# Cooperative Enantiomorphic-Site and Chain-End Chirality in Isoselective β‑Lactone Polymerization

**DOI:** 10.1021/jacs.6c14172

**Published:** 2026-10-22

**Authors:** Morgan S. Young, Yolanda Rusconi, Ethan B. Flanagan, Samantha N. MacMillan, Claudio De Rosa, Anne M. LaPointe, Giovanni Talarico, Geoffrey W. Coates

**Affiliations:** † Department of Chemistry and Chemical Biology, Baker Laboratory, 5922 Cornell University, Ithaca, New York 14853-1301, United States; ‡ 657489 Scuola Superiore Meridionale, Largo San Marcellino, 80138 Naples, Italy; § Department of Chemical Sciences, University of Naples Federico II, Via Cintia, 80126 Naples, Italy

## Abstract

Polymer properties are profoundly impacted by tacticity, necessitating an understanding of the origins of stereocontrol to make high-performance materials. Recently, we reported that controlled incorporation of stereoerrors into *trans*-poly­(3-hydroxy-2-methylbutyrate) (*trans*-PHMB) through the isoselective polymerization of *rac-trans*-3,4-dimethylpropiolactone (*trans*-DMPL) using racemic “sandwich” (BDI)Zn alkoxide complexes (where BDI = β-diketiminate) enables tuning of mechanical properties to match those of low-density polyethylene. However, the mechanism of stereocontrol of these catalysts was not explored. Herein, we report a thorough experimental and computational investigation uncovering a distinct manifestation of cooperative enantiomorphic-site and chain-end control, which we describe as site-mediated asymmetric chain-end control, where catalyst (Δ and Λ) and chain-end chiralities (*R*,*R* or *S*,*S*) work synergistically to influence the reaction’s stereoselectivity and rate. Experimental and computational data converge remarkably well, revealing that a Δ-catalyst with an (*R*,*R*)-chain end (Δ-*RR*) is isoselective 90% of the time. In contrast, a Δ-catalyst with an (*S*,*S*)-chain end (Δ-*SS*) is unselective. Detailed Density Functional Theory (DFT) investigations suggest molecular-level rationale for this mechanism of stereocontrol, demonstrating that chain positioning, monomer distortion, and ligand interactions work in conjunction to foster isoselectivity. Finally, DFT and kinetic modeling reveal that when polymerizing *rac-trans*-DMPL, the populations of Δ-*RR* and Δ-*SS* are equal and the high overall isoselectivity (*mm*% = 71–73%; where *mm*% is the percent of triads with a *meso* configuration across both ester linkages) of the catalyst is attributed to Δ-*RR* being faster than Δ-*SS*.

## Graphical abstract

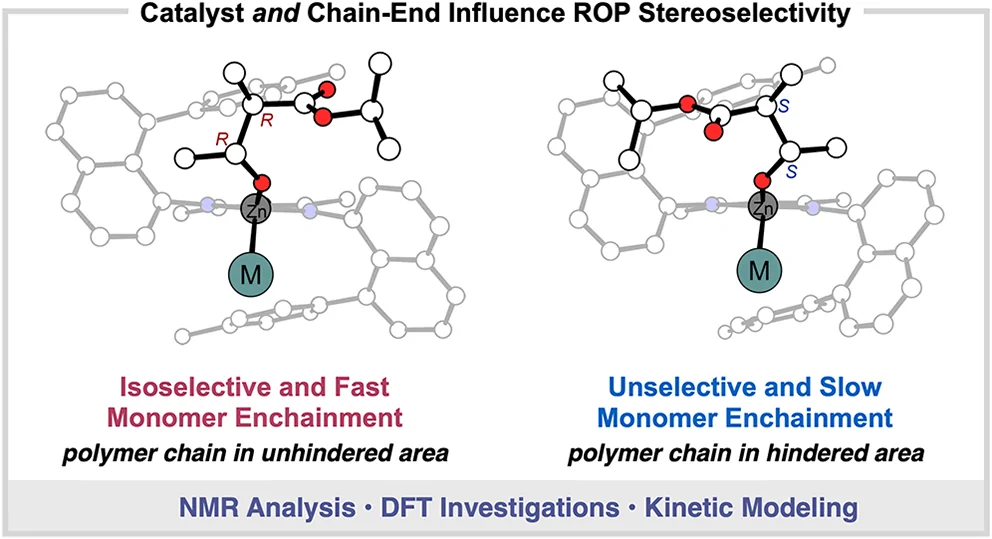

## Introduction

Nearly a century ago, Staudinger hypothesized that polymer main-chain stereoregularity, or tacticity, would have profound impacts on polymer properties.^1^ This hypothesis was later confirmed by Schildknecht and Natta with the discovery and characterization of isotactic poly­(isobutyl vinyl ether) and isotactic poly­(propylene) (*i*PP), respectively.^2–4^ Today, the semicrystallinity resulting from *i*PP’s high isotacticity has led to its widespread use, making it the second most produced plastic by volume worldwide.^5–8^ Since propylene is a prochiral monomer, significant effort has been put into understanding the mechanism of its stereospecific polymerization.^9–12^

Mechanistic studies of well-defined group 4 metallocene catalysts revealed relationships between ligand geometry, enantiofacial selectivity, chain-end positioning, and the stereochemical structure of the resulting polymer.^9–12^ This deep understanding has led to rational design of catalysts that yield high-performance materials.^10,11,13–20^ The same strategy is now being applied to the ring-opening polymerization (ROP) of lactones to access (bio)­degradable plastics and combat the accumulation of waste polyolefins.^21–25^ However, these polymerizations introduce an additional layer of complexity: chiral monomers, which can have a profound influence on catalyst selectivity. Development of a detailed mechanistic model for ROP stereoselectivity is essential for the rational design of catalysts that yield materials suitable for use as polyolefin alternatives.

Stereochemical control in lactone ROP is dictated by the free energy difference between the transition states leading to isotactic and syndiotactic enchainments.^26–28^ For achiral catalysts, four possible propagation pathways exist, two of which are unique by symmetry. For example, when an achiral catalyst polymerizes *rac-trans*-3,4-dimethylpropiolactone (*trans*-DMPL) there are two enantiomeric resting states: one with an (*R*,*R*)- and one with an (*S*,*S*)-chain end. Each of these resting states can incorporate an (*R*,*R*)- or (*S*,*S*)-*trans*-DMPL to make iso- or syndio-enchainments (Scheme 1A). In this case, there are two transition states contributing to observed stereoselectivity. However, chiral, *C*_2_-symmetric catalysts are often used to promote isoselective polymerization.^10,21,29–31^ Introduction of catalyst chirality doubles the number of propagation pathways to eight, where four are distinct by symmetry (Scheme 1B). Considering a single catalyst enantiomer, it can have either an (*R*,*R*)- or (*S*,*S*)-chain end. These diastereomeric resting states can incorporate an (*R*,*R*)- or (*S*,*S*)-*trans*-DMPL to give either iso- or syndio-enchainments. Owing to the unique combinations of chain end, monomer, and catalyst chiralities in each pathway, the overall tacticity depends on the relative free energies of all transition states, two leading to isotactic and two leading to syndiotactic enchainment.

**Scheme 1:**
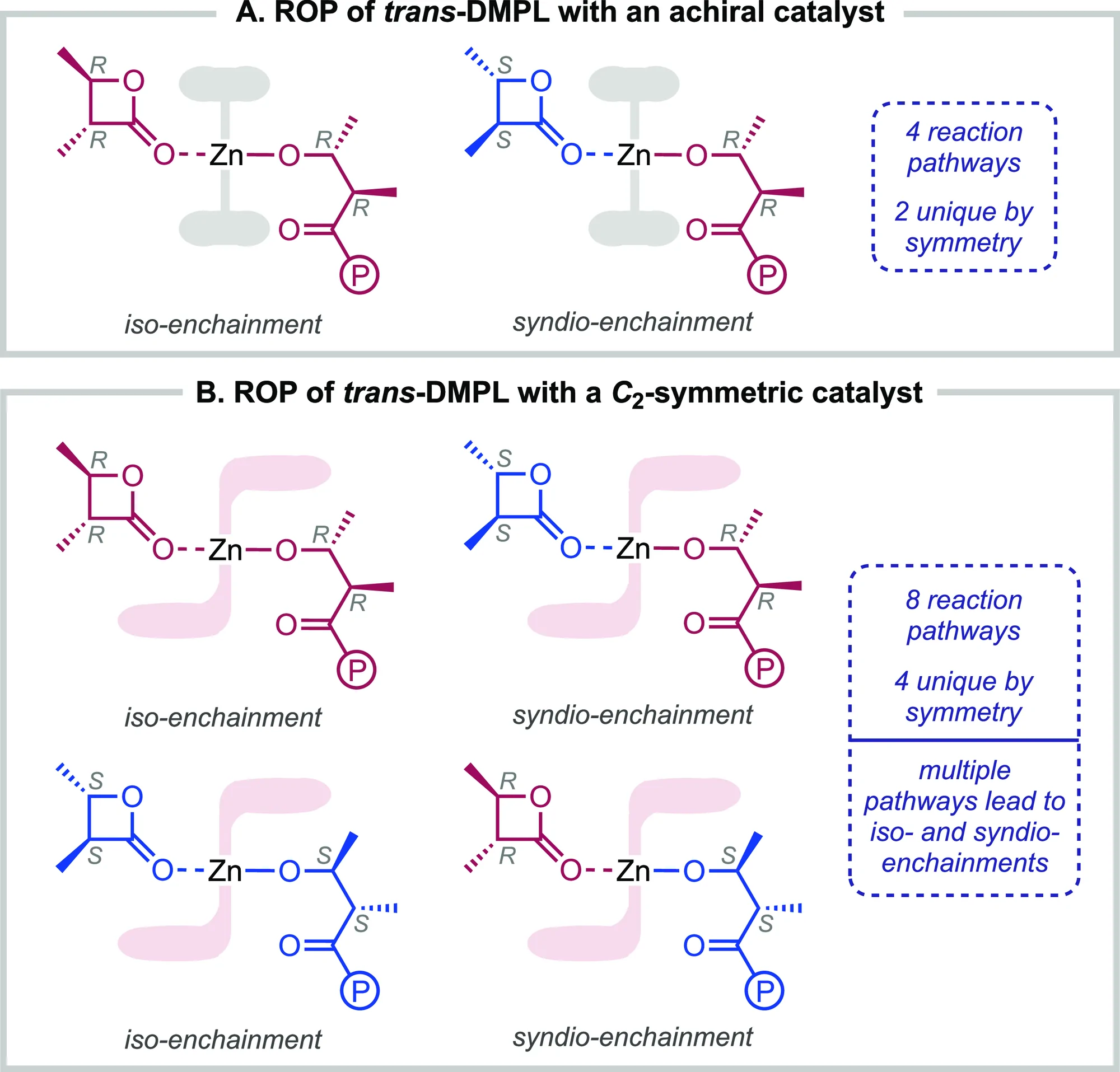
Stereochemical Pathways for ROP of *trans*-DMPL by a Zn-Based (A) Achiral Catalyst and (B) *C*_2_-Symmetric Catalyst

In most stereoselective polymerizations, the origin of stereocontrol is dominated by either the chain-end or enantiomorphic catalyst site. In chain-end control (CEC), the chirality of the terminal and/or penultimate polymer unit(s) determines the stereochemistry of the next inserted monomer.^32^ For isoselective polymerization, stereoerrors are propagated yielding stereoblock polymers (Scheme 2). On the other hand, enantiomorphic-site control (ESC) occurs when the chirality of the catalyst site dictates the stereochemistry of monomer insertion. In an isoselective polymerization, ESC yields enantioenriched polymers with isolated errors (Scheme 2). Although these paradigms may be independent, many polymerizations have shown synergistic contributions from both CEC and ESC.^33,34^ In ROP, this is often seen through catalyst-site rearrangement, exchange of polymer chain ends after insertion, and dormant/active catalyst states generated through chemoselective monomer incorporation.^35–44^ Although cooperative contributions from ESC and CEC have been recognized previously, the molecular origins of this cooperation can differ substantially between catalyst systems. The present work focuses on identifying the specific mechanistic origin of this interplay in the ROP of *trans*-DMPL by a racemic zinc alkoxide catalyst.

**Scheme 2:**
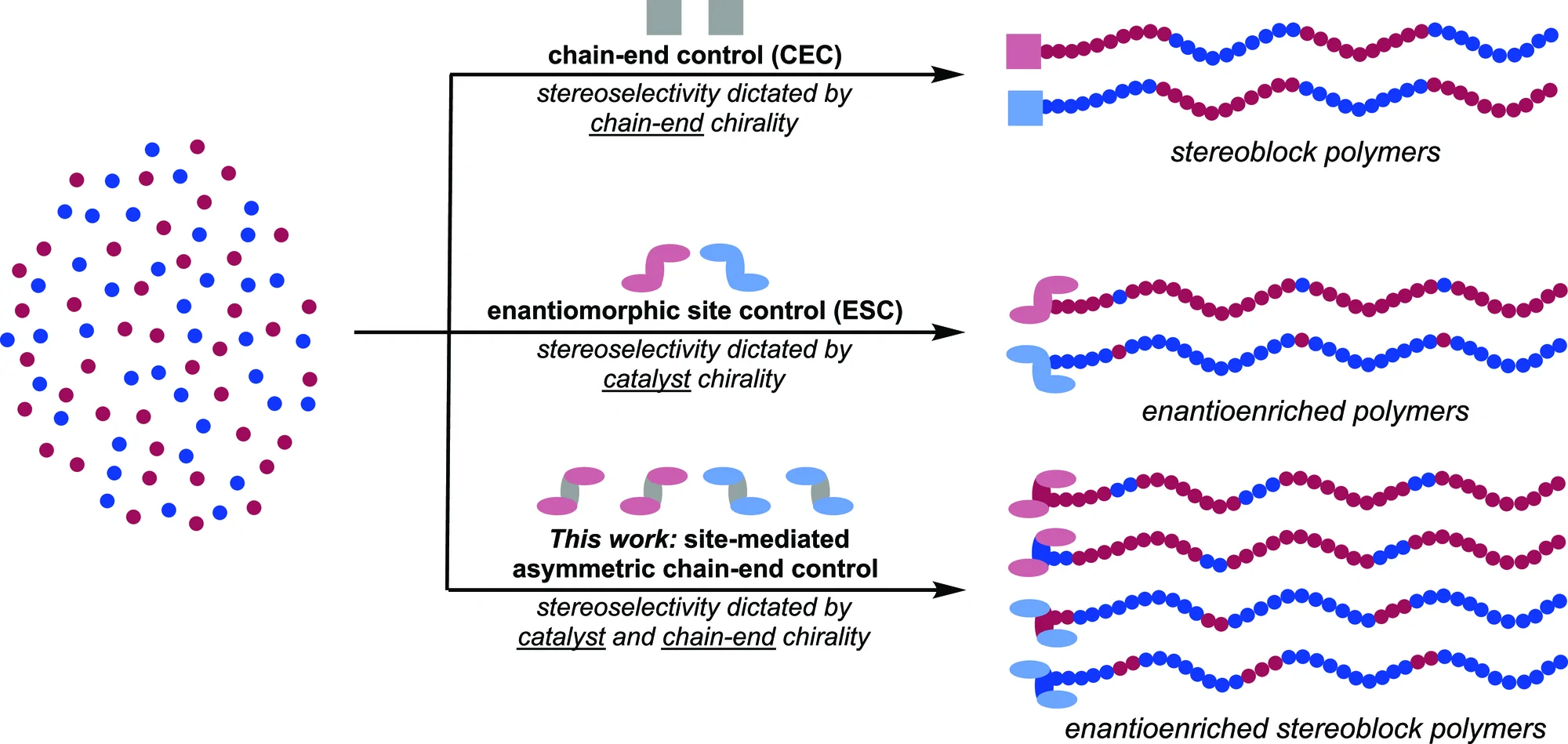
Mechanisms of Stereocontrol for Isoselective Polymerizations

Recently, we reported the synthesis of isoenriched *trans*-poly­(3-hydroxy-2-methylbutyrates) (*trans*-PHMBs) as a thermally stable, biodegradable alternative to low-density polyethylene (LDPE).^45^ To access these materials, we designed new *C*_2_-symmetric *rac*-(^Ar^BDI*)­ZnO^*i*^Pr (where BDI = β-diketiminate and Ar = 8-arylnaphthylamine) complexes that isoselectively polymerize *rac-trans*-DMPL, which can be scalably synthesized through sequential epoxidation and catalytic carbonylation of *cis*-2-butene.^46–48^ We were particularly interested in (BDI)Zn systems because they are known for being single-site, living β-lactone ROP catalysts capable of reaching high molecular weights with quantitative monomer conversion on reasonable time scales.^49,50^ To create a stereoselective BDI system, we used 8-arylnaphthylamines, developed by Daugulis, Brookhart and co-workers, to synthesize chiral “sandwich” BDI ligands with axial steric bulk to prevent rapid chain transfer between catalysts.^51,52^ This industrially relevant, cost-efficient approach yielded a racemic catalyst able to quantitatively convert *rac-trans*-DMPL into semicrystalline material. Mechanistically, this is a challenging system to study, having two catalyst enantiomers, each with two iso- and two syndio-enchainment pathways. Given the favorable thermal and mechanical properties achieved in our prior work, we sought to understand the mechanism of stereocontrol.

Herein, we report a combined experimental and computational approach to study the mechanism of the new *rac*-(^Ar^BDI*)­ZnO^*i*^Pr complexes in the ROP of *trans*-DMPL. We investigated the roles of catalyst (ESC) and chain end (CEC) chirality in influencing the overall isoselectivity. Kinetic studies establish the nature of the active species and the rate law which, combined with thorough NMR analysis and detailed Density Functional Theory (DFT) calculations, provide a molecular-level description of the catalytic cycle and the origin of stereocontrol. The combined experimental and computational studies uncover that catalyst and chain-end chirality work synergistically to impart isoselectivity through a specific mode of cooperative ESC and CEC that we term site-mediated asymmetric chain-end control, producing enantioenriched stereoblock polymers (Scheme 2).

## Results and Discussion

### Activity and Speciation of *rac*-(^4‑Me^BDI*)­ZnO^*i*^Pr (*rac*-**1**)

Among the *rac*-(^Ar^BDI*)­ZnO^*i*^Pr catalysts investigated, we selected *rac*-(^4‑Me^BDI*)­ZnO^*i*^Pr (Ar = 8-(4-methylphenyl)-naphthyl-1-amine; *rac*-**1**) as the optimal candidate for further mechanistic studies because it showed the best combination of activity and isoselectivity.^45^ *rac*-(^4‑Me^BDI*)­ZnO^*i*^Pr demonstrates nearly quantitative conversion (>96%) and high isoselectivity (*mm*% = 71–73%; where *mm*% is the percent of triads with a *meso* configuration across both ester linkages) over a wide range of [*trans*-DMPL]_0_:[*rac*-**1**]_0_ ratios spanning between 200:1 and 5000:1 (Table 1, entries 1–8). The resulting polymers have the targeted molecular weights with narrow molecular weight distributions (*Đ* = 1.04–1.08). After full conversion of monomer, the molecular weight and *Đ* remain constant, indicating that the catalyst does not promote transesterification or elimination reactions during or after propagation (Figure S6). Furthermore, addition of 1–10 equivalent(s) of isopropanol as a chain transfer agent (CTA; Table 1, entries 9–11) enabled synthesis of *trans*-PHMB with tunable molecular weights with smaller Zn loadings. Remarkably, as the equivalents of CTA were increased, there was no influence on the resulting polymer isotacticity. This suggests that (1) the metal-bound ligand is resistant to being protonated off the metal at these alcohol concentrations and (2) the rate of chain transfer is slower than that of propagation. It is worth noting that a slightly broadened dispersity (*Đ* = 1.08–1.15) is observed when using CTA, but this is still quite narrow for a β-lactone polymerization.

**Table 1:**
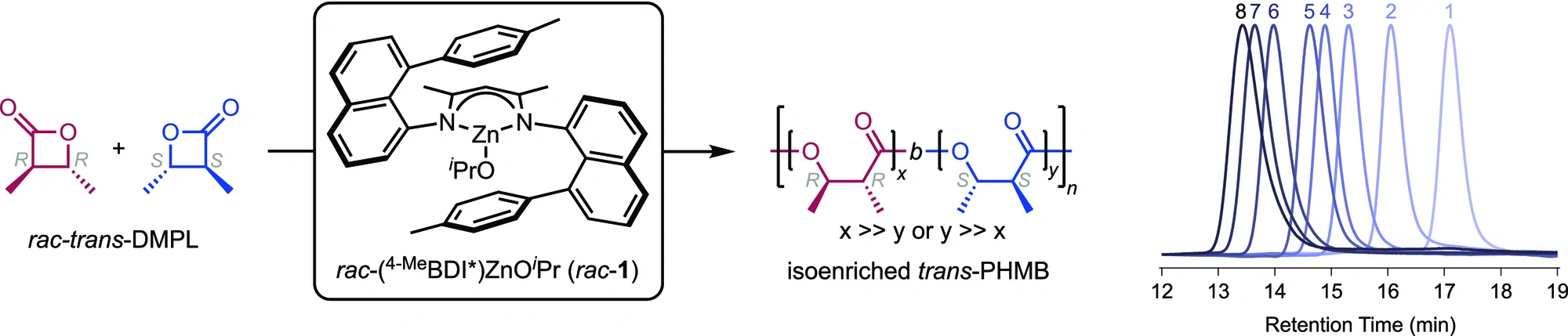
Polymerization of *rac-trans*-DMPL by *rac*-**1**

Entry^a^	[M]_0_:[*rac*-**1**]_0_:[^*i*^PrOH]	*t*_rxn_ (h)	conv. (%)^b^	*M*_n,theo_ (kDa)	*M*_n,SEC_ (kDa)^c^	*Đ* ^c^	*mm*%^d^
1	200:1:0	18	>99	20.0	25.4	1.06	71
2	500:1:0	18	>99	50.1	56.0	1.08	71
3	1000:1:0	18	>99	100.1	105.8	1.06	71
4	1500:1:0	18	>99	150.2	149.6	1.04	71
5	2000:1:0	18	96	192.2	185.9	1.04	71
6	3000:1:0	48	98	294.4	303.7	1.06	73
7	4000:1:0	96	>99	400.5	387.1	1.06	72
8	5000:1:0	96	97	485.6	454.1	1.06	71
9	500:1:1	18	>99	25.0	29.6	1.08	72
10	500:1:5	18	>99	8.3	11.3	1.15	72
11	500:1:10	18	>99	4.6	6.6	1.12	73

a Polymerizations were performed with 1.00 mmol *trans*-DMPL, [*trans*-DMPL]_0_ = [M]_0_ = 4 M in PhMe, *T*_rxn_ = 25 °C.

b Determined by ^1^H NMR spectroscopic analysis comparing the relative integration of polymer and residual monomer.

c Determined by SEC in THF at 30 °C, calibrated relative to monodisperse polystyrene standards.

d Determined by ^13^C­{^1^H} NMR spectroscopic analysis.

The exceptional control demonstrated by *rac*-**1** prompted an investigation into its speciation as both an isolated complex and in its active form during polymerization, as (BDI)Zn alkoxides are known to exist as monomers and dimers.^49,53–55^ Complex *rac*-**1** crystallizes in the centrosymmetric, monoclinic space group *P*2_1_/*n* as a mixture of two cocrystallized enantiomers: Δ (clockwise) and Λ (counterclockwise). Single-crystal X-ray diffraction analysis revealed that *rac*-**1** adopts a mononuclear structure with pseudo-*C*_2_ symmetry around the metal center creating a chiral catalytic pocket (Figure 1). A trigonal planar coordination geometry around the zinc center is observed; although rare for a zinc alkoxide complex, such geometries are not unprecedented.^56–60^ The zinc center is chelated to the BDI ligand through the two nitrogen atoms (N1 and N2) and the isopropoxide group via the oxygen. In the solution state, a single set of peaks is observed in the ^1^H NMR spectrum, suggesting a monomeric structure (Figure S14). The presence of the *p*-tolyls in *rac*-**1** likely prevents dimerization, stabilizing the mononuclear structure through π–π interactions between the 8-aryl groups and the diimine backbone, as confirmed by the positive Gibbs energy of dimerization and noncovalent interaction (NCI) analysis (*vide infra* for computational details and see Figures S26 and S27). Further supporting the presence of these NCIs, the chelate ring in the crystal structure is canted toward the *p*-tolyl groups of the ligand, likely to enhance π–π stacking. Considerable interactions were also observed between the *p*-tolyl groups of the ligand and the BDI backbone in solution by ^1^H NMR spectroscopy. In *rac*-**1**, the backbone methine is significantly shifted upfield to 3.94 ppm compared to the backbone methine in [(^*i*Pr^BDI)­ZnO^*i*^Pr]_2_ (^*i*^Pr = 2,6-diisopropylaniline) at 4.92 ppm suggesting the methine is situated within the aromatic currents of the 8-aryl rings (Figure S14).

**Figure 1:**
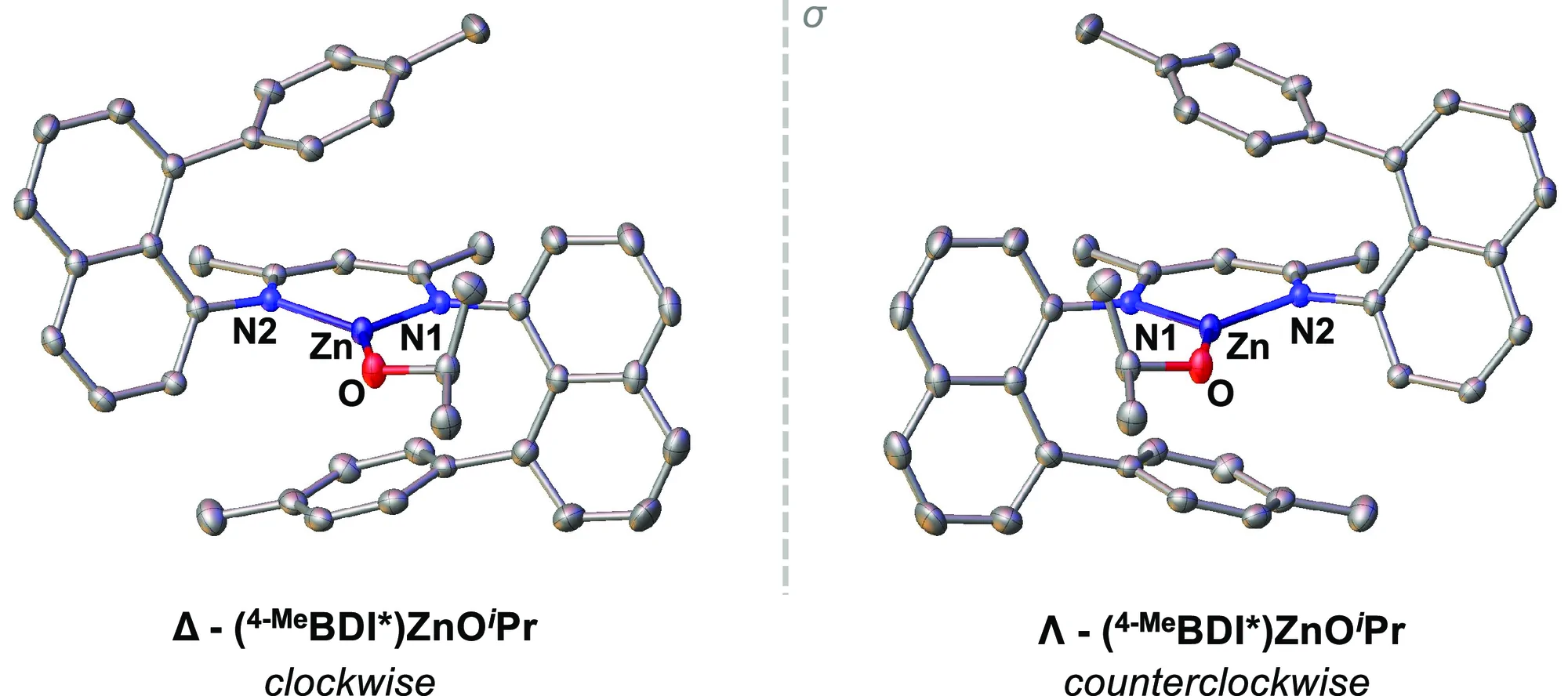
ORTEP drawing of *rac*-**1** with thermal ellipsoids drawn at the 50% probability level. CCDC 2563348 Selected bond distances (Å) and angles (deg): Zn–O, 1.8304(10); Zn–N(1), 1.9425(11); Zn–N(2), 1.9316(11); O–Zn–N(1), 129.27(5); O–Zn–N(2), 130.14(4); N(2)–Zn–N(1), 100.46(5); C(40)–O–Zn, 119.53(8).

To elucidate the catalyst’s speciation during polymerization, the reaction orders in monomer and zinc were determined through a series of kinetic experiments. Polymerizations were conducted at different [*trans*-DMPL]_0_:[catalyst]_0_ ratios (200–600) with a fixed [*trans*-DMPL]_0_ of 4.0 M, and the initial rates of monomer conversion were monitored over time. A first-order dependence on monomer concentration was observed in each case (Figure 2A). The order in zinc was determined by plotting ln­(*k*_app_) versus ln­([Zn]). The slope of the fitted line is 1.05 ± 0.03, confirming a first-order dependence on zinc (Figure 2B), giving the overall rate law −d­[*trans*-DMPL]/d*t* = *k*_p_[*trans*-DMPL]­[*rac*-**1**]. A similar first order dependence on monomer and catalyst was reported for the ROP of β-butyrolactone by [(^*i*Pr^BDI)­ZnO^*i*^Pr]_2_, despite its dimeric precatalytic state.^49^ In summary, *rac*-**1** is mononuclear in its solid- and solution-state structures as well as in its active form during polymerization.

**Figure 2:**
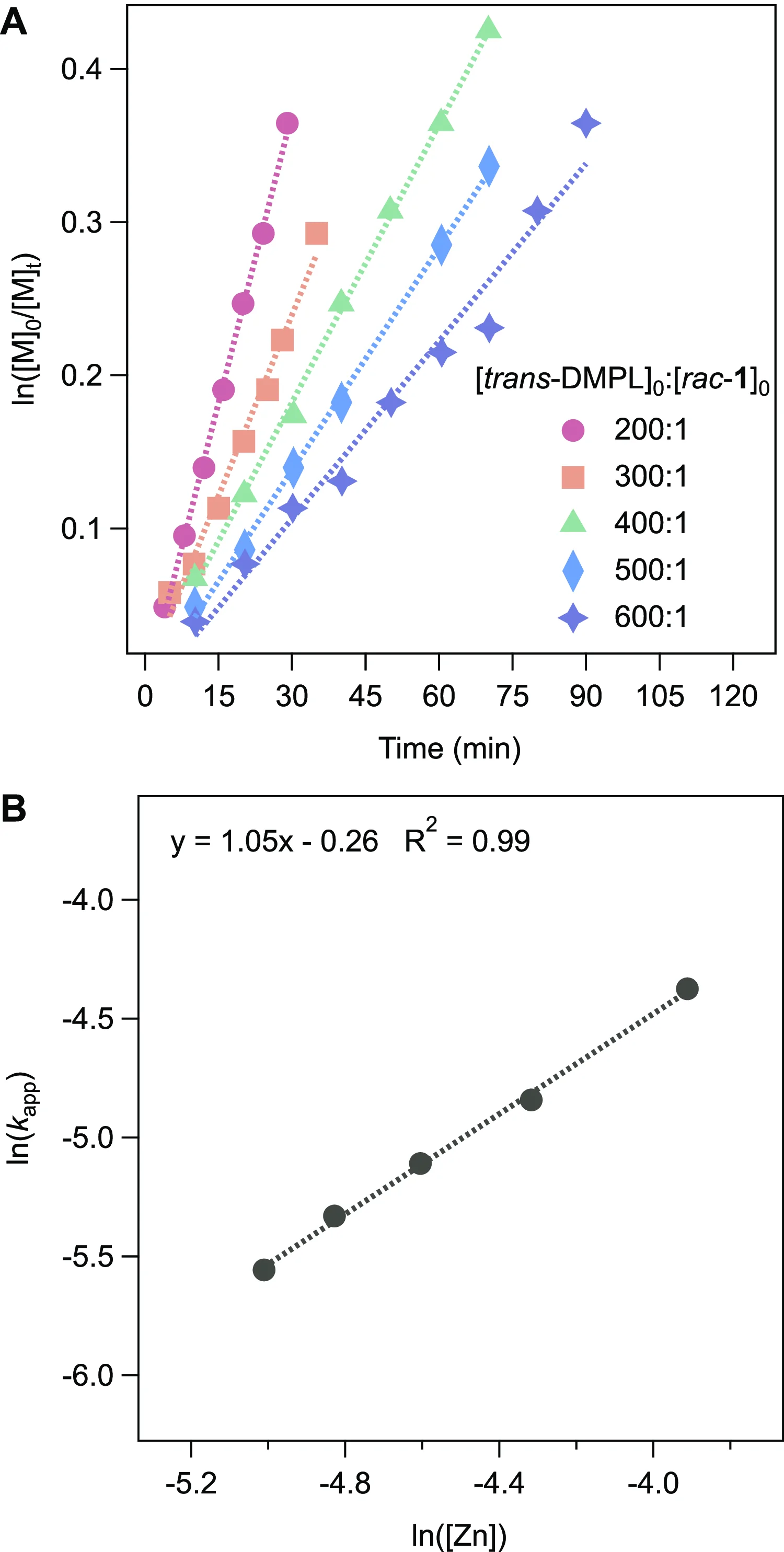
(A) First-order kinetic plots for the polymerizations of different ratios of [*trans-*DMPL]_0_:[*rac*-**1**]_0_ ([*trans*-DMPL]_0_ = 4.0 M) and (B) plot of ln­(*k*_app_) vs ln­([Zn]).

### ^13^C­{^1^H} NMR Analysis of Polymer Microstructure

Next, we analyzed the distribution of stereoerrors to decipher the mechanism of *rac*-**1**’s isoselectivity. To assess this, we employed the Bernoulli CEC triad test for isoselective polymerizations using the integrations of the triad-centered peaks in the ^13^C­{^1^H} NMR spectra (Figure 3), where *B* = 4­[*mm*]­[*rr*]/([*rm*] + [*mr*])^2^.^32,45^ When *B* = 1, the system operates through a perfect CEC mechanism, while a value of *B* > 1 indicates elements of ESC. Applying the Bernoulli test to a polymer produced by *rac*-**1** (Table 1, entry 1), a value of *B* = 2.6 is obtained, indicating a hybrid ESC and CEC mechanism. This result prompted a thorough experimental and computational mechanistic investigation to uncover the origins and relative contributions of ESC and CEC that dictate the isoselectivity of *rac*-**1**.

**Figure 3:**
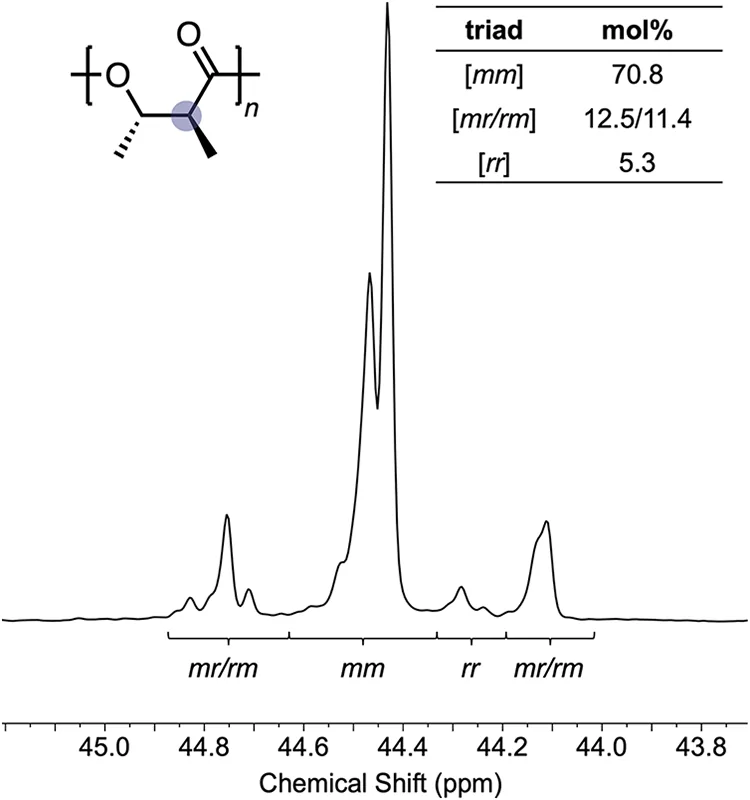
Portion of ^13^C­{^1^H} NMR spectrum of the *trans*-PHMB from Table 1, entry 1 showing the triad-centered peaks of the α-carbon.

We began by creating a model to define the probabilities of racemic monomer insertions as a function of Δ catalyst resting states (Scheme 3; see SI Section 3.2 for the full details). The Δ catalyst has two diastereomeric resting states: either with an (*R*,*R*)-PHMB chain end (Δ-*RR*) or an (*S*,*S*)-PHMB chain end (Δ-*SS*). A Δ-*RR* catalyst can insert an (*R*,*R*)- or (*S*,*S*)-*trans*-DMPL. The probabilities of these two insertions are defined as *P*_*RR*_^Δ*RR*^ and *P*_*SS*_^Δ*RR*^, respectively, where the subscript denotes the stereochemistry of the incoming monomer and the superscript indicates the resting state of the starting catalyst. The sum of *P*_*RR*_^Δ*RR*^ and *P*_*SS*_^Δ*RR*^ is equal to one and their ratio (*P*_*RR*_^Δ*RR*^/*P*_*SS*_^Δ*RR*^) is equal to the ratio of rate constants *k*_*RR*→*RR*_^Δ^/*k*_*RR*→*SS*_^Δ^, assuming racemic monomer. Using the Eyring equation, this ratio can be converted into the free energy difference between the rate-determining transition states (Δ*G*_*RR*→*SS*_^‡^ – Δ*G*_*RR*→*RR*_^‡^ = ΔΔ*G*_stereo_^‡^). The subscripts of the rate constants denote the starting and ending stereochemical configuration of the polymer chain end. In a similar fashion, *P*_*RR*_^Δ*SS*^, *P*_*SS*_^Δ*SS*^, *k*_*SS*→*RR*_^Δ^, *k*_*SS*→*SS*_^Δ^, Δ*G*_*SS*→*RR*_^‡^ and Δ*G*_*SS*→*SS*_^‡^ can be applied to Δ-*SS*. These probability terms do not factor in population or rate differences between Δ-*RR* and Δ-*SS*. To account for this, we introduced the correction factors *P*_rxn_^Δ*RR*^ and *P*_rxn_^Δ*SS*^ which are the probability of any Δ reaction occurring at Δ-*RR* and Δ-*SS*, respectively, and the sum of these two terms is one. By symmetry, these probabilities, rate constants and activation energies can be applied to the Λ catalyst by inverting all stereochemical centers on the incoming monomer and chain.

**Scheme 3:**
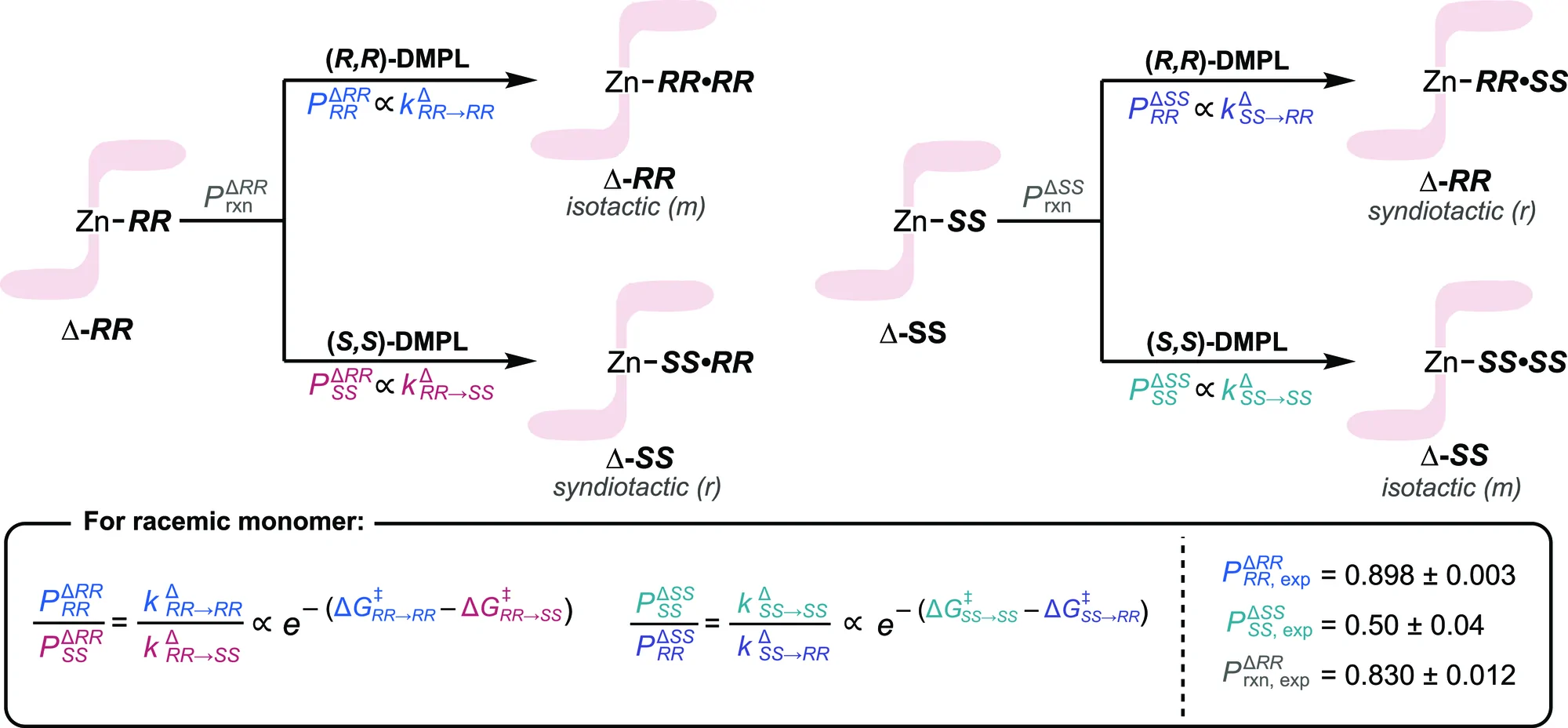
NMR Model and Experimentally Determined Probabilities

To determine these probabilities from experimental data, we correlated these terms with the measurable integrations of the triad-centered peaks of the α-carbon in the ^13^C­{^1^H} NMR spectra of PHMB (Scheme S1). This created a system of equations with two unknown probabilities, *P*_*RR*_^Δ*RR*^ and *P*_*SS*_^Δ*SS*^, and four measurables, [*mm*], [*mr*], [*rm*], and [*rr*]. Using the least-squares method, the errors on [*mm*], [*mr*], [*rm*], and [*rr*] were minimized for entries 1–8 in Table 1 (assuming Δ-catalyst favors (*R*,*R*)-*trans*-DMPL insertion; *vide infra* for computational support). By averaging the solutions (Table S3), the following values were obtained: *P*_*RR*_^Δ*RR*^ = 0.898 ± 0.003, *P*_*SS*_^Δ*SS*^ = 0.50 ± 0.04 and *P*_rxn_^Δ*RR*^ = 0.830 ± 0.012 (Scheme 3; uncertainty is presented as the standard deviation of the mean).

These results indicate that Δ-*RR* has a large preference for inserting (*R*,*R*)-*trans*-DMPL monomer forming an isotactic enchainment, and this occurs during 90% of all insertions at Δ-*RR*. On the other hand, Δ-*SS* is unselective, inserting each enantiomer 50% of the time. Furthermore, the *P*_rxn_^Δ*RR*^ indicates that, with racemic monomer, 83% of Δ reactions occur at Δ-*RR* catalyst. The higher isoselectivity of Δ-*RR* means *rac*-**1** produces polymer chains with an enantioenriched stereoblock microstructure. The Δ catalyst produces *trans-*PHMB with longer blocks of (*R*,*R*) repeat units (average block length = 1/*P*_*SS*_^Δ*RR*^ = 10 units) with shorter blocks of (*S*,*S*) repeat units (average block length = 1/*P*_*RR*_^Δ*SS*^ = 2 units). Meanwhile, the Λ catalyst will produce equal and opposite polymer chains composing of longer blocks of (*S*,*S*)-PHMB and shorter blocks of (*R*,*R*)-PHMB. In the polymerization of *trans*-DMPL by *rac*-**1**, it is not the catalyst or chain-end chirality alone that dictates the overall selectivity, but rather the unique combination of both. This is unlike classical CEC and ESC where either the chain end or catalyst site drive stereocontrol. In this system, all elements of chirality act cooperatively to produce a stereochemical interplay that is distinct from previously described examples of ESC/CEC cooperation. We refer to this mechanistic picture as site-mediated asymmetric chain-end control (Scheme 2).

While the NMR model provides significant insight into the mechanism of stereocontrol by *rac*-**1**, it cannot relate the relative populations and rates of Δ-*RR* and Δ-*SS* to each other. In particular, the model indicates that, with racemic monomer, 83% of Δ reactions occur at a Δ-*RR*, but it is unclear if Δ-*RR* is a faster catalyst than Δ-*SS* (i.e., *k*_*RR*→*RR*_^Δ^ + *k*_*RR*→*SS*_^Δ^ > *k*_*SS*→*SS*_^Δ^ + *k*_*SS*→*RR*_^Δ^), if Δ-*RR* is more populous than Δ-*SS* ([Δ-*RR*] > [Δ-*SS*]), or some combination of both. To answer this question and gain a deeper molecular-level description of why Δ-*RR* is highly selective and Δ-*SS* is unselective, we turned to DFT calculations.

### DFT Investigations of *rac*-**1** Isoselectivity

DFT calculations were performed using the Gaussian 16 package.^61^ Geometry optimizations and frequency calculations were carried out at the B3-D3BJ^62–64^ /SDD^65^­(Zn)/Def2SVP^66^ level. Single-point energies were refined at the PBE0-D3BJ/SDD­(Zn)/6-311G­(d,p)^68,69^ level using the CPCM^70^ solvation model (toluene). Additional computational details are reported in the Supporting Information.

All possible combinations of the chiral elements involved in the reaction were considered to provide a comprehensive description of the stereochemical outcomes of the polymerization. The Δ configuration of *rac*-**1** was arbitrarily selected. Due to the *C*_2_ symmetry of the catalyst, this configuration features two homotopic coordination sites (chemically equivalent binding sites). Both (*R*,*R*)- and (*S*,*S*)-DMPL were examined, each undergoing attack on both the *re* and *si* enantiofaces. Following initiation, two propagating chains are generated: those with (*R*,*R*)- and (*S*,*S*)-chain ends (see Scheme S2). The catalytic cycle begins with coordination of the monomer to the zinc center (INT1), enabling nucleophilic attack of the initiator on the monomer carbonyl group via TS1, followed by formation of intermediate INT2. Subsequent ring-opening through TS2 yields the propagating species INT3 (Δ-*RR* or Δ-*SS*) (Figure 4, top).

**Figure 4:**
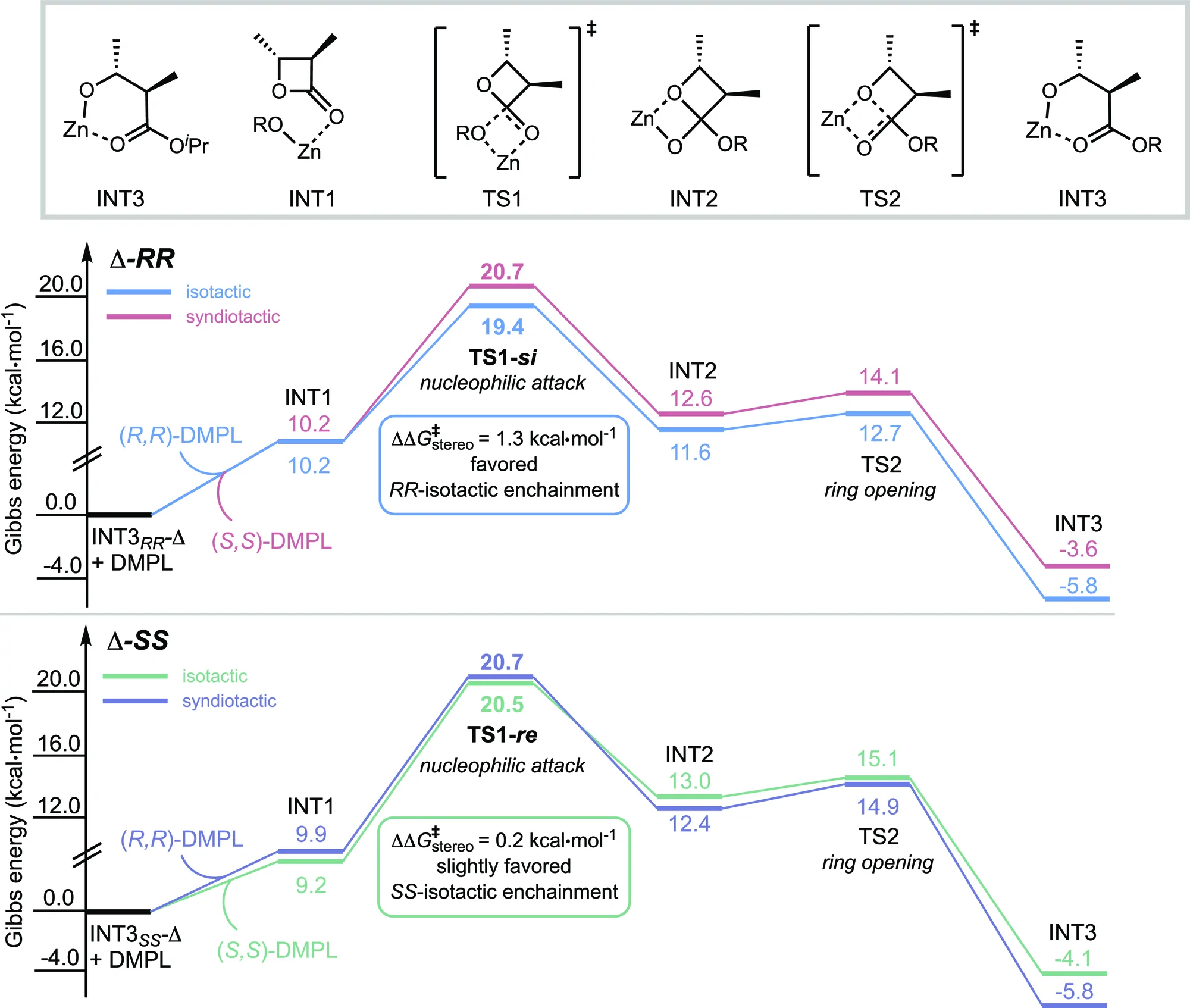
Minimum energy paths for the propagation of the ROP of *rac-trans*-DMPL promoted by Δ-*RR* (middle) and Δ-*SS* (bottom). The pathways leading to isotactic enchainments are reported in blue and green while the ones leading to syndiotactic enchainments in red and purple for Δ-*RR* and Δ-*SS*, respectively. Catalytic intermediates and transition states for *RR*-isotactic enchainment are shown at the top.

We first examined the insertion of a single monomer unit initiated by the O^*i*^Pr group on Δ-**1** (Figure S28). Nucleophilic attack on the *si* enantioface was identified as the rate-determining step (RDS), with activation barriers of 16.3 and 16.5 kcal·mol^–1^ for (*S*,*S*)- and (*R*,*R*)-DMPL, respectively. The small free energy difference between the rate-determining transition states (ΔΔ*G*_stereo_^‡^ = 0.2 kcal·mol^–1^) indicates negligible stereocontrol during initiation. Ring-opening was found to be faster than nucleophilic attack, consistent with the high ring strain of four-membered lactones. The resulting intermediates, INT3_*RR*_-Δ (Δ-*RR*) and INT3_*SS*_-Δ (Δ-*SS*), are isoenergetic (−10.9 kcal·mol^–1^), adopting a tetrahedral geometry around zinc, coordinated to the alkoxide and carbonyl oxygen atoms of the growing chain.

We then studied the second *rac-trans*-DMPL insertion promoted by INT3_*RR*_-Δ (Δ-*RR*) and INT3_*SS*_-Δ (Δ-*SS*) (Figure 4). First, a conformational analysis of the growing chain was conducted to identify its minimum-energy conformations. Two-dimensional potential energy surface scans were obtained on two key dihedral angles (Scheme S4), which are critical for defining the conformational landscape of the propagating species. The resulting energy maps, along with a detailed table summarizing the identified global minima, are provided in Figures S29–S30 and Table S9. Optimization of the transition states was subsequently conducted, including the growing chain in various minimum-energy conformations to ensure proper placement within the catalytic pocket. From all the transition states identified, the lowest-lying structures were selected and are reported in the minimum energy pathways (MEPs, Figure 4).

First, propagation promoted by INT3_*RR*_-Δ (Δ-*RR*) was investigated (Figure 4, middle). The energy profile closely resembles that of the initial insertion, with nucleophilic attack on the *si* enantioface (see Table S7 for *re* enantioface energetic values) as the RDS and fast ring-opening. Notably, a preference of 1.3 kcal·mol^–1^ for sequential incorporation of (*R*,*R*)-DMPL was calculated (Δ*G*_*RR*→*RR*_^‡^ = 19.4 vs Δ*G*_*RR*→*SS*_^‡^ = 20.7 kcal·mol^–1^). This ΔΔ*G*_stereo_^‡^ translates to a *P*_*RR*,theo_^Δ*RR*^ = 0.90 which is in excellent agreement with the experimentally observed *P*_*RR*,exp_^Δ*RR*^ of 0.898 ± 0.003.

Closer inspection of the stereodetermining transition states provided insight into the origin of selectivity (Figure 5). The lowest energy chain conformation is the one in which the (*R*,*R*)-chain is positioned within a less hindered region of the catalytic pocket, avoiding steric repulsions. Moreover, preference for the *si* enantioface (Table S7) arises from minimization of steric interactions with the methyl substituents of the growing chain. Specifically, *si-*attack on (*R*,*R*)-DMPL places the C2 methyl group away from both the ligand framework and the chain, while the methyl substituent at C3 remains sufficiently distant from the propagating chain to avoid significant steric repulsion. In contrast, *si*-attack on (*S*,*S*)-DMPL directs the C2 methyl group toward the naphthyl ring of the ligand (3.5 Å), resulting in an unfavorable interaction (Figure 5, bottom). This monomer-ligand interaction induces significant distortion of the monomer in the transition state, which can be quantified by the dihedral angle τ (C1–C2–C3–O). In the minimum-energy conformation of DMPL, τ is ± 1.8°, whereas in the transition states it is −2.0° for (*R*,*R*)-DMPL and 9.3° for (*S*,*S*)-DMPL, indicating substantial deviation of the latter from the ideal geometry (Figure 5). To quantify this effect, Activation Strain Model (ASM) analysis^71,72^ was performed by dividing the transition states (TS1_*RR*→*RR*_ and TS1_*RR*→*SS*_) into two fragments: monomer and catalyst coordinated to the growing chain (Figure S31). This approach enables quantitative evaluation of the energetic contributions associated with deformation of each fragment in the transition state, thereby providing insight into the origin of stereoselectivity. The deformation energy of the monomer is significantly higher for (*S*,*S*)-DMPL than for (*R*,*R*)-DMPL (19.9 vs 17.2 kcal·mol^–1^), corresponding to ΔΔ*E*_Strain(Mon)_ = 2.7 kcal·mol^–1^, confirming that monomer distortion contributes substantially to stereoselectivity (Table 2). Interestingly, the same analysis performed on the RDSs of the initiation (in the absence of a chiral growing chain) revealed a much lower ΔΔ*E*_Strain(Mon)_ (0.6 kcal·mol^–1^, Table S8) with a lack of stereoselectivity (ΔΔ*G*_stereo_^‡^ = 0.2 kcal·mol^–1^ with O^*i*^Pr). The increase of stereoselectivity in the presence of a chiral growing chain is like that observed for propylene polymerization promoted by Group 4 *ansa*-metallocenes. Indeed, several studies have shown that a nonstereoselective propylene insertion into the M-alkyl bond is observed in the presence of a short, achiral chain, such as −CH_3_. This opened the opportunity for the “chiral growing-chain orientation” model to explain stereoselective propylene polymerization.^73–76^

**Figure 5:**
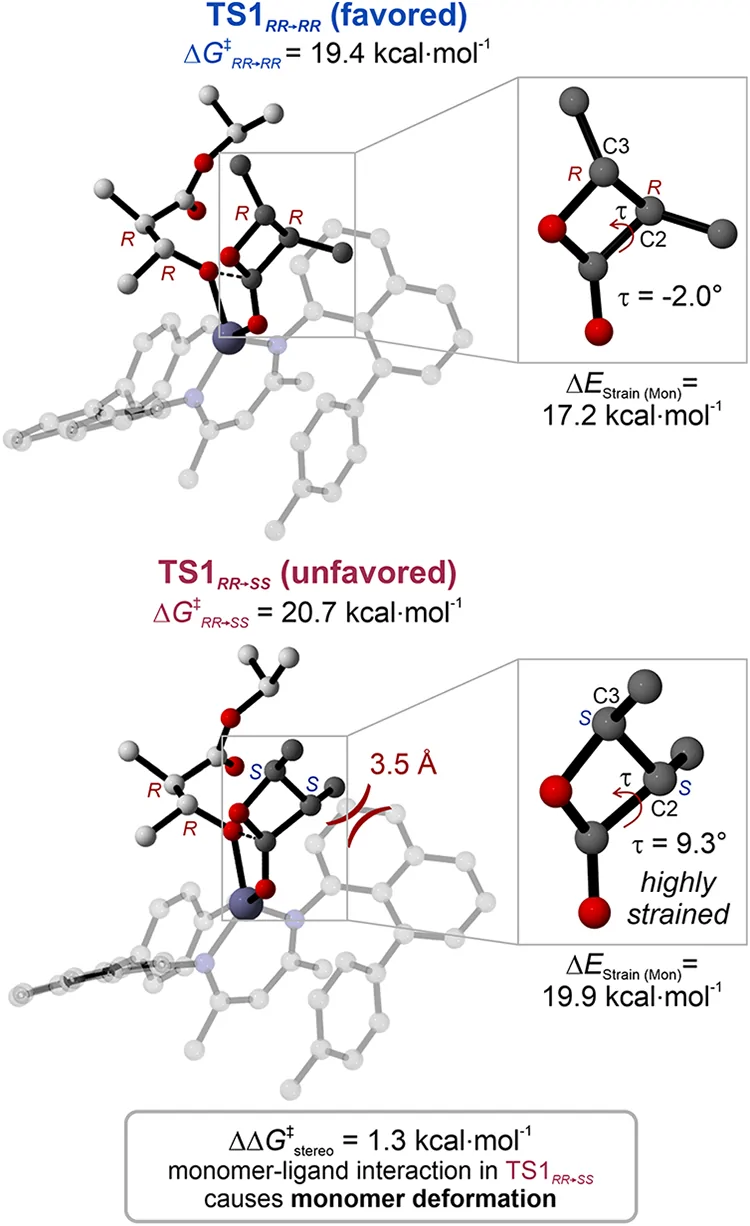
Optimized structures of stereoselectivity determining steps in the propagation step of the ROP of *rac-trans*-DMPL promoted by Δ-*RR* and monomer deformation analysis.

**Table 2:**
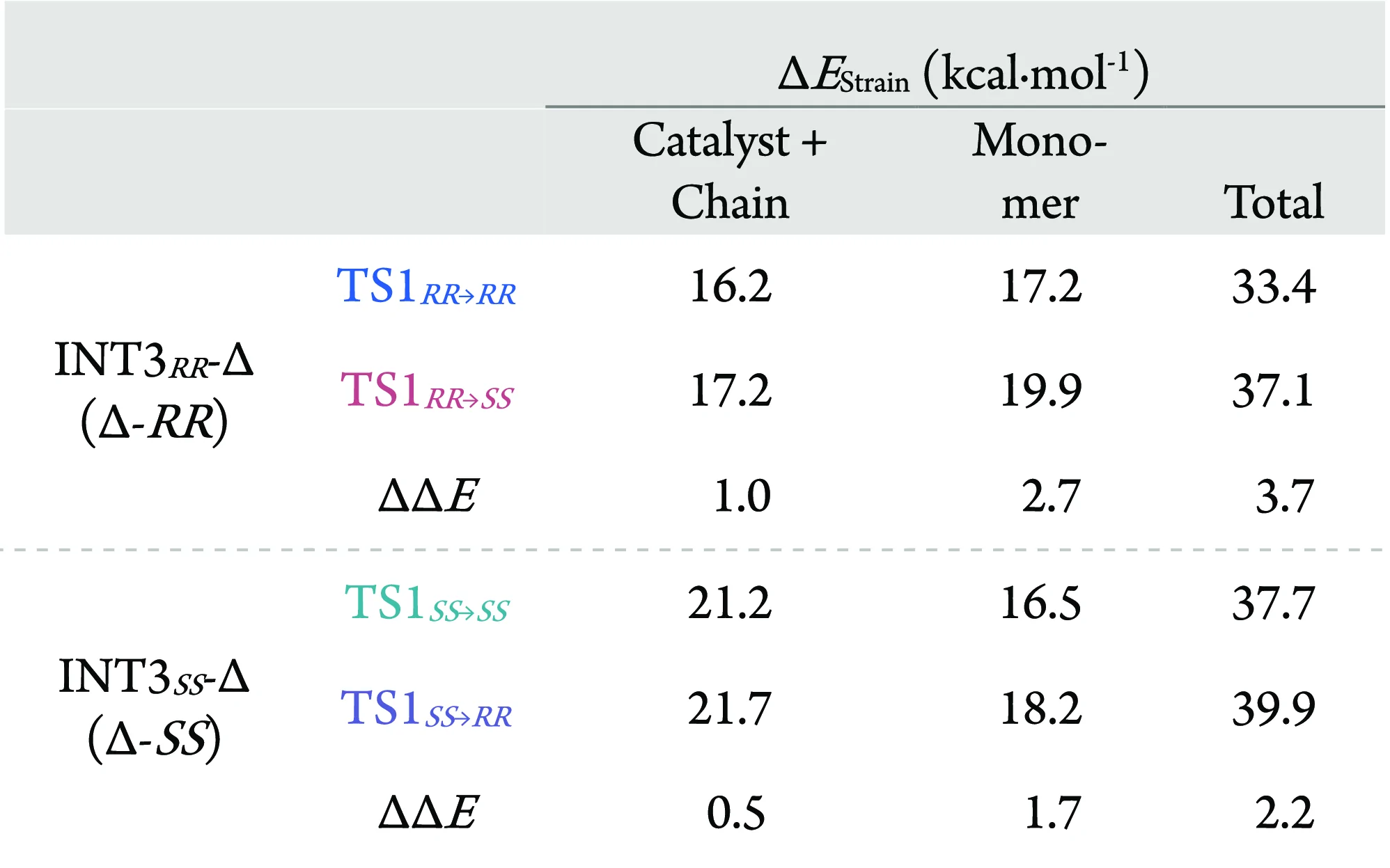
ASM Results for Enantioselectivity-Determining TSs in the Propagation of the ROP of *rac-trans*-DMPL Promoted by Δ-*RR* (Top) and Δ-*SS* (Bottom)

Next, the pathway originating from INT3_*SS*_-Δ (Δ-*SS*) was examined (Figure 4, bottom). The RDS is still nucleophilic attack, although higher activation barriers were calculated compared to the Δ-*RR* pathway and minimal stereoselectivity (ΔΔ*G*_stereo_^‡^ = 0.2 kcal·mol^–1^), resulting in only a slight preference for *SS*-isotactic enchainment (Δ*G*_*SS*→*SS*_^‡^ = 20.5 vs Δ*G*_*SS*→*RR*_^‡^ = 20.7 kcal·mol^–1^). This correlates to a *P*_*SS*,theo_^Δ*SS*^ of 0.58 which is in good agreement with *P*_*SS*,exp_^Δ*SS*^ (0.50 ± 0.04). The (*S*,*S*)-chain adopts a conformation opposite to that of the (*R*,*R*)-chain. In this case, attack on the *re* enantioface is favored for both monomers (see Table S7 for *si* enantioface energetic values), minimizing interactions with the growing chain (Figure S32). ASM analysis revealed a smaller overall strain energy difference between the two competing transition states than that calculated for the Δ-*RR* pathway (ΔΔ*E*_Strain(Tot)_ = 2.2 kcal·mol^–1^ for Δ-*SS* and 3.7 kcal·mol^–1^ for Δ-*RR*, Table 2), with monomer deformation remaining the main source of destabilization for TS1_*SS*→*RR*_ (ΔΔ*E*_Strain(Mon)_ = 1.7 kcal·mol^–1^). However, despite the non-negligible strain energy difference between the two transition states, the overall ΔΔ*G*_stereo_^‡^ remains close to zero because TS1_*SS→RR*_ benefits from a more favorable entropic contribution. The different positioning of the growing chain within the catalytic pocket results in a less constrained transition-state structure, which is consistent with the more favorable entropy calculated for TS1_*SS→RR*_. Thus, the unfavorable strain-energy contribution is partially compensated by the entropic term, resulting in only a small overall free-energy difference between the two pathways (Figure S32).

Overall, these results indicate that stereocontrol originates from a cooperative interplay between chain-end and catalyst-controlled effects. In both propagation pathways, the chirality of the growing chain dictates the preferred monomer enantioface by minimizing chain-monomer steric interactions. Simultaneously, the ligand framework influences chain-end positioning and monomer distortion. Again, this mechanistic picture suggests analogies with the stereocontrol observed in propylene polymerization catalysts. In the latter, the chiral catalyst dictates a chiral conformation of the growing chain, which, in turn, selects the enantioface of the incoming monomer.^9^ However, for olefin polymerization catalysis, stereoselectivity is generally focused on ESC with secondary contributions from CEC.^11,77^ Instead, the present system operates through a mixture of ESC and CEC, with catalyst chirality and chain positioning both having profound impacts on transition state energy.

Another key observation in our system is that Δ*G*_*RR*→*RR*_^‡^ is approximately 1 kcal·mol^–1^ lower in energy than the other three TSs. To rationalize the stability of the Δ-*RR* iso-insertion and quantify the steric environment experienced by the propagating chain, buried volume (%*V*_Bur_) analysis of the catalyst topology was performed.^78–80^ The analysis focused on the octants occupied by the monomer and propagating chain (+*z*, see SI for details and Table S10).^81–84^ The *C*_2_ symmetry of the Δ configuration generates a well-defined steric map in which the NW and SE octants display similar occupancies (70.5% and 66.5%, Figure S33), as do the SW and NE octants (45.1% and 42.9%, Figure S33). For clarity, a quadrant representation of the steric environment is shown in Figure 6, where light gray quadrants correspond to highly hindered areas and white quadrants to less congested ones. Mapping the RDS geometries onto this representation reveals more favorable accommodation of the (*R*,*R*)-chain within the catalyst pocket. Specifically, the (*R*,*R*)-chain occupies the less hindered NE quadrant, whereas the (*S*,*S*)-chain is directed toward the more congested NW quadrant, thereby increasing the activation barrier of the favored TS1_*SS*→*SS*_ (Δ*G*_*RR*→*RR*_^‡^ = 19.4 kcal·mol^–1^ vs Δ*G*_*SS*→*SS*_^‡^ = 20.5 kcal·mol^–1^).

**Figure 6:**
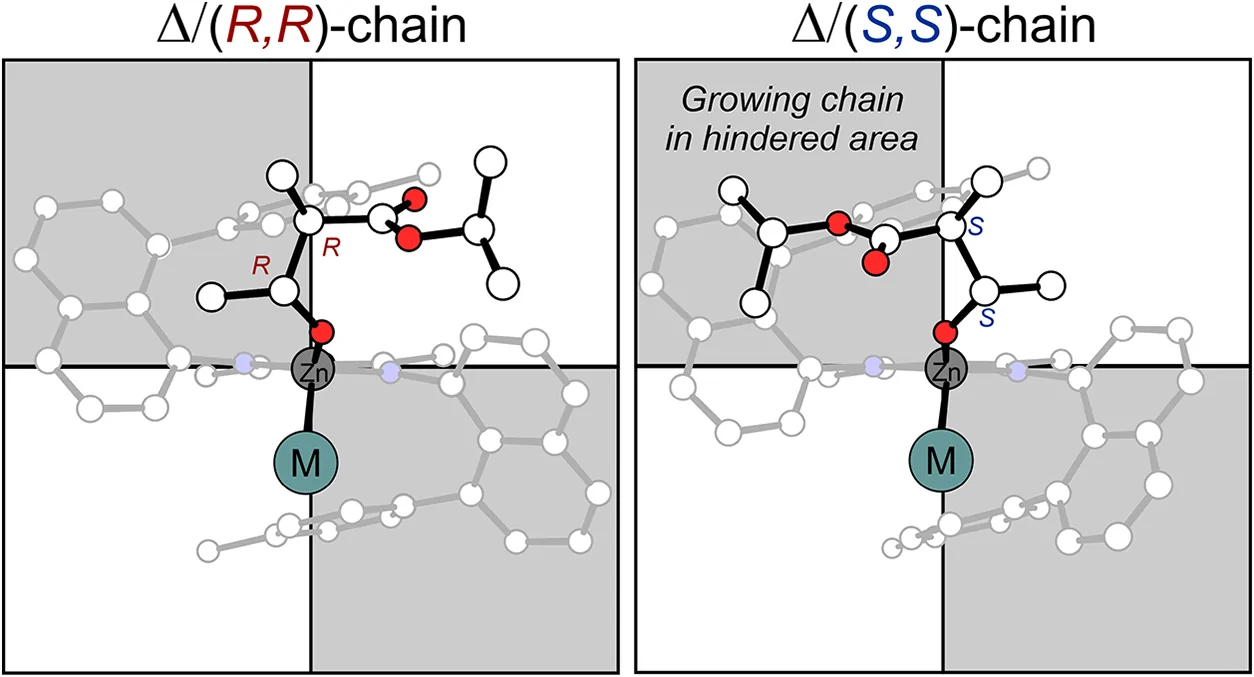
Quadrant representation of the rate determining transition states bearing (*R*,*R*)- or (*S*,*S*)-chain ends. Gray quadrants represent highly hindered areas. M = *trans*-DMPL.

In summary, the computational findings are in excellent agreement with the experimental results (Table 3). Specifically, Δ-*RR* is isoselective for another (*R*,*R*)-DMPL insertion ∼90% of the time (*P*_*RR*_^Δ*RR*^) and Δ-*SS* is essentially unselective with a slight preference for (*S*,*S*)-DMPL insertion (*P*_*SS*,exp_^Δ*SS*^ = 0.50 ± 0.04; *P*_*SS*,theo_^Δ*SS*^ = 0.58). Knowing *P*_*RR*,theo_^Δ*RR*^ and *P*_*SS*,theo_^Δ*SS*^, the value of *P*_rxn,theo_^Δ*RR*^ can be calculated (See SI section 3.2) to be 0.81, close to the experimental value (*P*_rxn,exp_^Δ*RR*^ = 0.830 ± 0.012). Additionally, the DFT calculations allow us to answer the question: do more reactions occur at Δ-*RR* due to it being in higher concentration or faster than Δ-*SS*? At steady state, the rate of Δ-*RR* turning into Δ-*SS* and vice versa must be equal (i.e., *k*_*RR*→*SS*_^Δ^[Δ-*RR*]­[(*S,S*)-*trans*-DMPL] = *k*_*SS*→*RR*_^Δ^[Δ-*SS*]­[(*R,R*)-*trans*-DMPL]). From the MEP, it is known that Δ*G*_*RR*→*SS*_^‡^ and Δ*G*_*SS*→*RR*_^‡^ are both 20.7 kcal·mol^–1^ and thus the reactions have equal rate constants (*k*_*SS*→*RR*_^Δ^ = *k*_*RR*→*SS*_^Δ^). Moreover, since racemic monomer is being polymerized by racemic catalyst, throughout polymerization [(*R,R*)-*trans*-DMPL] is equal to [(*S,S*)-*trans*-DMPL]. As a result, the steady state expression can be simplified to [Δ-*RR*] = [Δ-*SS*], indicating that the populations of Δ-*RR* and Δ-*SS* are equal. This signifies more reactions occur at Δ-*RR* because this species is faster rather than more populous. The equal populations of Δ-*RR* and Δ-*SS* can partially explain the narrow dispersities observed with *rac*-**1**. After the unselective first insertion, there is 25% each of Δ-*RR*, Δ-*SS*, Λ-*RR*, and Λ-*SS*, which happens to be the equilibrium population distribution. Thus, minimal to no time is needed to achieve steady state yielding uniform propagation rates for all catalytic species.

**Table 3:**
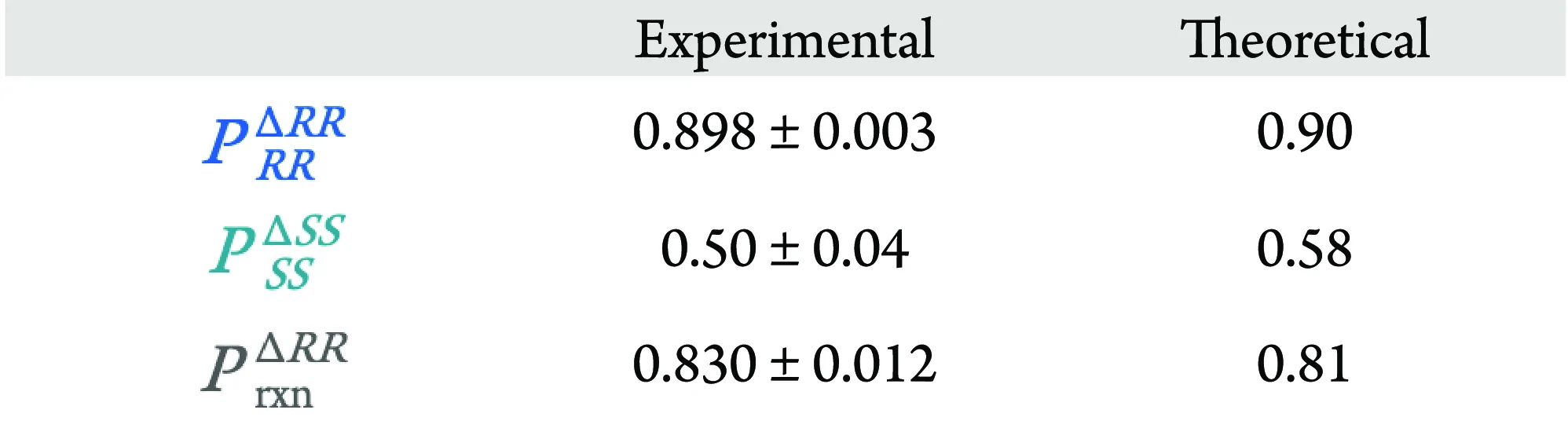
Comparison of Experimental and Computational Insertion Probability Results

### Experimental Determination of Rate Constants

To gain additional support for the site-mediated asymmetric chain-end control mechanism of *rac*-**1** and obtain experimental evidence for the equal populations of Δ-*RR* and Δ-*SS*, 86 %*ee* (*R*,*R*)-DMPL (200 equiv) was polymerized using *rac*-**1**. Notably, the molecular weight distribution of the resulting polymer was broadened and polymodal (Figure S8) contrasting with the monomodal distribution achieved when racemic *trans*-DMPL is polymerized. During *rac-trans*-DMPL polymerization, there are equal concentrations of (*R*,*R*)- and (*S*,*S*)-*trans*-DMPL, so both the Δ and Λ catalyst have equal access to their preferred monomer causing them to both propagate at the same rate. However, at the beginning of polymerization with 86 %*ee* (*R*,*R*)-DMPL, the Δ catalyst is significantly faster due to the higher concentration of its preferred monomer while the Λ catalyst is slower (Figure 7A). This, in turn, causes the %*ee* of the monomer to decrease with polymerization time (Figure 7B). As a result, during the polymerization, the Δ catalyst will slow down, and the Λ catalyst will increase in rate. Furthermore, the polymers produced by both catalysts will have decreasing (*R*,*R*)-block lengths and increasing (*S*,*S*)-blocks.

**Figure 7:**
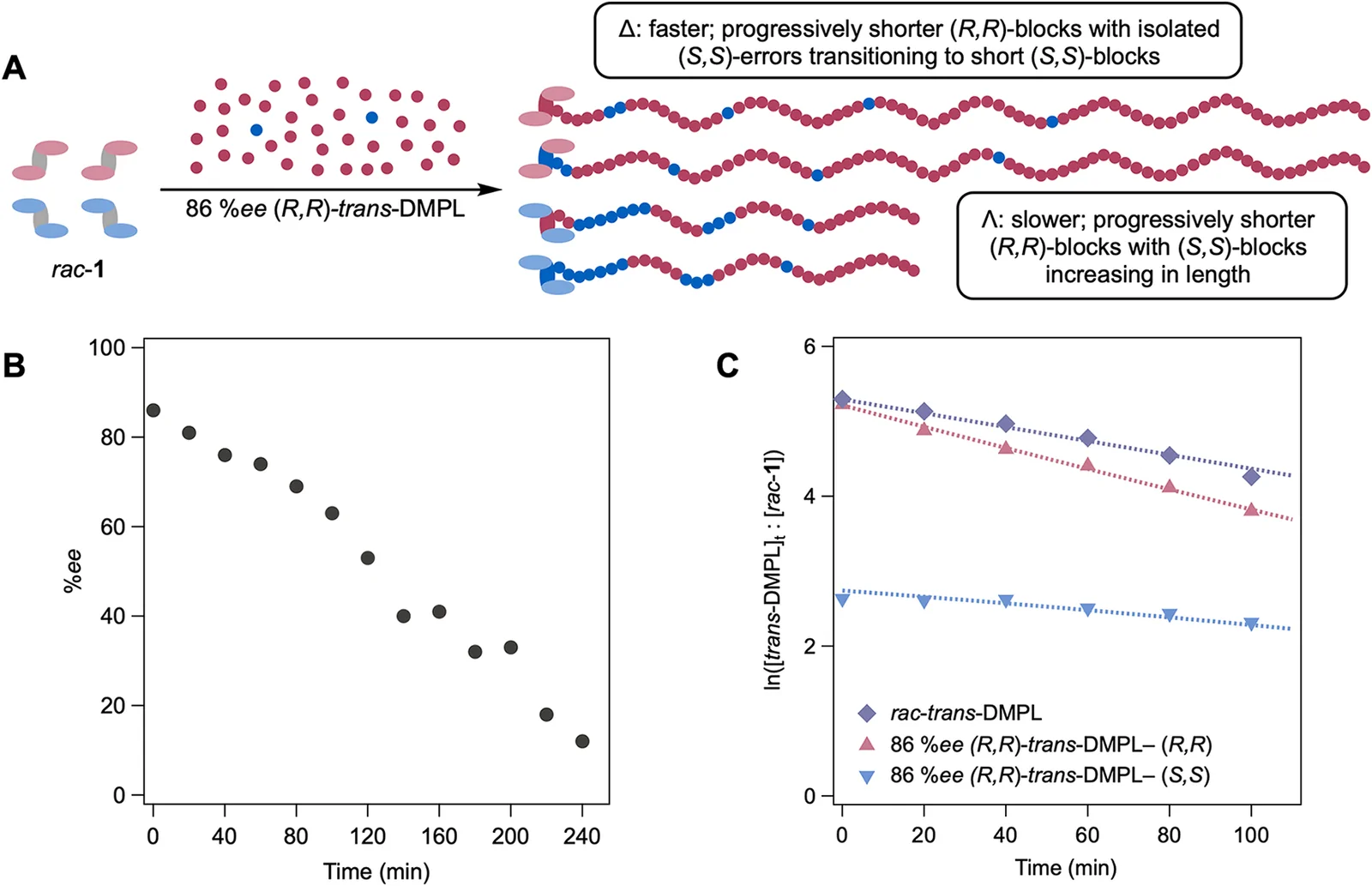
(A) Illustration of the outcomes for polymerization of racemic and enantioenriched (86 %*ee*) *trans*-DMPL by *rac*-**1** (B) %*ee* as a function of time (C) consumption of racemic and enantioenriched (split into *R*,*R* and *S*,*S*) *trans*-DMPL with the fits generated by the system of differential equations.

The decreasing %*ee* of the monomer during polymerization causes the populations of Δ-*RR*, Δ-*SS*, Λ-*RR*, and Λ-*SS* to change, enabling the fitting of enantioenriched and racemic kinetic data with a system of differential equations to abstract the rate constants (Figure 7C; *k*_*RR*→*RR*_^Δ^, *k*_*RR*→*SS*_^Δ^, *k*_*SS*→*SS*_^Δ^, and *k*_*SS*→*RR*_^Δ^). Values for these rate constants were determined by a least-squares fit of the model to the experimental kinetic data. The first 100 min of the polymerization were fit due to deviations from first order behavior later in the polymerization, likely due to changes in viscosity. Uncertainty estimates were determined by Monte Carlo simulations and are expressed as the standard deviation of the mean (for further details, see SI section 3.6 and the annotated Python code).^85,86^ Best-fit values for the rate constants were determined to be *k*_*RR*→*RR*_^Δ^ = 27.7 ± 1.3 mM^–1^·s^–1^, *k*_*RR*→*SS*_^Δ^ = 3.13 ± 0.17 mM^–1^·s^–1^, *k*_*SS*→*RR*_^Δ^ = 3.1 ± 0.4 mM^–1^·s^–1^, and *k*_*SS*→*SS*_^Δ^ = 3.1 ± 0.6 mM^–1^·s^–1^. Using the Eyring equation, values of Δ*G* ^‡^ close to the DFT values are obtained (Scheme 4). The proportion of Δ catalysts with (*R*,*R*)-chain ends was determined to be 50 ± 4% with racemic *trans*-DMPL. Overall, experimental and computational data converge at the same conclusion: at steady state with racemic monomer, the populations of Δ-*RR* and Δ-*SS* are equal.

**Scheme 4:**
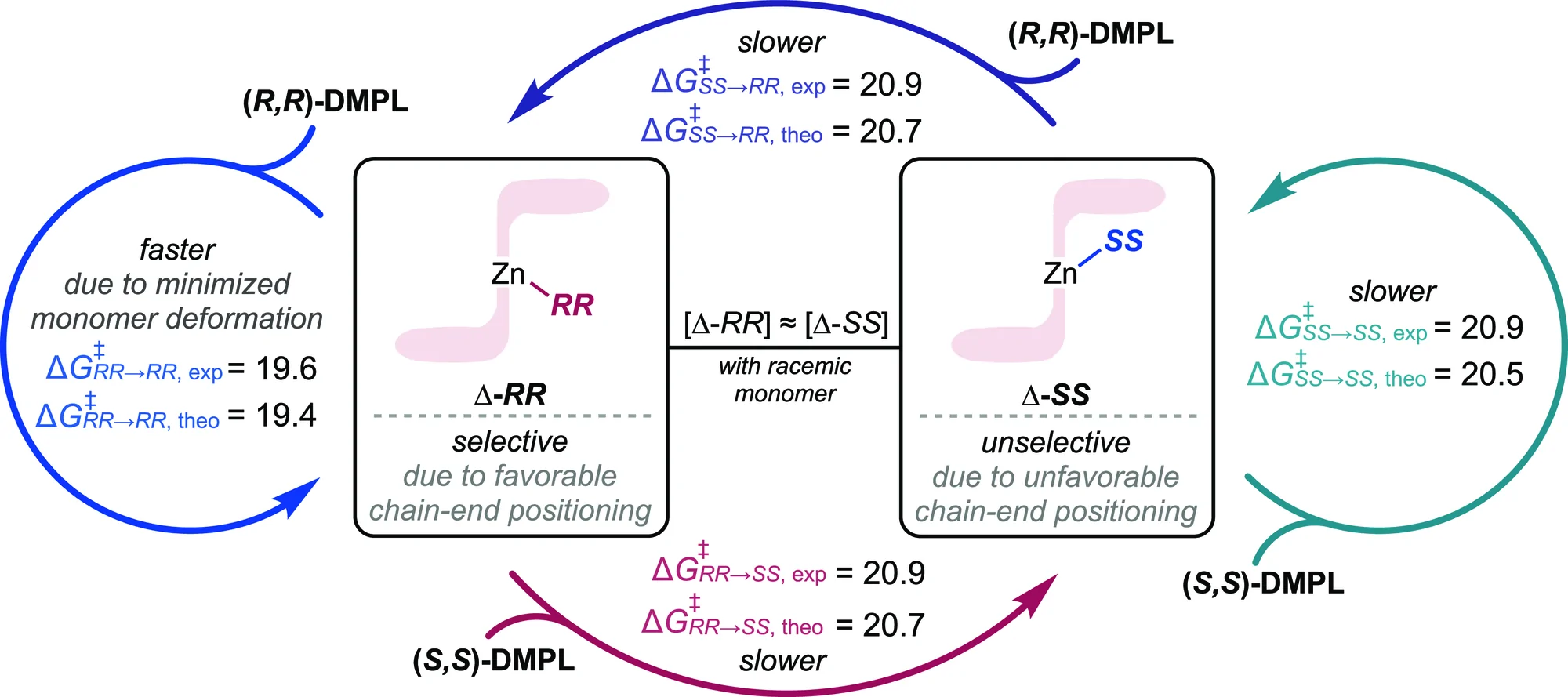
Comprehensive Polymerization Mechanism (Units of All Values are kcal·mol^–1^)

### Unified Mechanistic Summary

Taking all experimental and computational findings together, the full mechanism of polymerization can be described as follows (Scheme 4). The first insertion by the isopropoxide on *rac*-**1** is unselective. Considering only the Δ-catalyst, the presence of a polymer chain on the catalyst creates two diastereomeric resting states: Δ-*RR* and Δ-*SS.* The Δ-*RR* catalyst preferentially inserts a (*R*,*R*)-*trans*-DMPL monomer 90% of the time (*P*_*RR*_^Δ*RR*^) to create an isotactic enchainment. This stereochemical preference is due to an unfavorable monomer-ligand interaction causing significant monomer distortion in the syndiotactic transition state. Conversely, Δ-*SS* is unselective. The syndiotactic transition state for Δ-*SS* also experiences significant monomer distortion, but it is compensated for by favorable entropic contributions in the isotactic transition state. Moreover, experimental and computational evidence shows that *k*_*RR*→*SS*_^Δ^ ≈ *k*_*SS*→*RR*_^Δ^ indicating that at steady state, when polymerizing *rac-trans*-DMPL, the populations of Δ-*RR* and Δ-*SS* are approximately equal. Finally, the Δ-*RR* catalyst is not only more selective but also more active due to more favorable chain positioning in the stereodetermining transition state when inserting (*R*,*R*)-*trans*-DMPL. This stereochemical analysis can be applied to the Λ-catalyst by inverting all monomer and polymer stereogenic centers. In summary, isoselectivity is not dominated by an ESC or CEC mechanism–in fact neither mechanism alone is particularly selective in this polymerization. Rather, all elements of chirality work synergistically to generate the specific cooperative ESC/CEC behavior described here as site-mediated asymmetric chain-end control.

## Conclusions

In conclusion, we report a detailed experimental and computational study of the polymerization of *trans*-DMPL by *rac*-(^4‑Me^BDI*)­ZnO^*i*^Pr (*rac*-**1**). *Rac*-**1** polymerizes up to 5000 equivalents of *trans*-DMPL while maintaining molecular-weight control, narrow dispersity, and high isoselectivity (71–73 *mm*%). Solid- and solution-state studies indicate that *rac*-**1** is monomeric, unlike less bulky (BDI)Zn alkoxides. Using experimental and computational studies, we present a detailed molecular picture of how enantiomorphic-site control and chain-end control cooperate in this catalyst system. We describe this specific mechanistic manifestation of cooperative stereocontrol as site-mediated asymmetric chain-end control. In short, each catalyst enantiomer, Δ and Λ, has a favored and unfavored chain end. When the catalyst has its favored chain end, ∼90% of the time another favored monomer will be added, and the catalyst’s overall rate is faster. Conversely, when the catalyst has its unfavored chain end, it is slower and essentially unselective. Experimental and computational investigations converge with remarkably good agreement on relative rates and selectivities. DFT calculations enabled a molecular-level description of the source of stereocontrol highlighting the interplay between chain positioning, monomer distortion, and ligand interactions. We imagine that this work will inform the design and understanding of other chiral lactone ROP catalysts with stereo and molecular weight control. Future work will focus on modifying catalyst structure to precisely control the ratios of all rate constants and tune the resulting polymer microstructures.

## Supplementary Material







## Notes

Experimental procedures; spectral data, and computational details (PDF)
Annotated Python scripts (ZIP)
Optimized Cartesian coordinates (XYZ)
