# Supplementary material for: Cooperative Enantiomorphic-Site and Chain-End Chirality in Isoselective β‑Lactone Polymerization
Source: J Am Chem Soc. 2026 Oct 22;148(43):46987–99. doi: 10.1021/jacs.6c14172 (PMC13741839; doi:10.1021/jacs.6c14172)
Supplement: Supplementary file 1 [file ja6c14172_si_001.pdf]

Supporting Information for:

Cooperative Enantiomorphic-Site and Chain-End Chirality in Isoselective β-Lactone Polymerization

Morgan S. Young,[‡,a] Yolanda Rusconi,[‡,b,c] Ethan B. Flanagan,[a] Samantha N. MacMillan,[a] Claudio De Rosa,[c] Anne M. LaPointe,[a] Giovanni Talarico,[b,c]* and Geoffrey W. Coates[a]*

[a] Department of Chemistry and Chemical Biology, Baker Laboratory, Cornell University, Ithaca, New York 14853-1301, United States

[b] Scuola Superiore Meridionale, Largo San Marcellino, 80138 Naples, Italy

[c] Department of Chemical Sciences, University of Naples Federico II, Via Cintia, 80126 Naples, Italy

Table of Contents

1 Materials and methods 1

1.1 General considerations 1

1.2 Materials 1

1.3 Characterization Methods 2

2 Synthetic Procedures 3

2.1 Synthesis of *rac*-($^{4\text{-Me}}$BDI*)Zn(N(SiMe$_3$)$_2$) 3

2.2 General Polymerization Procedure 4

2.3 Kinetics Procedure for Rate Order Determination 5

2.4 Procedure for Racemic and Enantioenriched Kinetics 5

3 Experimental Details and Polymer Characterization 6

3.1 Example Tacticity Calculation 6

3.2 Full Explanation of ^{13}C{^{1}H} NMR Model 7

3.3 Polymer Tacticity Information for Table 1, entries 1–8 10

3.4 SEC Chromatograms for Samples Made with Chain Transfer Agent (CTA) 10

3.5 Racemic and Enantioenriched Kinetics 11

3.6 Kinetic Model for Rate Constant Determination 15

4 NMR Spectra 19

4.1 BDIZnO^{i}Pr Complexes 19

4.2 Selected *trans*-PHMB ^{13}C{^{1}H} NMR Spectra 20

5 Computational Details 26

5.1 General Information 26

5.2 Benchmark Calculations with Alternative Functionals 27

5.3 Noncovalent Interaction (NCI) Analysis 27

5.4 Comparison of Mononuclear and Dinuclear Structures of *rac*-1 28

5.5 Elements of Chirality and Proposed Polymerization Mechanism 29
5.6 Minimum Energy Pathway of Initiation 30
5.7 Chain Conformation Analysis 32
5.8 Activation Strain Model (ASM) Analysis 34
5.9 TS1 Structures for INT3$_{SS}$-Δ (Δ-*SS*) 34
5.10 Buried Volume (%V_{Bur}) Analysis 35
6 X-Ray Crystallographic Data 36
6.1 *rac*-($^{\mathrm{4\text{-}Me}}$BDI*)ZnO^{i}Pr (*rac*-1) 36
6.2 *rac*-($^{\mathrm{4\text{-}Me}}$BDI*)Zn(N(SiMe$_3$)$_2$ 46
7 References 57

1 Materials and Methods

1.1 General Considerations

Unless otherwise indicated, all reactions and manipulations of air- and/or water-sensitive compounds were carried out under a dry nitrogen atmosphere using an MBraun glovebox or standard Schlenk techniques. Toluene and THF for polymerizations and air-free reactions were purchased from Fisher, sparged with ultrahigh purity (UHP) grade nitrogen, passed through two columns containing reduced copper (Q-5) and alumina and dispensed into an oven-dried Straus flask. The solvent was brought into the glovebox and stored over activated 3 Å sieves for 24 h before use. Chloroform-*d* ($CDCl_3$) and benzene-d_6 (C_6D_6) were purchased from Cambridge Isotope Laboratories. $CDCl_3$ was used without further purification. C_6D_6 was sparged with nitrogen for 15 min before being stored in a glovebox over alumina beads for 24 h before use. Otherwise, solvents were purchased from commercial sources and used without further purification.

1.2 Materials

CaH_2 was purchased from Acros Organics (10 - 100 mm pieces) and was ground into a fine powder using a mortar and pestle immediately before use. Anhydrous isopropanol was purchased from Millipore Sigma and was dried over CaH_2 for 3 d under an N_2 atmosphere, vacuum transferred and degassed with three freeze-pump-thaw cycles before use. $Zn(N(SiMe_3)_2)_2$ was synthesized as previously reported.[1] $^{4\text{-Me}}$BDI*H and ($^{4\text{-Me}}$BDI*)ZnO^{i}Pr (*rac*-**1**) were synthesized as previously reported.[2] *rac*-*trans*-3,4-Dimethylpropiolactone (*trans*-DMPL) was synthesized according to a modified literature procedure.[3] The *trans*-DMPL was stirred over freshly ground CaH_2 and carbon black for 3 d, vacuum transferred, freeze-pump-thawed x 3, brought into a glovebox and stored over alumina beads (BASF F200) at -25 °C for 24 h before use.

1.3 Characterization Methods

Nuclear magnetic resonance (NMR) spectroscopy: All NMR spectra were acquired at 25 °C. ^{1}H and ^{13}C{^{1}H} NMR spectra were recorded on a Bruker AVANCE III HD spectrometer with a broadband Prodigy cryoprobe at 25 °C (operating at 500 MHz for ^{1}H). For ^{1}H and ^{13}C{^{1}H} spectra, chemical shift data is reported in units of δ (ppm) relative to tetramethylsilane (TMS). ^{1}H NMR spectra were referenced to the residual non-deuterated solvent (7.26 ppm for $CDCl_3$ and 7.16 ppm for C_6D_6) and ^{13}C{^{1}H} NMR spectra were referenced to the deuterated solvent itself (77.16 ppm for $CDCl_3$ and 128.06 ppm for C_6D_6). NMR spectra were processed in MestReNova 14.3.2. NMR spectroscopic data are reported as follows: chemical shift, multiplicity (s = singlet, d = doublet, t = triplet, q = quartet, hept = heptet, m = multiplet, b = broad), coupling constants (Hz) and integration.

Size exclusion chromatography (SEC): SEC analyses were carried out using an Agilent 1260 Infinity SEC System equipped with an Agilent 1260 Infinity autosampler and a refractive index detector. The Agilent SEC system was equipped with a guard column (4.6 x 50 mm) and two Agilent PolyPore MiniMix columns (5-micron, 4.6 x 250 mm ID) which were eluted with THF at 30 °C at 0.3 mL/min and calibrated using monodisperse polystyrene standards. Prior to analysis, polymer samples (1 mg/mL THF) were gently heated to dissolve any residual polymer particles before being passed through a 0.45 µm PTFE syringe filter.

Chiral gas chromatography (Chiral GC): GC analysis to determine percent enantiomeric excess was done using a Hewlett-Packard 6890 gas chromatograph equipped with a Supelco β-Dex 225 column (L × I.D. 30 m × 0.25 mm, df 0.25 µm) and a flame-ionization detector. Method for *trans*-DMPL: The injection port is set to 150 °C. After injection, the oven temperature increases from 50 °C to 200 °C at 8 °C/min. (*R,R*)-*trans*-DMPL is eluted at ~14.9 min, and (*S,S*)-*trans*-DMPL is eluted at ~15.2 min. The 86 %*ee* (*R,R*)-*trans*-DMPL was prepared as previously reported.[2]

X-ray Diffraction (XRD): Low-temperature X-ray diffraction data for Rmsy2 and Rmsy3 were collected on a Rigaku XtaLAB Synergy diffractometer coupled to a Rigaku Hypix detector with Cu Kα radiation (λ = 1.54184 Å), from a PhotonJet micro-focus X-ray source at 100 K. The diffraction images were processed and scaled using the CrysAlisPro software.[4] The structures were solved through intrinsic phasing using SHELXT[5] and refined against F^2 on all data by full-matrix least-squares with SHELXL[6] following established refinement strategies.[7] All non-hydrogen atoms were refined anisotropically. All hydrogen atoms bound to carbon were included in the model at geometrically calculated positions and refined using a riding model. The isotropic displacement parameters of all hydrogen atoms were fixed to 1.2 times the U_{eq} value of the atoms they are linked to (1.5 times for methyl groups). Details of the data quality and a summary of the residual values of the refinements are listed in **Table S11–Table S20Table S19**.

2 Synthetic Procedures

2.1 Synthesis of *rac*-($^{4\text{-Me}}$BDI*)Zn(N(SiMe$_3$)$_2$)

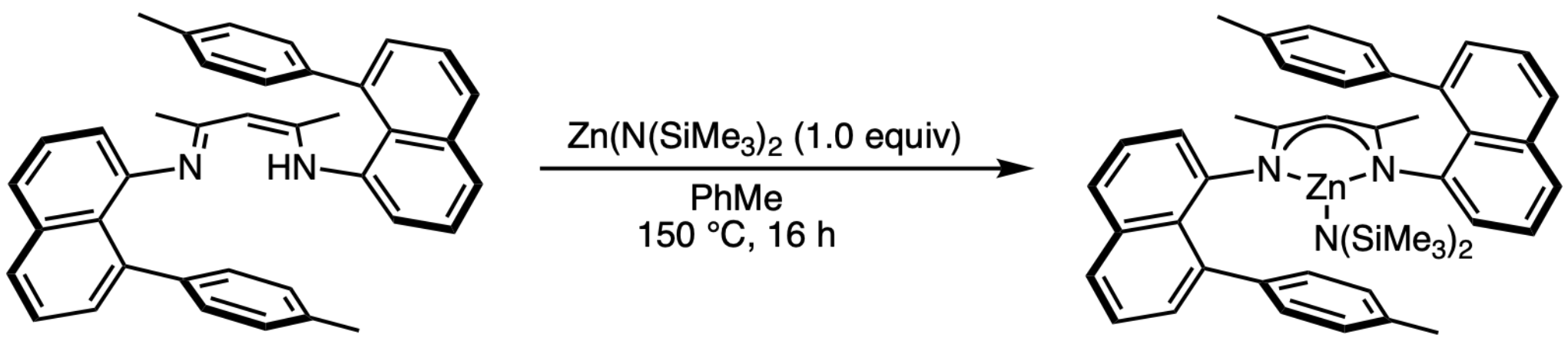

***rac*-($^{4\text{-Me}}$BDI*)Zn(N(SiMe$_3$)$_2$):** In a glovebox, $^{4\text{-Me}}$BDI*H[2] (0.200 g, 0.377 mmol, 1.00 equiv), Zn(N(SiMe$_3$)$_2$)$_2$ (152 μL, 0.377 mmol, 1.00 equiv, ρ = 0.957 g/mL) and PhMe (8 drops) were added to a 4 mL vial with stir bar and heated to 150 °C for 16 h. An aliquot was taken for ^{1}H NMR spectroscopic analysis to confirm full conversion of the ligand. The reaction mixture was then heated to 150 °C *in vacuo* for 16 h to remove the volatiles. The title compound was isolated as a golden yellow solid with 0.09 equiv HMDSH remaining and stored at -25 °C (0.275 g, 95%). X-ray quality crystals were obtained from a solution of hexanes and toluene (ca. 10:1) at -25 °C. **^{1}H NMR** (500 MHz, C$_6$D$_6$) δ 7.67 (dd, *J* = 8.1, 1.6 Hz, 2H), 7.61 (dd, *J* = 8.0, 1.5 Hz, 2H), 7.59 (bs, 2H) 7.43 (t, *J* = 7.7 Hz, 2H), 7.38 (dd, *J* = 7.3, 1.5 Hz, 2H), 7.24 (dd, *J* = 7.0, 1.6 Hz, 2H), 7.19 (dd, *J* = 8.1, 7.0 Hz, 2H), 7.12 (bs, 2H), 6.95 (bs, 2H), 6.82 (bs, 2H), 3.33 (s, 1H), 2.09 (s, 6H), 1.37 (s, 6H), -0.01 (s, 18H). **^{13}C{^{1}H} NMR** (126 MHz, C$_6$D$_6$) δ 167.68, 146.15, 141.83, 139.49, 136.57, 135.18, 131.99, 129.44, 127.64, 126.96, 125.64, 125.31, 125.20, 98.92, 25.16, 21.20, 6.08.

2.2 General Polymerization Procedure

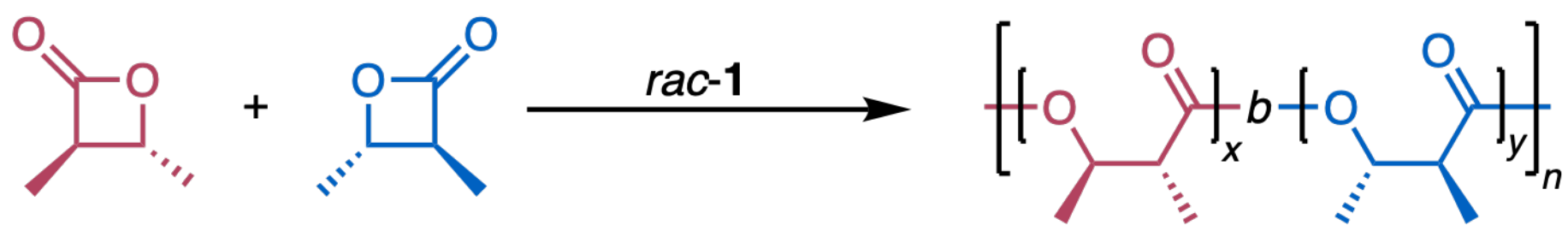

In a glovebox, *rac-trans*-DMPL was added to a solution of *rac*-**1** in toluene inside of a vial equipped with a stir bar. For polymerization using chain transfer agent (CTA), ^{i}PrOH as a solution in PhMe was then added. The vial was capped and stirred at 25 °C. Once polymerization time concluded, the vial was brought out of the glovebox, quenched with benchtop CH_2Cl_2, and homogenized with a vortex and gentle heat when necessary. An aliquot was taken for ^{1}H NMR spectroscopic analysis to determine conversion and for SEC analysis to determine crude molar mass data.

Representative procedure for Table 1, entry 1: In a glovebox, *rac-trans*-DMPL (100 µL, 1.00 mmol, 200 equiv) was added to a solution of *rac*-**1** (5.0 µmol, 150 µL, 0.033 M in toluene) in a 2 mL vial equipped with a stir bar. The vial was capped and stirred at the 25 °C for 18 h. Then, the vial was brought out of the glovebox, quenched with benchtop CH_2Cl_2, and homogenized before analysis.

<u>Precipitation procedure:</u>

Polymers were precipitated from a concentrated (~100 mg/mL) solution in CH_2Cl_2 with cold MeOH (7 mL, -20 °C) in vials or culture tubes. The polymer suspensions were placed at -20 °C for 15 min before being centrifuged. The supernatant was gently pipetted off and the polymer dried *in vacuo*. The polymer was then redissolved in a minimal amount of CH_2Cl_2 and precipitated with cold (-20 °C) hexanes. The polymer suspensions were placed at -20 °C for 15 min before being centrifuged. The supernatant was gently pipetted off and the polymer dried *in vacuo* for 16 h before being subjected to $^{13}C\{^{1}H\}$ NMR spectroscopic and SEC analysis.

2.3 Kinetics Procedure for Rate Order Determination

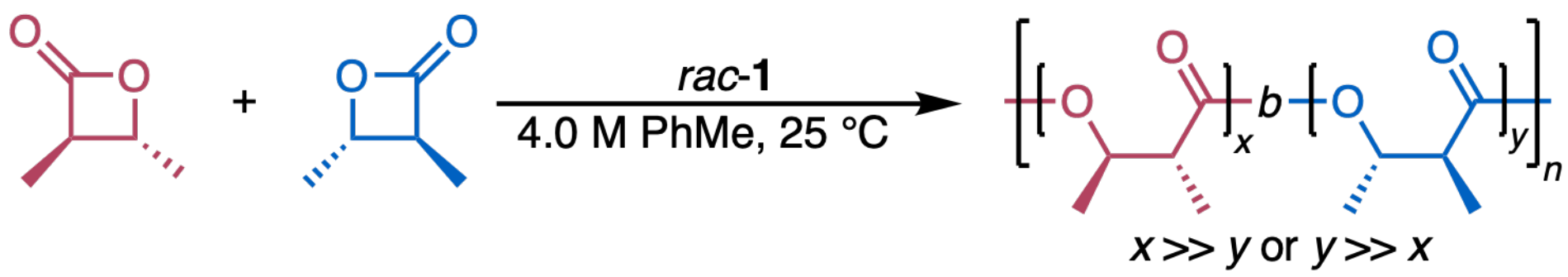

In a glovebox at 25 °C, *rac-trans*-DMPL was added to solutions of *rac*-**1** in toluene (2.0 mmol *trans*-DMPL, [*trans*-DMPL]$_0$= 4.0 M, total volume = 0.50 mL). Different Zn concentrations were achieved by diluting a single catalyst stock solution to mitigate error between polymerizations. The solutions were mixed, distributed among 2 mL vials and capped. At the designated time, the vials were quenched in the glovebox with 4-*tert*-butylbenzoic acid in $CDCl_3$. The time points were individually subjected to ^{1}H NMR spectroscopic analysis to determine conversion.

2.4 Procedure for Racemic and Enantioenriched Kinetics

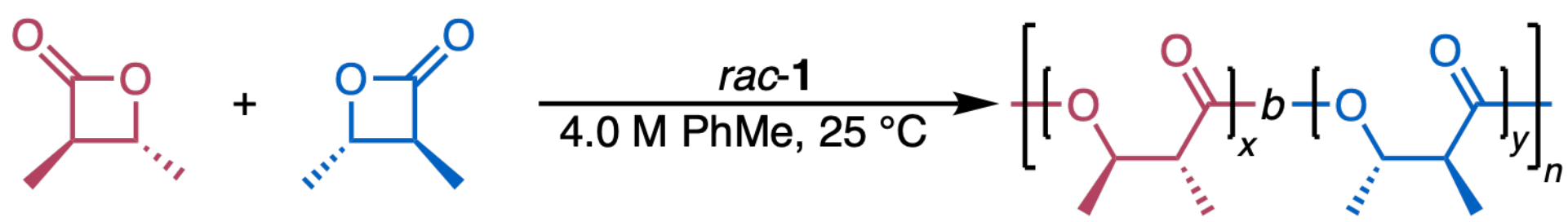

racemic or
86 %*ee* (*R,R*)-*trans*-DMPL

In a glovebox, either racemic (0.26 mL, 0.26 mmol, 200 equiv) or 86 %*ee* (*R,R*)-*trans*-DMPL (containing 0.05 equiv THF, 0.26 mL, 0.26 mmol, 200 equiv) was added to a solution of *rac*-**1** (0.013 mmol, 1.0 equiv) and cyclododecane (ca. 10 mg, GC standard) in toluene (0.38 mL, [DMPL]$_0$ = 4.0 M in toluene). THF (10 µL) was added to the racemic polymerization. The polymerizations were mixed, distributed among 2 mL vials and capped. At the designated time, the vials were brought outside of the glovebox and quenched with benchtop $CDCl_3$. The time points were individually subjected to ^{1}H NMR spectroscopic analysis to determine conversion. An aliquot of each time point was dried *in vacuo* and subjected to SEC analysis. For the samples made with 86 %*ee* (*R,R*)-*trans*-DMPL, the timepoints were diluted with CH_2Cl_2 such that the concentration of residual *trans*-DMPL was ca. 2 mg/mL. A portion of this solution was taken for chiral GC analysis to determine %*ee*.

3 Experimental Details and Polymer Characterization

3.1 Example Tacticity Calculation

mm%s were calculated as previously reported.[2]

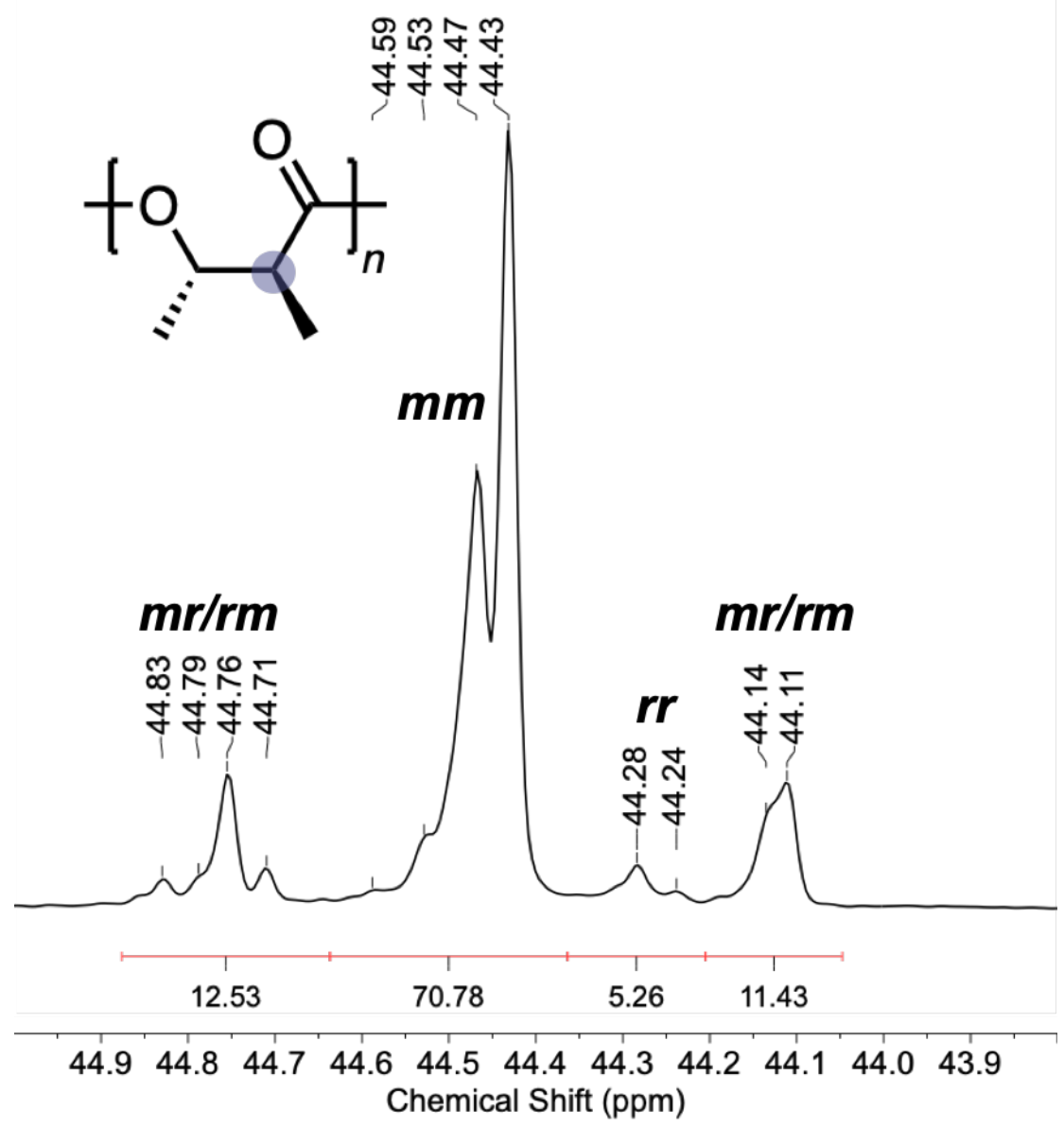

Figure S1: Portion of the $^{13}C\{^1H\}$ NMR (126 MHz, $CDCl_3$) spectrum of isoenriched *trans*-PHMB from Table 1, entry 1.

Equation S1: Determination of *mm%* from a $^{13}C\{^1H\}$ NMR spectrum.

$$mm\% = \frac{(mm)}{(mm) + (rr) + (mr) + (rm)} * 100$$

Using **Equation S1**,

$$mm\% = \frac{(70.78)}{(70.78) + (12.53) + (11.43) + (5.26)} * 100 = 71\%$$

Equation S2: Bernoulli triad test for chain-end control. If B ≅ 1, then system operates by a chain-end control mechanism of stereocontrol.[8]

$$B = \frac{4(mm)(rr)}{[(rm) + (mr)]^2}$$

For the sample made by *rac*-**1** in Figure S1 showed in the text,

$$B = \frac{4(70.78)(5.26)}{[(12.53) + (11.43)]^2} = 2.6$$

3.2 Full Explanation of $^{13}C\{^1H\}$ NMR Model

To create the following NMR model the following assumptions/restrictions were made:

- *Rac*-*trans*-DMPL is being polymerized
- Stereoselectivity is attributed to the stereochemistry of the catalyst and the chain end, and that there is no influence from the penultimate repeat unit or any repeat units further back in the polymer chain.
- The length of the polymer chain has no effect on the rate of polymerization
- Initiation is unselective and much faster than polymerization and thus it can be ignored
- Termination events and polymer exchange between catalysts is negligible

To describe the stereoselectivity of *rac*-**1,** we created a model defining probabilities of *trans*-DMPL (*R,R* or *S,S*) insertions as a function of Δ catalyst resting states. The following analysis can be applied to Λ catalyst by inverting all stereochemical centers on the incoming monomer and chain end. The Δ catalyst can be resting in two diastereomeric states: either with an (*R,R*)-PHMB chain end (Δ-*RR*) or (*S,S*)-PHMB chain end (Δ-*SS*) (**Scheme S1** top and bottom, respectively). First considering the Δ-*RR* case, Δ-*RR* can insert an (*R,R*) or (*S,S*)-*trans*-DMPL. These insertions have a probability of $P_{RR}^{\Delta RR}$ and $P_{SS}^{\Delta RR}$, respectively, and their sum is equal to unity. The subscript of these probability terms denotes the stereochemistry of the incoming monomer, and the superscript indicates the resting state of the starting catalyst. Analogously, $P_{RR}^{\Delta SS}$ and $P_{SS}^{\Delta SS}$ can be applied to Δ-*SS*. These four probabilities do not factor in population or rate differences between the two catalyst resting states, Δ-*RR* and Δ-*SS.* To account for this, we introduced the correction factors $P_{rxn}^{\Delta RR}$ and $P_{rxn}^{\Delta SS}$ which are the probability of any Δ reaction occurring at Δ-*RR* and Δ-*SS*, respectively, and the sum of these two terms is unity.

The insertions described by $P_{RR}^{\Delta RR}$ and $P_{SS}^{\Delta SS}$ lead to an isotactic diad (*m*) while $P_{SS}^{\Delta RR}$, and $P_{RR}^{\Delta SS}$ lead to syndiotactic diad (*r*). However, the $^{13}C\{^1H\}$ NMR spectrum of *trans*-PHMB allows us to measure triads. Thus, to correlate our model to experimental data, the model was expanded to the triad level as shown in **Scheme S1**. Each insertion is independent of the last, thus the resulting triad probability is simply the product of each probability leading to this result. Moreover, the total probability is the sum of the individual probabilities of each triad resulting from Δ-*RR* and Δ-*SS* (**Table S2**).

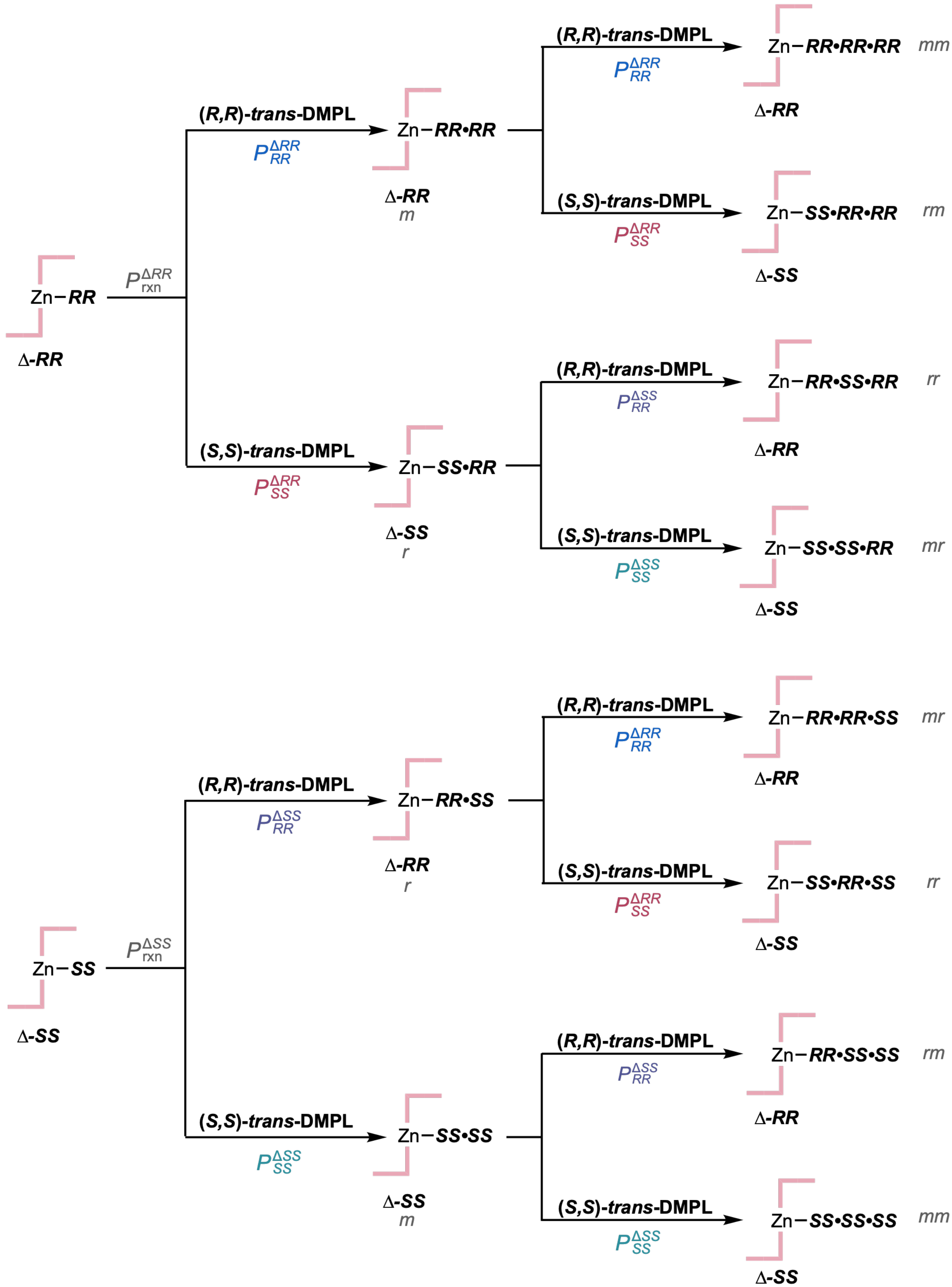

Scheme S1: Pathways leading to all four triads starting from Δ-*RR* (top) and Δ-*SS* (bottom).

Table S1: Probability expressions of the triads as a function of starting species. The total probability is the sum of the Δ-*RR* and Δ-*SS* pathways.

Triad	Probability Expression	
	Δ-*RR* pathway	Δ-*SS* pathway
mm	$P_{rxn}^{\Delta RR} \cdot (P_{RR}^{\Delta RR})^2$	$P_{rxn}^{\Delta SS} \cdot (P_{SS}^{\Delta SS})^2$
mr	$P_{rxn}^{\Delta RR} \cdot P_{SS}^{\Delta RR} \cdot P_{SS}^{\Delta SS}$	$P_{rxn}^{\Delta SS} \cdot P_{RR}^{\Delta SS} \cdot P_{RR}^{\Delta RR}$
rm	$P_{rxn}^{\Delta RR} \cdot P_{RR}^{\Delta RR} \cdot P_{SS}^{\Delta RR}$	$P_{rxn}^{\Delta SS} \cdot P_{SS}^{\Delta SS} \cdot P_{RR}^{\Delta SS}$
rr	$P_{rxn}^{\Delta RR} \cdot P_{SS}^{\Delta RR} \cdot P_{RR}^{\Delta SS}$	$P_{rxn}^{\Delta SS} \cdot P_{RR}^{\Delta SS} \cdot P_{SS}^{\Delta RR}$

If $P_{RR}^{\Delta RR} = x$, $P_{SS}^{\Delta RR} = 1 - x$, $P_{SS}^{\Delta SS} = y$, $P_{RR}^{\Delta SS} = 1 - y$, $P_{rxn}^{\Delta RR} = z$ and $P_{rxn}^{\Delta SS} = 1 - z$, then the probabilities of the triads simplify to:

Table S2: Simplified triad probability expressions.

Triad	Probability
mm	$zx^2 + y^2(1 - z)$
mr	$zy(1 - x) + x(1 - z)(1 - y)$
rm	$zx(1 - x) + y(1 - z)(1 - y)$
rr	$y(1 - x)(1 - y) + (1 - z)(1 - y)(1 - x)$

Moreover, at steady state:

$$P_{rxn}^{\Delta RR} \cdot P_{SS}^{\Delta RR} = P_{rxn}^{\Delta SS} \cdot P_{RR}^{\Delta SS}$$

Which can be rewritten as:

$$z(1 - x) = (1 - z)(1 - y)$$

By solving for z, we obtain:

$$z = \frac{y - 1}{x + y - 2}$$

Since z can be calculated from x and y, this leaves two variables to be solved for. Using a Python script (see additional supplementary zip file), experimental *mm, mr, rm* and *rr* measurements were fit to the equations in **Table S2** by modulating x and y. The error on x and y was minimized using the least squares method. The results of this analysis can be found in **Table S3**.

3.3 Polymer Tacticity Information for Table 1, entries 1–8

Table S3: $^{13}C\{^1H\}$ NMR integrations, Bernoulli CEC test, and calculated $P_{RR}^{\Delta RR}$, $P_{SS}^{\Delta SS}$ and $P_{rxn}^{\Delta RR}$ values for the polymers in Table 1, entries 1-8.

	$^{13}C\{^1H\}$ NMR mol%s[a]							
Entry	[*mm*]	[*mr*]/[*rm*]		[*rr*]	B^b	$P_{RR}^{\Delta RR}(x)$	$P_{SS}^{\Delta SS}(y)$	$P_{rxn}^{\Delta RR}(z)$
1	70.78	12.53	11.43	5.26	2.6	0.8961	0.4937	0.8297
2	71.09	12.15	11.92	4.84	2.4	0.8972	0.5292	0.8208
3	70.64	12.40	11.96	5.00	2.4	0.8952	0.5227	0.8200
4	71.38	12.06	11.76	4.80	2.4	0.8985	0.5276	0.8231
5	70.51	12.17	12.31	5.01	2.4	0.8946	0.5245	0.8186
6	72.56	11.69	10.28	5.48	3.3	0.9038	0.4306	0.8554
7	71.90	11.91	11.34	4.85	2.6	0.9010	0.5103	0.8318
8	71.30	11.45	11.78	5.47	2.9	0.8985	0.4612	0.8414
Mean						**0.898**	**0.50**	**0.830**
Std Dev						**0.003**	**0.04**	**0.012**

[a]Determined by $^{13}C\{^1H\}$ NMR spectroscopic analysis. [b] Calculated using Equation S2.

3.4 SEC Chromatograms for Samples Made with Chain Transfer Agent (CTA)

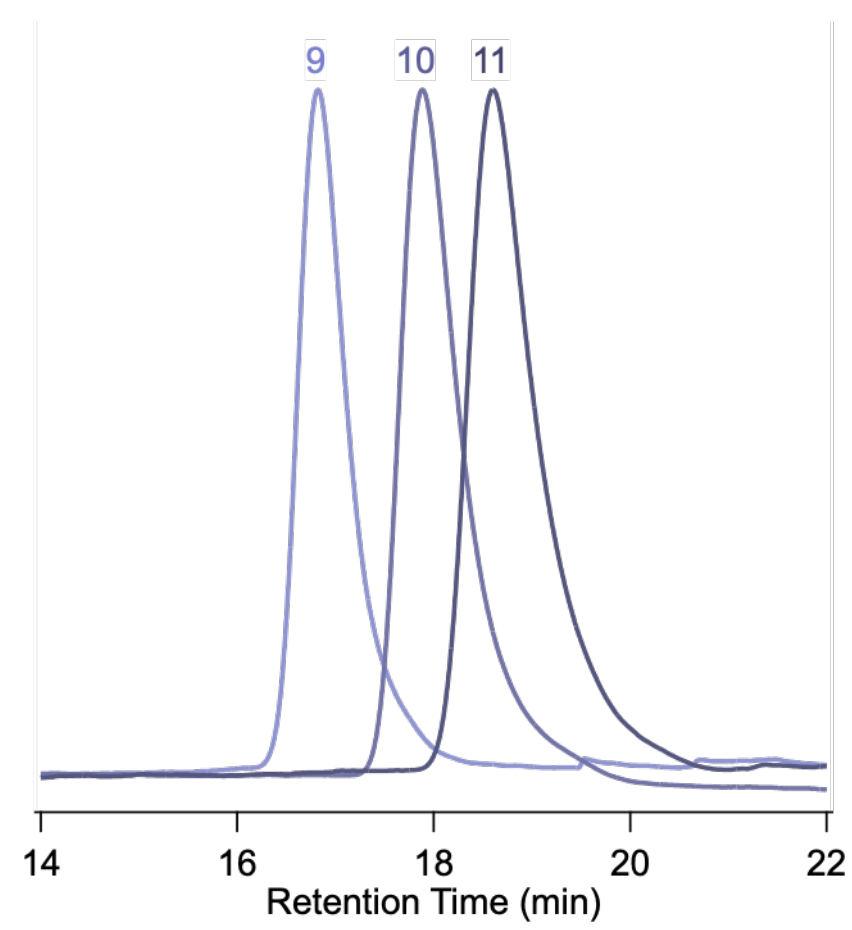

Figure S2: SEC chromatograms of isoenriched *trans*-PHMB samples made using CTA in Table 1. Numbers above curves correlate to entry number.

3.5 Racemic and Enantioenriched Kinetics

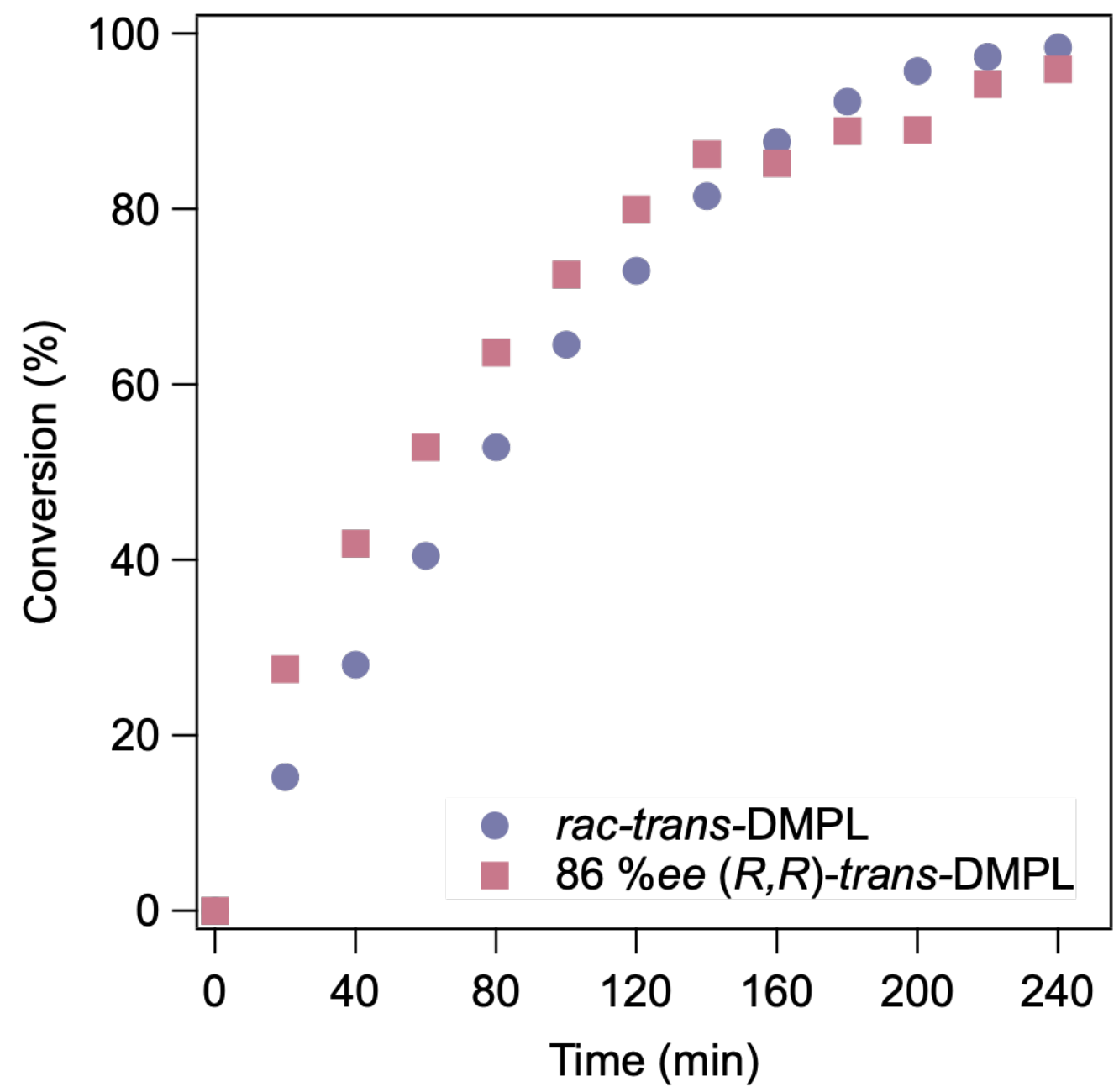

Figure S3: Conversion vs. time for the polymerization of racemic or 86 %*ee* (*R,R*)-*trans*-DMPL by *rac*-**1** ([*trans*-DMPL]$_0$ = 4.0 M toluene, [*trans*-DMPL]$_0$:[*rac*-**1**]$_0$ = 200:1, T_{rxn} = 25 °C).

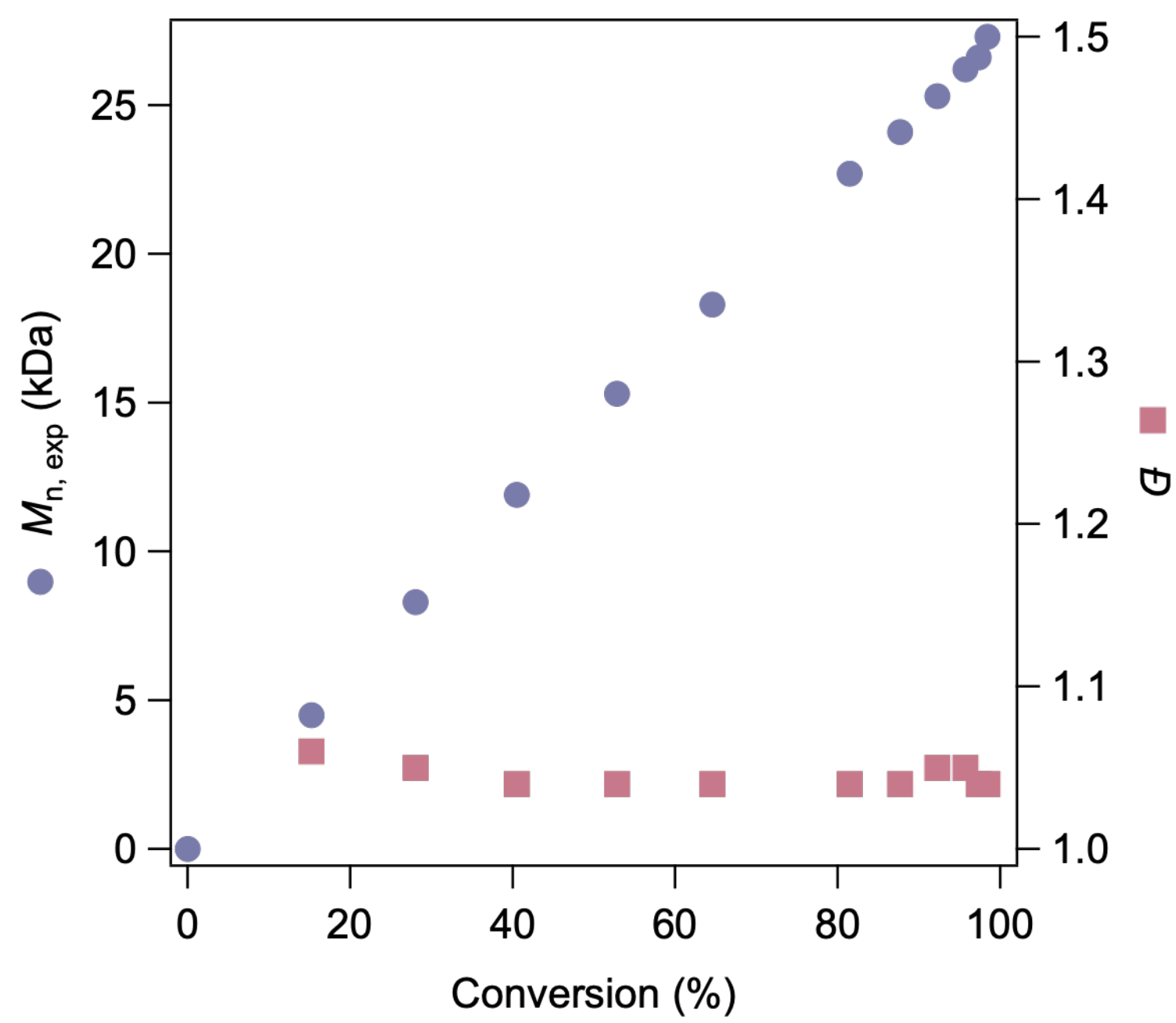

Figure S4: M_n and *Đ* vs. conversion for the polymerization of *rac*-*trans*-DMPL by *rac*-**1** ([*trans*-DMPL]$_0$ = 4.0 M toluene, [*trans*-DMPL]$_0$:[*rac*-**1**]$_0$ = 200:1, T_{rxn} = 25 °C).

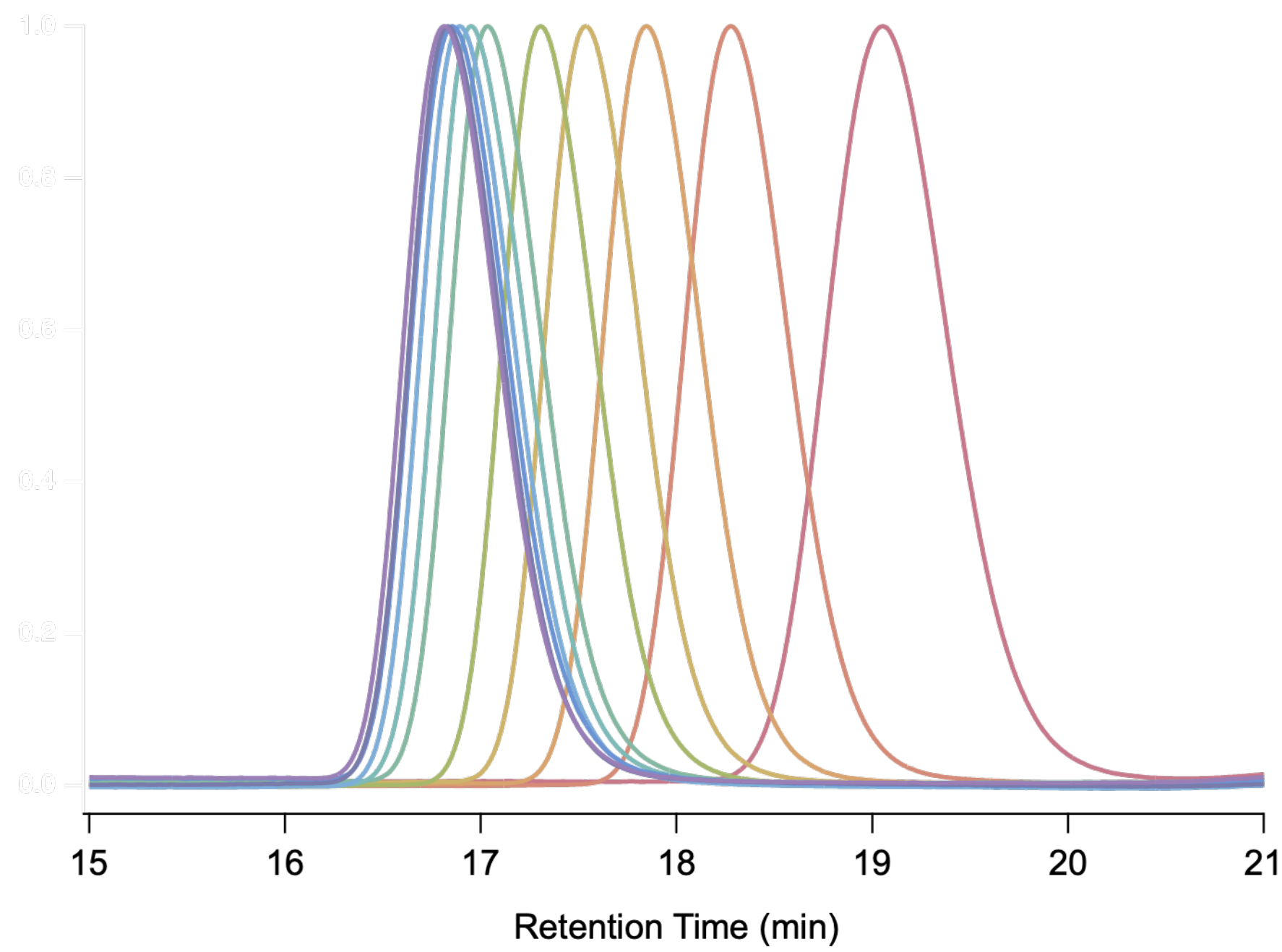

Figure S5: SEC chromatograms of the aliquots from the polymerization of *rac*-*trans*-DMPL by *rac*-**1** ([*trans*-DMPL]$_0$ = 4.0 M toluene, [*trans*-DMPL]$_0$:[*rac*-**1**]$_0$ = 200:1, T_{rxn} = 25 °C).

Table S4: Conversion and molecular weight data for *rac*-*trans*-DMPL kinetics.

Time (min)	Conversion[a]	$M_{n, theo}$ (kDa)	$M_{n, exp}$ (kDa)	$Đ$
20	15	3.1	4.5	1.06
40	28	5.6	8.3	1.05
60	40	8.1	11.9	1.04
80	53	10.6	15.3	1.04
100	65	12.9	18.3	1.04
120	73	14.6	–[b]	–[b]
140	81	16.3	22.7	1.04
160	87	17.6	24.1	1.04
180	92	18.5	25.3	1.05
200	96	19.2	26.2	1.05
220	97	19.5	26.6	1.04
240	98	19.7	27.3	1.04
1440	>99	20.1	27.1	1.05

[a] Determined by ^{1}H NMR spectroscopic analysis comparing the relative integration of polymer and residual monomer.
[b]The 120 min aliquot was contaminated with adventitious polymer and was omitted from SEC.

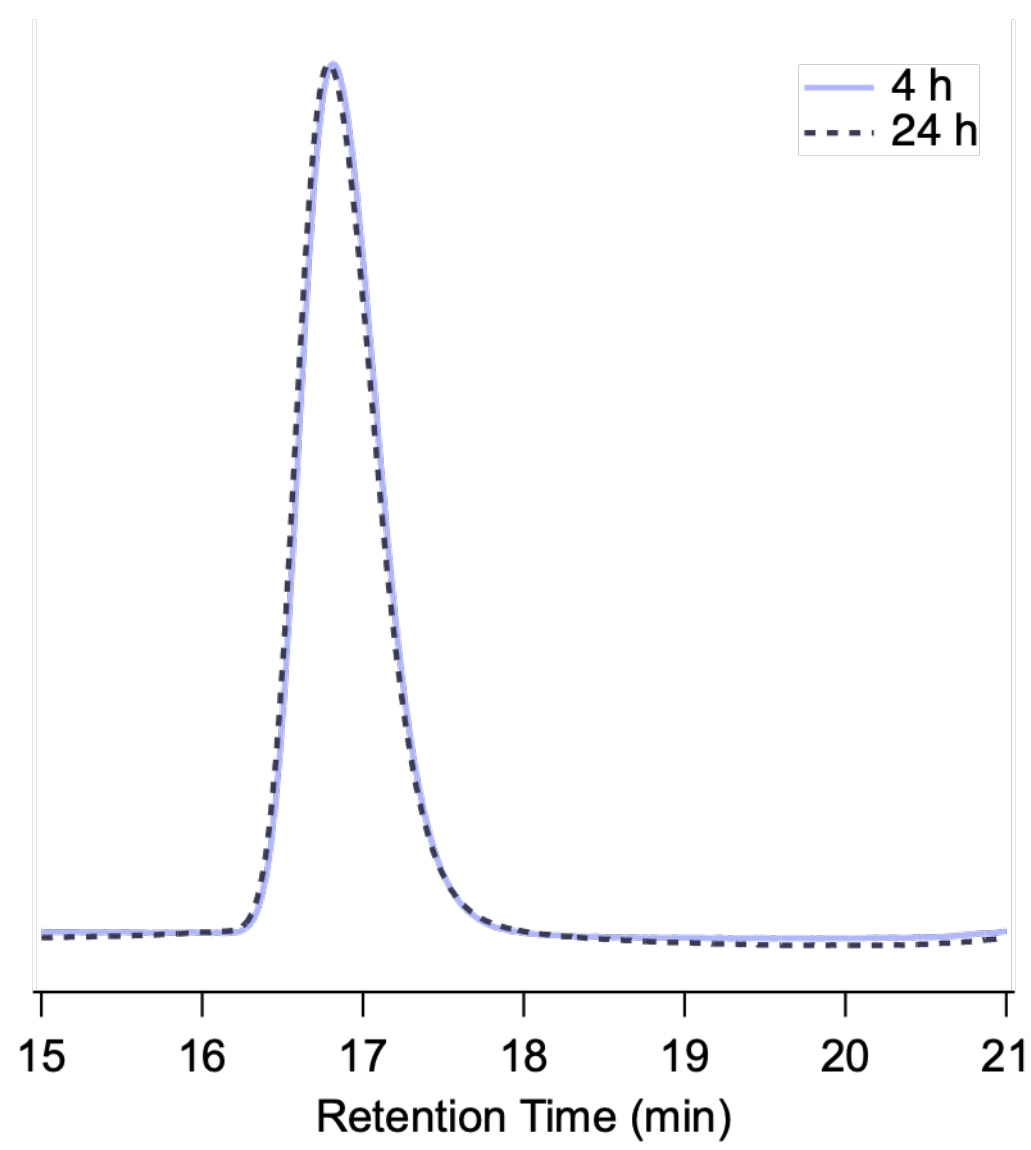

Figure S6: SEC chromatograms of an aliquot near full conversion (98 %) at 4 h and at 24 h from the polymerization of *rac*-*trans*-DMPL by *rac*-**1** ([*trans*-DMPL]$_0$ = 4.0 M toluene, [*trans*-DMPL]$_0$:[*rac*-**1**]$_0$ = 200:1, T_{rxn} = 25 °C).

*The negligible change in SEC trace suggests that after full conversion, rac-**1** does not engage in transesterification and elimination reactions that would contribute to broadened dispersities.*

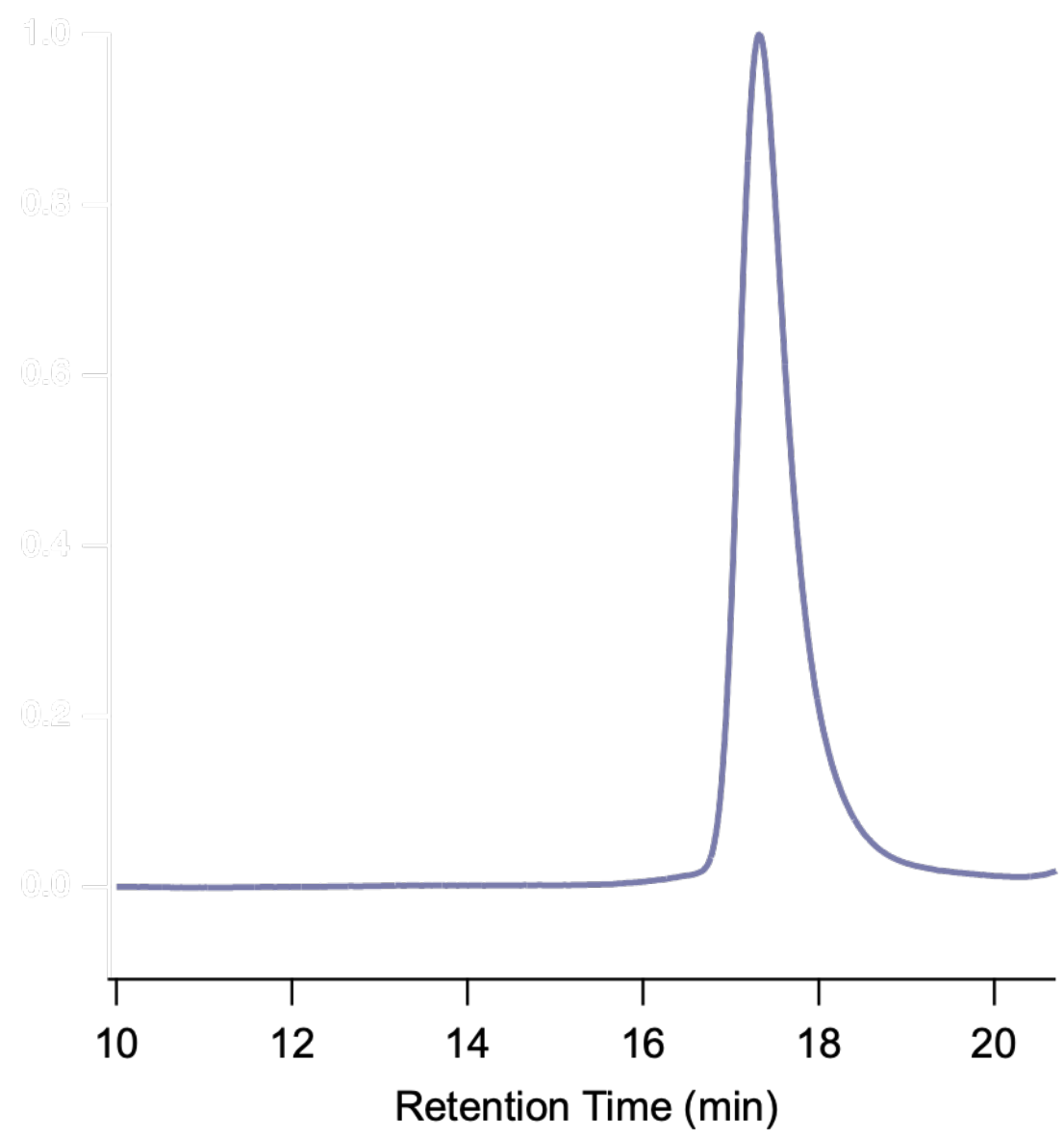

Figure S7: SEC chromatogram from the polymerization of 86 %*ee* (*R,R*)-*trans*-DMPL by [(^{iPr}BDI)ZnO^{i}Pr]$_2$ ([*trans*-DMPL]$_0$ = 4.0 M toluene, [*trans*-DMPL]$_0$:[cat]$_0$ = 200:1, T_{rxn} = 25 °C, t_{rxn} = 24 h). $M_{n,\ theo}$ = 19.4; $M_{n,\ SEC}$ = 18.2; $Đ$ = 1.11.[2]

*Unimodal and narrow molecular weight distribution and molecular weight control indicates enantioenriched monomer is free of substantial quantities of protic impurities. Thus, the abnormal molecular weight distributions made by rac-**1** can be attributed to the differences between the Δ and Λ catalysts.*

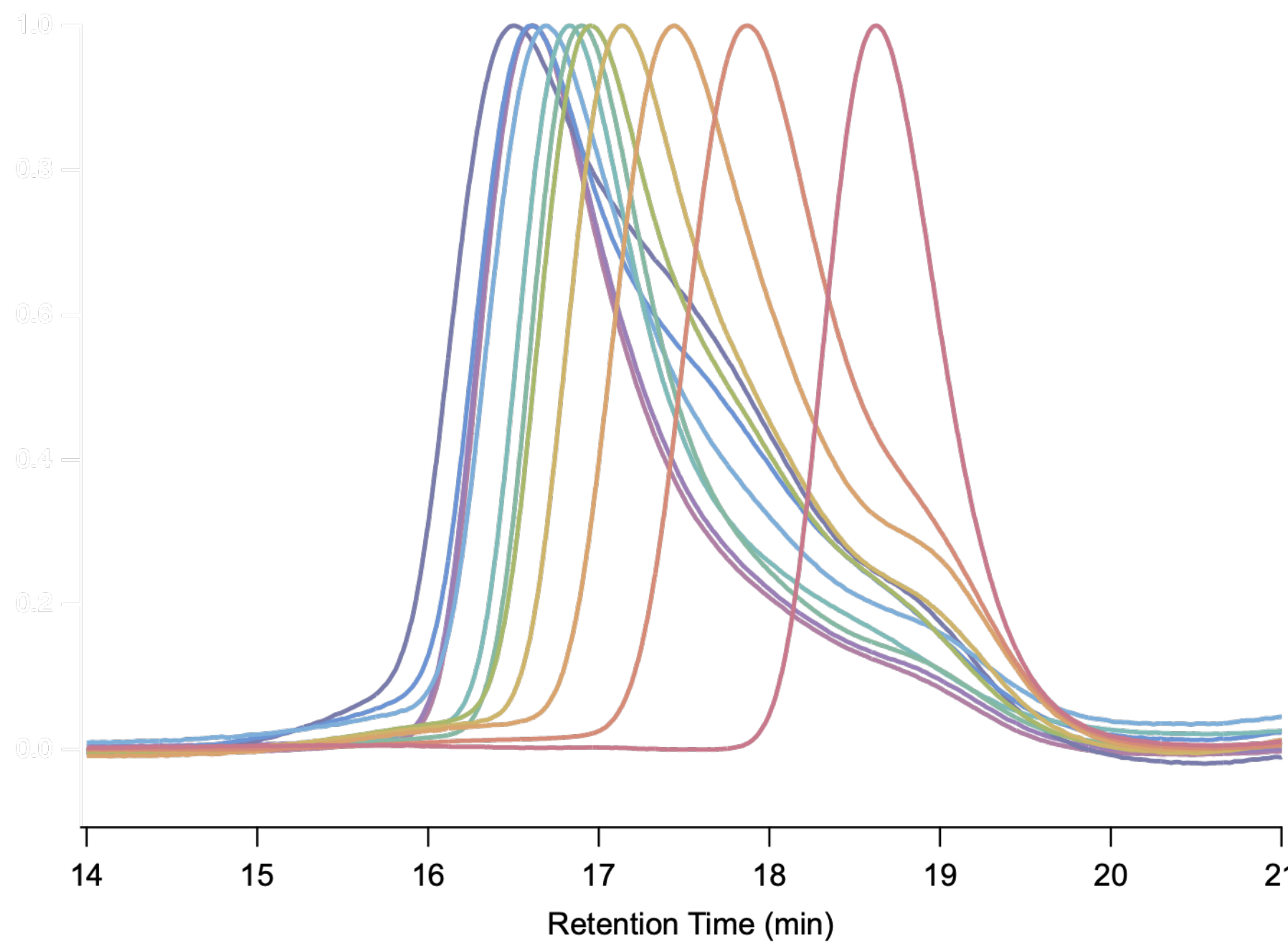

Figure S8: SEC chromatograms of the aliquots from the polymerization of 86 %*ee* (*R,R*)-*trans*-DMPL by *rac*-**1** ([*trans*-DMPL]$_0$ = 4.0 M toluene, [*trans*-DMPL]$_0$:[*rac*-**1**]$_0$ = 200:1, T_{rxn} = 25 °C).

The broadened and polymodal molecular weight distributions support the Δ and Λ catalysts polymerizing at different rates.

Table S5: Conversion and molecular weight data for 86 %*ee* (*R,R*)-*trans*-DMPL kinetics.

Time (min)	Conversion[a]	$M_{n, theo}$ (kDa)	$M_{n, exp}$ (kDa)	*Đ*
20	27	0.0	6.5	1.09
40	42	5.5	9.3	1.27
60	53	8.4	11.4	1.42
80	64	10.6	13.3	1.44
100	73	12.7	14.9	1.45
120	60	14.5	16.6	1.40
140	86	16.0	17.1	1.41
160	85	17.3	17.4	1.52
180	89	17.1	16.9	1.60
200	89	17.8	17.4	1.64
220	94	17.8	18.9	1.50
240	96	18.9	19.7	1.43

[a] Determined by ^{1}H NMR spectroscopic analysis comparing the relative integration of polymer and residual monomer.

3.6 Kinetic Model for Rate Constant Determination

To create a kinetic model for rate-constant determination, we first make a few simplifying assumptions. First, we assume that the stereoselectivity can be fully attributed to the stereochemistry of the catalyst and the chain end, and that there is no influence from the penultimate repeat unit or any repeat units further back in the polymer chain. We also assume that the length of the polymer chain has no effect on the rate of polymerization. In addition, we assume that initiation is unselective and much faster than propagation so that it can be ignored in the kinetic model. Finally, we do not consider termination events or polymer exchange between catalysts. Thus, we can describe the polymerization with the following chemical reactions and their associated rate constants:

$$\Delta_{RR} + \mathrm{M}_{RR} \rightarrow \Delta_{RR} \ \left(k^{\Delta}_{RR\rightarrow RR}\right)$$

$$\Delta_{RR} + \mathrm{M}_{SS} \rightarrow \Delta_{SS} \ \left(k^{\Delta}_{RR\rightarrow SS}\right)$$

$$\Delta_{SS} + \mathrm{M}_{RR} \rightarrow \Delta_{RR} \ \left(k^{\Delta}_{SS\rightarrow RR}\right)$$

$$\Delta_{SS} + \mathrm{M}_{SS} \rightarrow \Delta_{SS} \ \left(k^{\Delta}_{SS\rightarrow SS}\right)$$

$$\Lambda_{RR} + \mathrm{M}_{RR} \rightarrow \Lambda_{RR} \ \left(k^{\Lambda}_{RR\rightarrow RR}\right)$$

$$\Lambda_{RR} + \mathrm{M}_{SS} \rightarrow \Lambda_{SS} \ \left(k^{\Lambda}_{RR\rightarrow SS}\right)$$

$$\Lambda_{SS} + \mathrm{M}_{RR} \rightarrow \Lambda_{RR} \ \left(k^{\Lambda}_{SS\rightarrow RR}\right)$$

$$\Lambda_{SS} + \mathrm{M}_{SS} \rightarrow \Lambda_{SS} \ \left(k^{\Lambda}_{SS\rightarrow SS}\right)$$

Here, Δ_{RR} represents a Δ catalyst with an (*R,R*) chain end, and Δ_{SS}, Λ_{RR}, and Λ_{SS} similarly represent catalysts with the corresponding combination of catalyst and chain-end stereochemistry. M_{RR} and M_{SS} represent *trans*-DMPL monomers with the corresponding (*R,R*) or (*S,S*) stereochemistry.

Based on these chemical reactions, we can write the following system of differential equations to describe the rate of change of the monomer and catalyst species with time:

$$-\frac{d[\mathrm{M}_{RR}]}{dt} = k^{\Delta}_{RR\rightarrow RR}[\mathrm{M}_{RR}][\Delta_{RR}] + k^{\Delta}_{SS\rightarrow RR}[\mathrm{M}_{RR}][\Delta_{SS}] + k^{\Lambda}_{RR\rightarrow RR}[\mathrm{M}_{RR}][\Lambda_{RR}] + k^{\Lambda}_{SS\rightarrow RR}[\mathrm{M}_{RR}][\Lambda_{SS}]$$

$$-\frac{d[\mathrm{M}_{SS}]}{dt} = k^{\Delta}_{SS\rightarrow SS}[\mathrm{M}_{SS}][\Delta_{SS}] + k^{\Delta}_{RR\rightarrow SS}[\mathrm{M}_{SS}][\Delta_{RR}] + k^{\Lambda}_{SS\rightarrow SS}[\mathrm{M}_{SS}][\Lambda_{SS}] + k^{\Lambda}_{RR\rightarrow SS}[\mathrm{M}_{SS}][\Lambda_{RR}]$$

$$\frac{d[\Delta_{RR}]}{dt} = k^{\Delta}_{SS\rightarrow RR}[\mathrm{M}_{RR}][\Delta_{SS}] - k^{\Delta}_{RR\rightarrow SS}[\mathrm{M}_{SS}][\Delta_{RR}]$$

$$\frac{d[\Delta_{SS}]}{dt} = k^{\Delta}_{RR\rightarrow SS}[\mathrm{M}_{SS}][\Delta_{RR}] - k^{\Delta}_{SS\rightarrow RR}[\mathrm{M}_{RR}][\Delta_{SS}]$$

$$\frac{d[\Lambda_{RR}]}{dt} = k^{\Lambda}_{SS\rightarrow RR}[\mathrm{M}_{RR}][\Lambda_{SS}] - k^{\Lambda}_{RR\rightarrow SS}[\mathrm{M}_{SS}][\Lambda_{RR}]$$

$$\frac{d[\Lambda_{SS}]}{dt} = k^{\Lambda}_{RR\rightarrow SS}[\mathrm{M}_{SS}][\Lambda_{RR}] - k^{\Lambda}_{SS\rightarrow RR}[\mathrm{M}_{RR}][\Lambda_{SS}]$$

To simplify the mathematics, we will scale all concentrations so that the total catalyst concentration is 1. Assuming racemic catalyst and no catalyst site inversion, this implies that

$[\Delta_{RR}] + [\Delta_{SS}] = [\Lambda_{RR}] + [\Lambda_{SS}] = 0.5$. By symmetry, we know that $k^{\Delta}_{RR\to RR} = k^{\Lambda}_{SS\to SS}$, $k^{\Delta}_{RR\to SS} = k^{\Lambda}_{SS\to RR}$, $k^{\Delta}_{SS\to RR} = k^{\Lambda}_{RR\to SS}$, and $k^{\Delta}_{SS\to SS} = k^{\Lambda}_{RR\to RR}$. Finally, we have previously determined via the NMR model the selectivity of each chain end, so we know that $\frac{k^{\Delta}_{RR\to RR}}{k^{\Delta}_{RR\to SS}} = 8.84$ and that $\frac{k^{\Delta}_{SS\to SS}}{k^{\Delta}_{SS\to RR}} = 0.99$.

These relationships mean that the system of differential equations can be described with only four variables and two coefficients (rather than six variables and eight coefficients). We will choose to use the variables $[\mathrm{M}_{RR}]$, $[\mathrm{M}_{SS}]$, $[\Delta_{RR}]$, and $[\Lambda_{RR}]$, and the coefficients $k^{\Delta}_{RR\to SS}$ and $k^{\Delta}_{SS\to RR}$. Modeling the kinetics of the polymerization is then equivalent to solving the initial value problem for some specified values of $k^{\Delta}_{RR\to SS}$ and $k^{\Delta}_{SS\to RR}$ and initial concentrations $[\mathrm{M}_{RR}]_0$, $[\mathrm{M}_{SS}]_0$, $[\Delta_{RR}]_0$, and $[\Lambda_{RR}]_0$. Values for the rate constants can be determined by solving for $k^{\Delta}_{RR\to SS}$ and $k^{\Delta}_{SS\to RR}$ by fitting with experimental kinetic data.

We applied this approach to determine the rate constants from the kinetics of two polymerizations, one with racemic monomer and one with enantioenriched (86 *%ee*) (*R,R*) monomer. Both polymerizations used racemic catalyst and a monomer to catalyst ratio of 200:1. Only the first 100 minutes of data were used as deviations from first-order behavior were noted later in the polymerizations, possibly due to the changing viscosity and homogeneity of the solution. The collection of this kinetic data is described in Section 2.4 and the results are reported in section 3.5.

To fit the data, we wrote a Python script implementing the mathematical approach described above. Full details of this implementation are provided as annotations to the code, available as a Python notebook in the supplementary zip file. As an overview, one function implements the system of differential equations, taking as input the values of the four variables and two coefficients, and returning the derivative of each variable. The initial value problem is solved numerically using the SciPy function 'solve_ivp,' which implements the Runge-Kutta method. The error of the fit is given by the sum of the squared difference between the log of the measured concentration and the log of the predicted concentration for each data point, as when fitting standard pseudo-first-order kinetics. Best-fit values of $k^{\Delta}_{RR\to SS}$ and $k^{\Delta}_{SS\to RR}$ are determined by minimizing the error function using the SciPy function 'minimize' to implement the Nelder-Mead algorithm. Best-fit values were also determined for the initial value of each variable.

Applying this gives best-fit values of $k^{\Delta}_{RR\to RR} = 27.7$, $k^{\Delta}_{RR\to SS} = 3.13$, $k^{\Delta}_{SS\to RR} = 3.13$, and $k^{\Delta}_{SS\to SS} = 3.10$ $\mathrm{mM^{-1}\cdot s^{-1}}$. However, it does not provide information about the uncertainty associated with these values. To capture this uncertainty, we used the Monte Carlo method, where synthetic datasets are generated from the experimental data based on the experimental uncertainty associated with those datapoints. Fitting many synthetic datasets produces a distribution of output values. To determine the uncertainty of the model output, we can use the statistical properties (e.g. standard deviation) of this Monte Carlo distribution.[9,10]

The synthetic data sets were generated as follows. Each synthetic data point was chosen randomly from a normal distribution with a mean of the experimental value and a chosen standard deviation. For the time at which aliquots were taken, the standard deviation was chosen as 0.25 minutes. For the monomer concentrations, standard deviations were determined from the root-mean-square error of each best-fit curve (i.e. separate error values were determined for the (*R,R*) and (*S,S*) concentrations of the enantioenriched kinetics and for the racemic kinetics). Because the root-mean-square error was determined from a fit to the log of the concentration, the actual standard deviation for each data point is the root-mean-square error multiplied by the measured

concentration. The root-mean-square errors were 0.0306 for (*R,R)*, 0.0588 for (*S,S*), and 0.0513 for the racemic kinetics. Finally, the standard deviations for the chain-end selectivities were determined from fitting NMR spectra of multiple polymers as described in **Table S3**, and were 0.27 for $\frac{k^{\Delta}_{RR\to RR}}{k^{\Delta}_{RR\to SS}}$ and 0.12 for $\frac{k^{\Delta}_{SS\to SS}}{k^{\Delta}_{SS\to RR}}$.

Generating and fitting 1000 synthetic data sets gave the following values for the rate constants, with uncertainties expressed as the standard deviation of the mean: $k^{\Delta}_{RR\to RR} = 27.6 \pm 1.3$, $k^{\Delta}_{RR\to SS} = 3.13 \pm 0.17$, $k^{\Delta}_{SS\to RR} = 3.2 \pm 0.4$, and $k^{\Delta}_{SS\to SS} = 3.2 \pm 0.6$ $mM^{-1}\cdot s^{-1}$. It is worth noting that the means from these Monte Carlo simulations differ very slightly from the best-fit values due to non-zero skewness of the distributions. For our final values, we report the best-fit value with uncertainty derived from the Monte Carlo simulation. Histograms showing the full distributions of these values are shown in **Figure S9**, and scatterplots showing their correlations are shown in **Figure S10**. Additionally, the proportion of Δ catalysts resting with (*R,R*) chain ends was determined to be 50% ± 4%. A histogram of this distribution is shown in **Figure S11**.

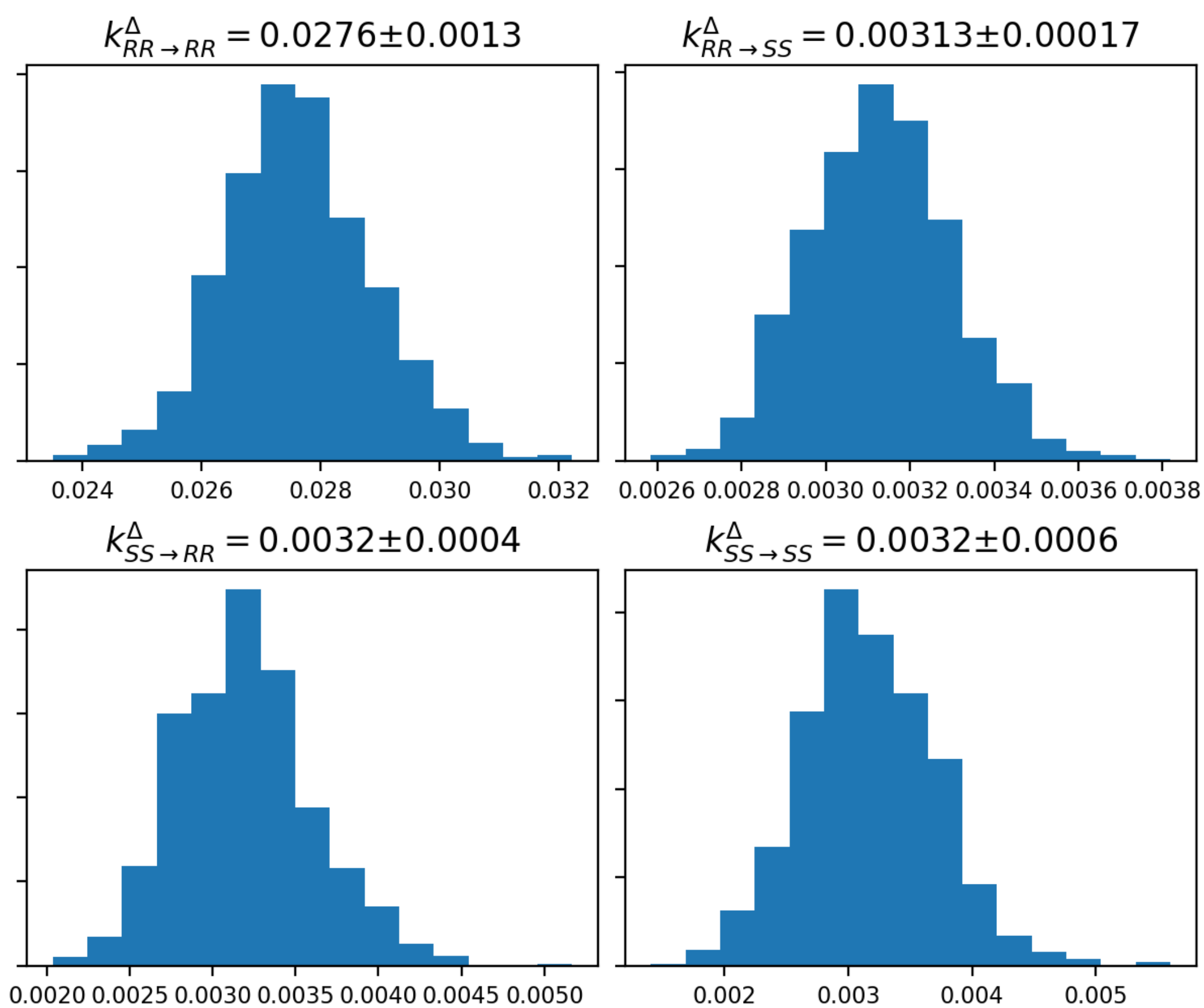

Figure S9: Histograms showing the distributions of rate constants ($M^{-1}\cdot s^{-1}$) determined from 1000 Monte Carlo simulations.

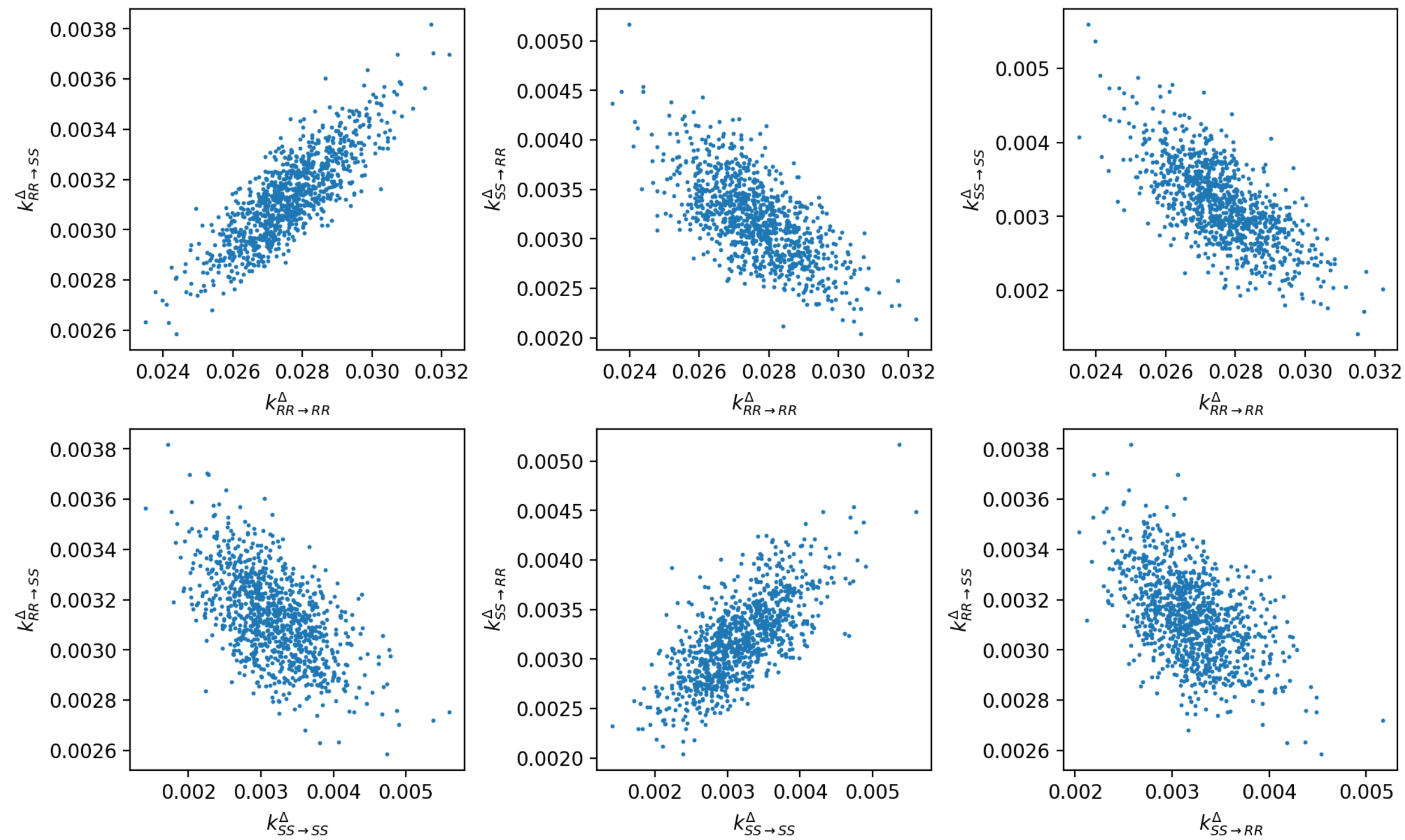

Figure S10: Scatterplots showing correlations between the rate constants ($M^{-1}\bullet s^{-1}$) determined from 1000 Monte Carlo simulations.

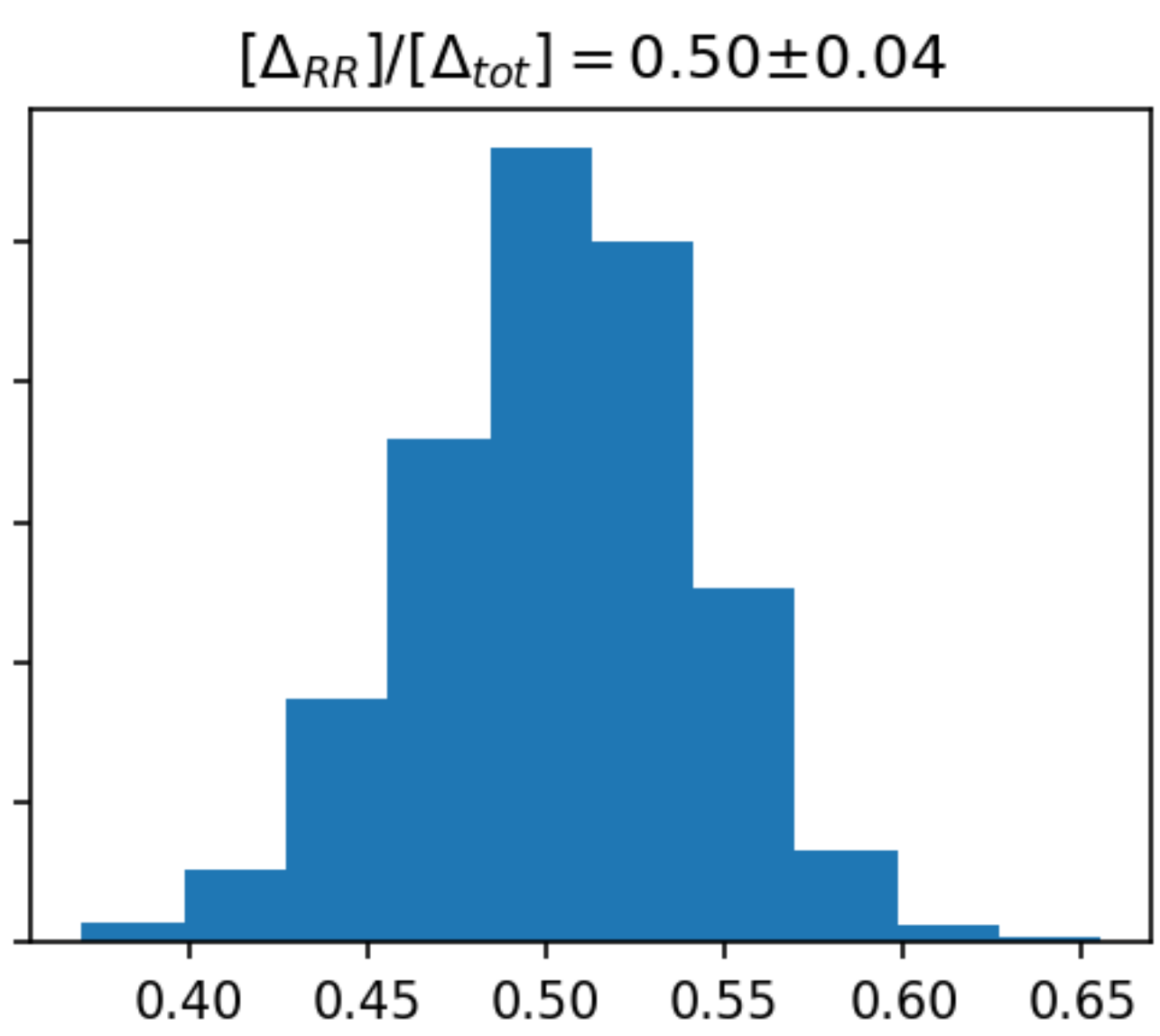

Figure S11: A histogram showing the distribution of values for the proportion of Δ catalysts with (*R,R*) chain ends, determined from 1000 Monte Carlo simulations.

4 NMR Spectra

4.1 BDIZnO^{i}Pr Complexes

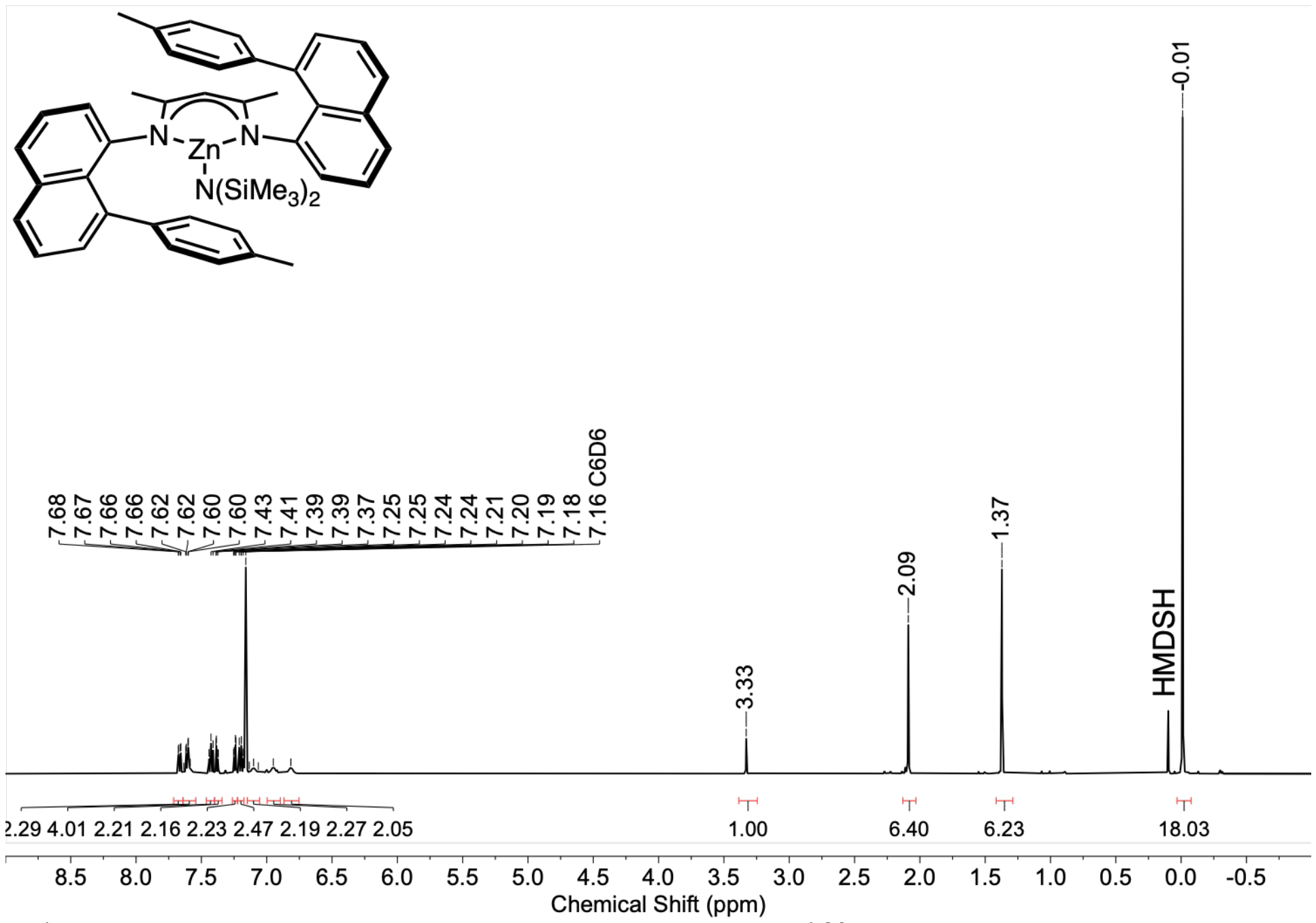

Figure S12: ^{1}H NMR (500 MHz, C_6D_6) spectrum of *rac*-($^{4\text{-Me}}$BDI*)Zn(HMDS).

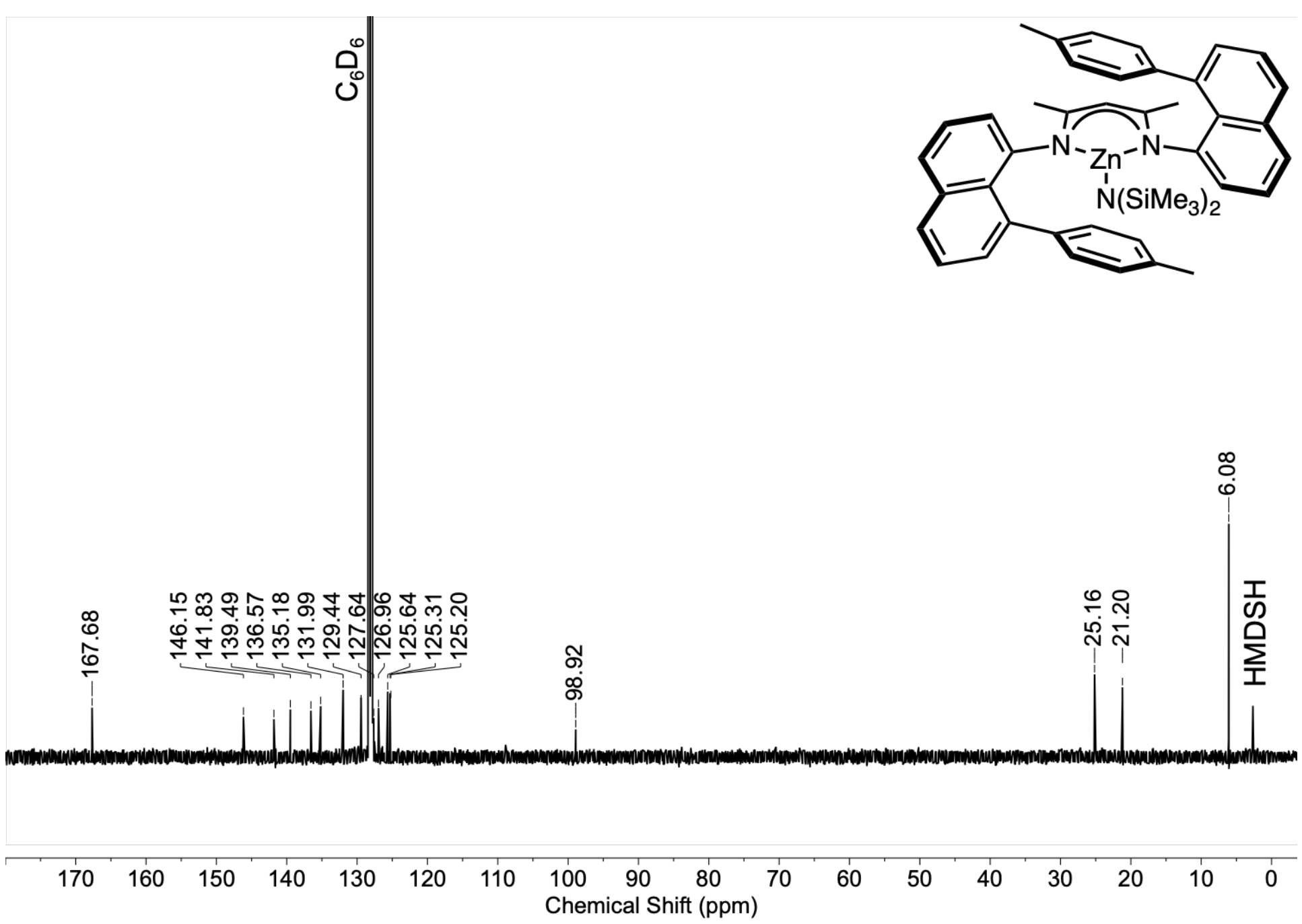

Figure S13: $^{13}C\{^{1}H\}$ NMR (126 MHz, C_6D_6) spectrum of *rac*-($^{4\text{-Me}}$BDI*)Zn(HMDS).

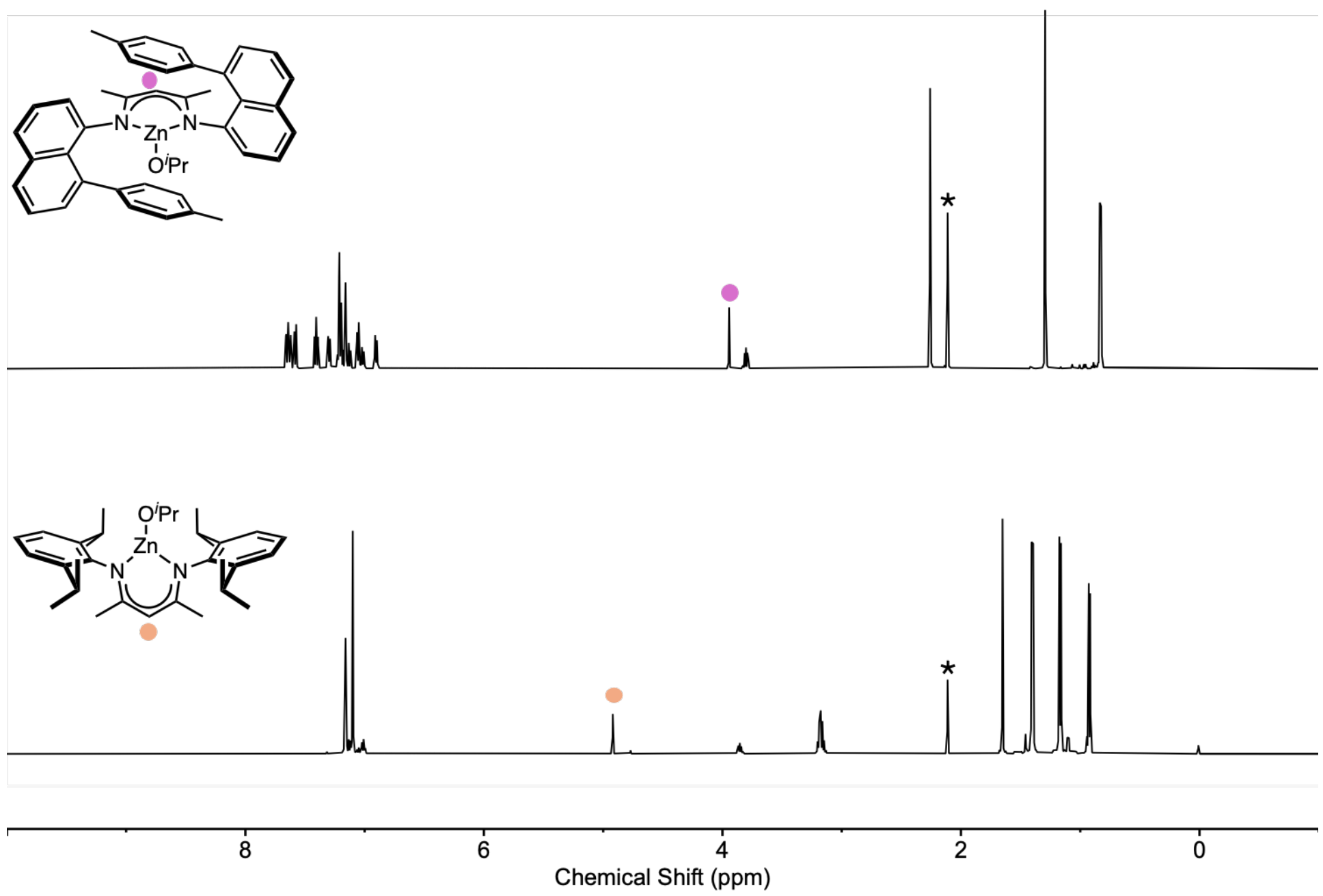

Figure S14: ^{1}H NMR (500 MHz, C_6D_6) spectra of ($^{4\text{-Me}}$BDI*)ZnO^{i}Pr and [(^{iPr}BDI)ZnO^{i}Pr]$_2$ comparing the relative shifts of the backbone methine protons. Full characterization of these compounds has been previously reported.[2] *Indicates residual toluene

4.2 Selected *trans*-PHMB ^{13}C{^{1}H} NMR Spectra

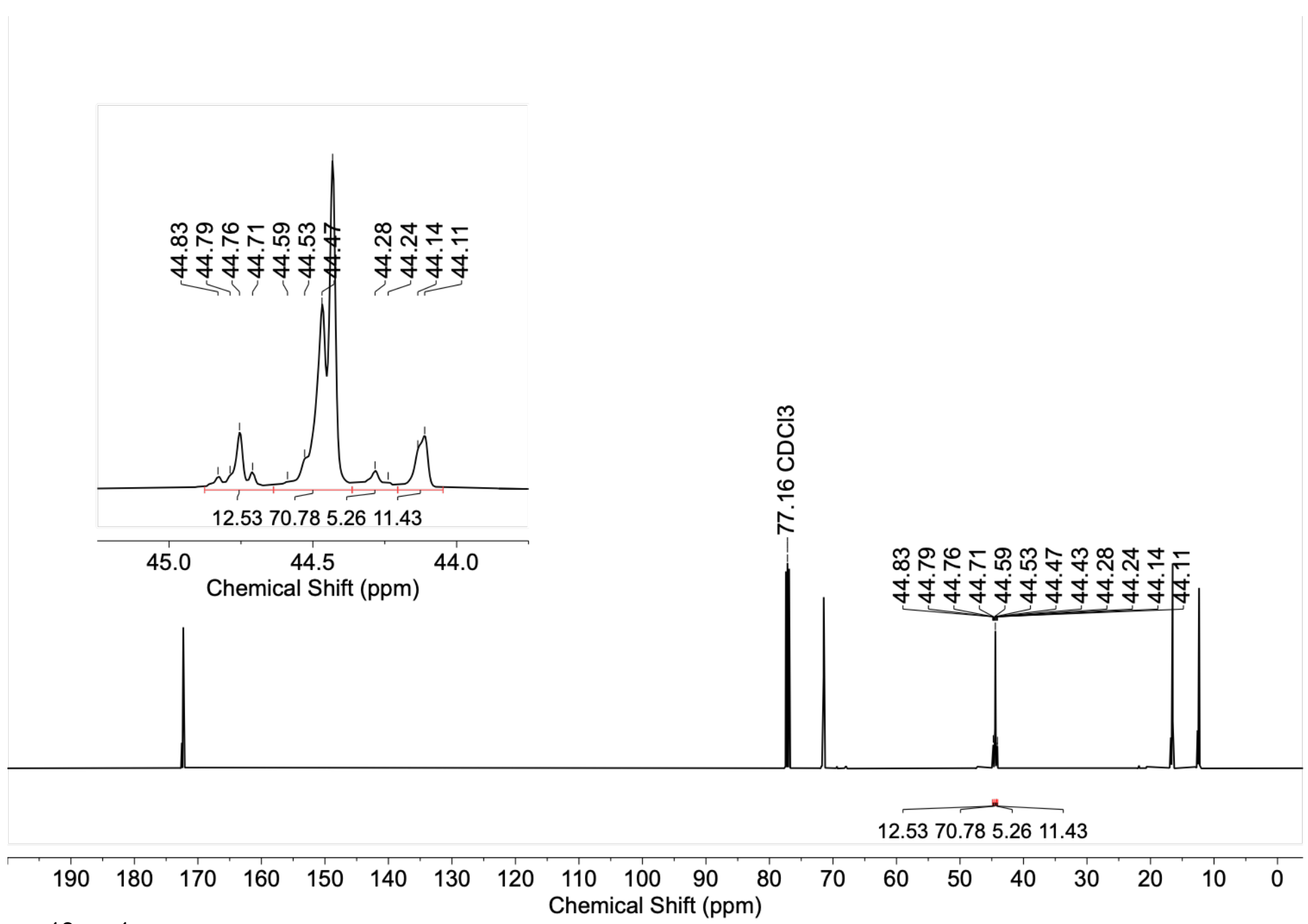

Figure S15: ^{13}C{^{1}H} NMR (126 MHz, $CDCl_3$) spectrum of isoenriched *trans*-PHMB (*mm*% = 71 %; Table 1, entry 1).

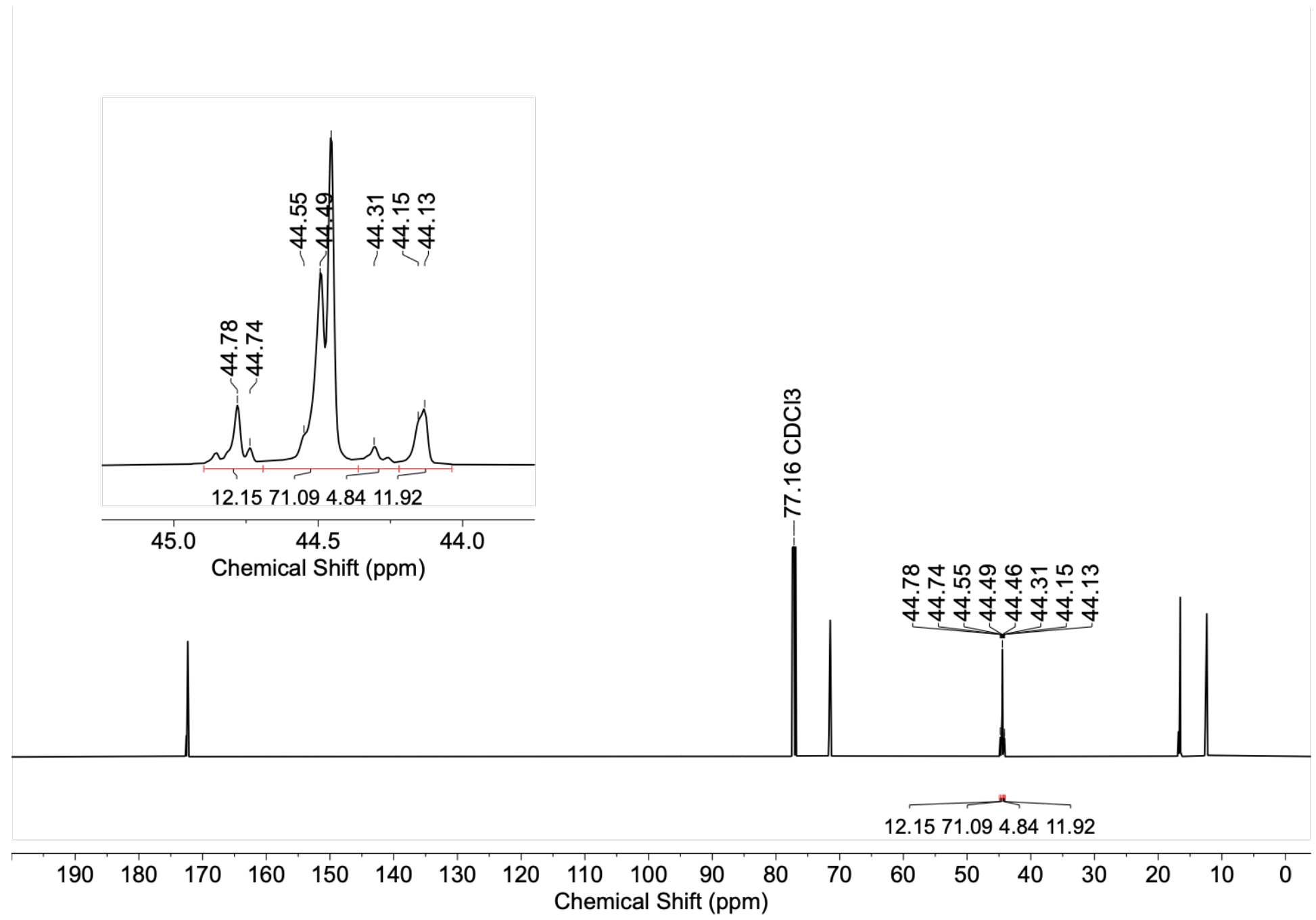

Figure S16: $^{13}C\{^{1}H\}$ NMR (126 MHz, $CDCl_3$) spectrum of isoenriched *trans*-PHMB (*mm*% = 71 %; Table 1, entry 2).

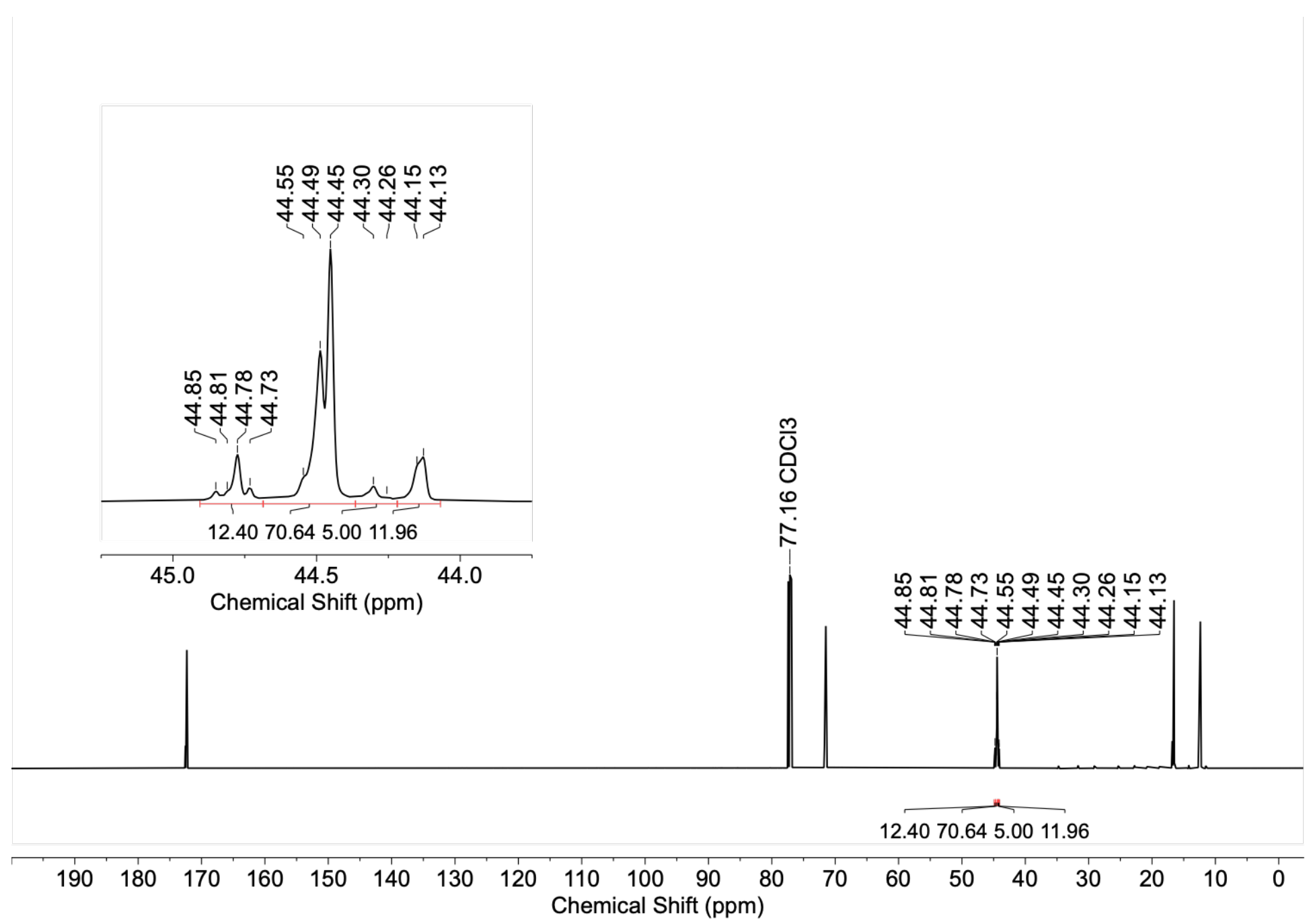

Figure S17: $^{13}C\{^{1}H\}$ NMR (126 MHz, $CDCl_3$) spectrum of isoenriched *trans*-PHMB (*mm*% = 71 %; Table 1, entry 3).

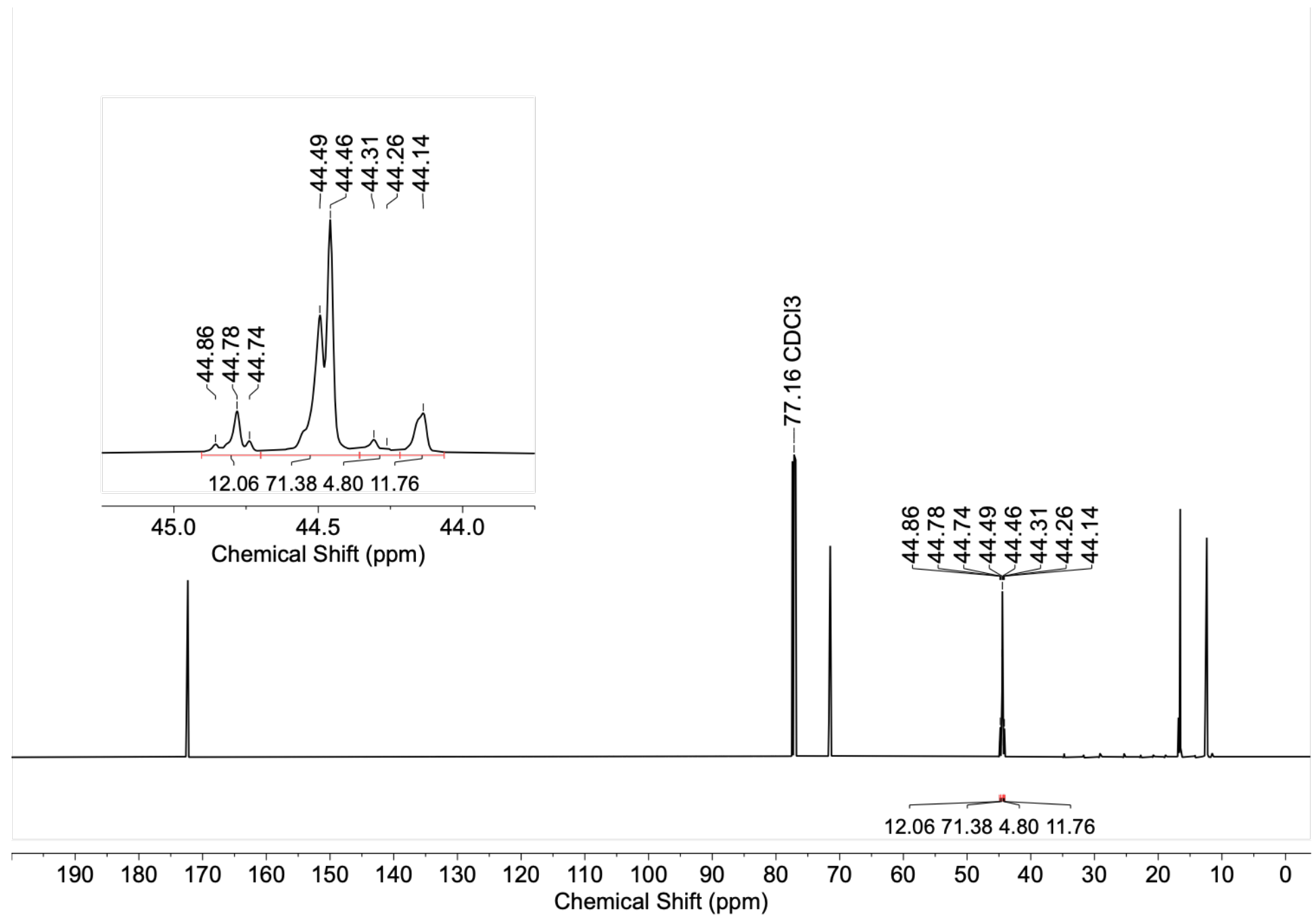

Figure S18: $^{13}C\{^{1}H\}$ NMR (126 MHz, $CDCl_3$) spectrum of isoenriched *trans*-PHMB (*mm*% = 71 %; Table 1, entry 4).

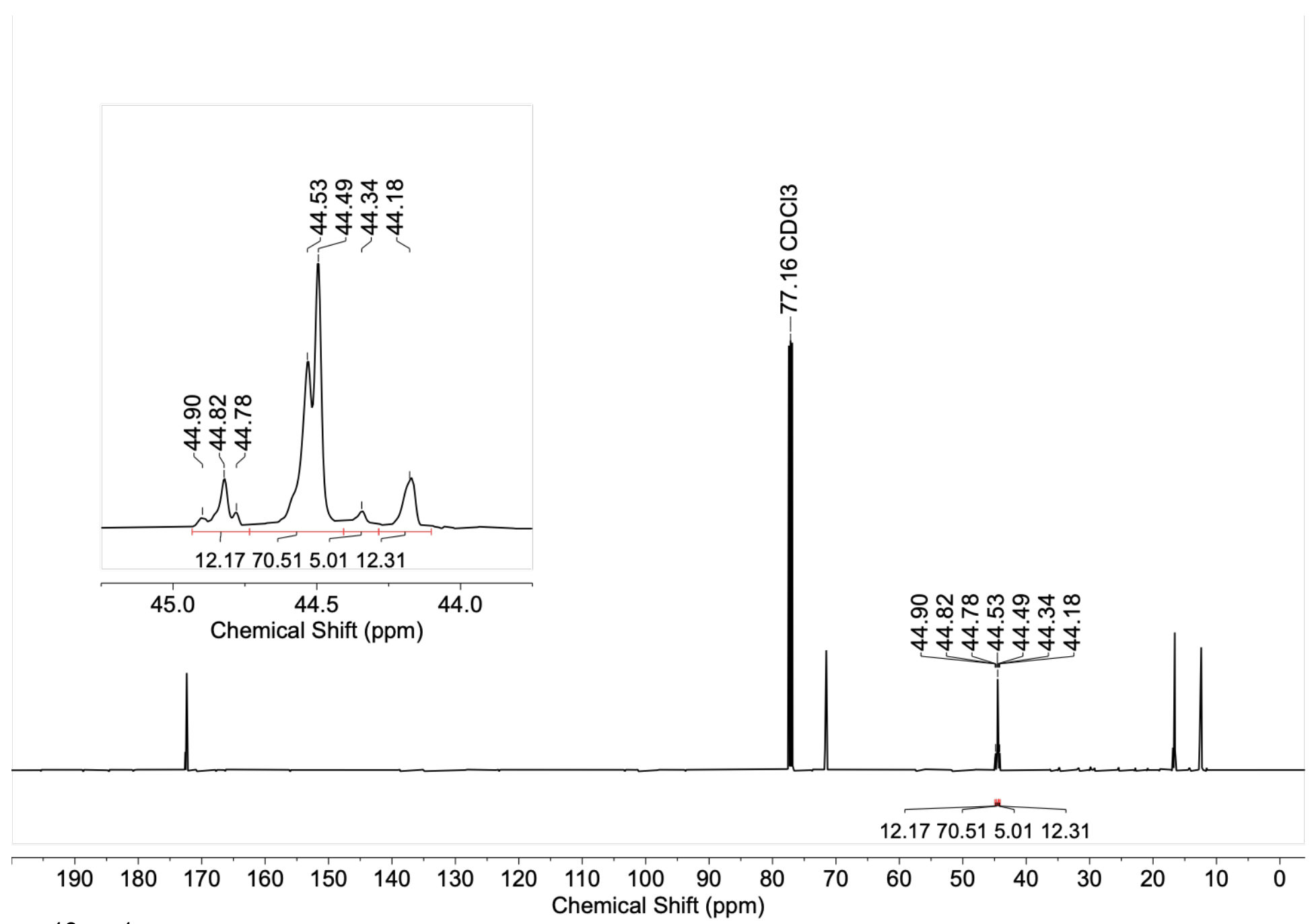

Figure S19: $^{13}C\{^{1}H\}$ NMR (126 MHz, $CDCl_3$) spectrum of isoenriched *trans*-PHMB (*mm*% = 71 %; Table 1, entry 5).

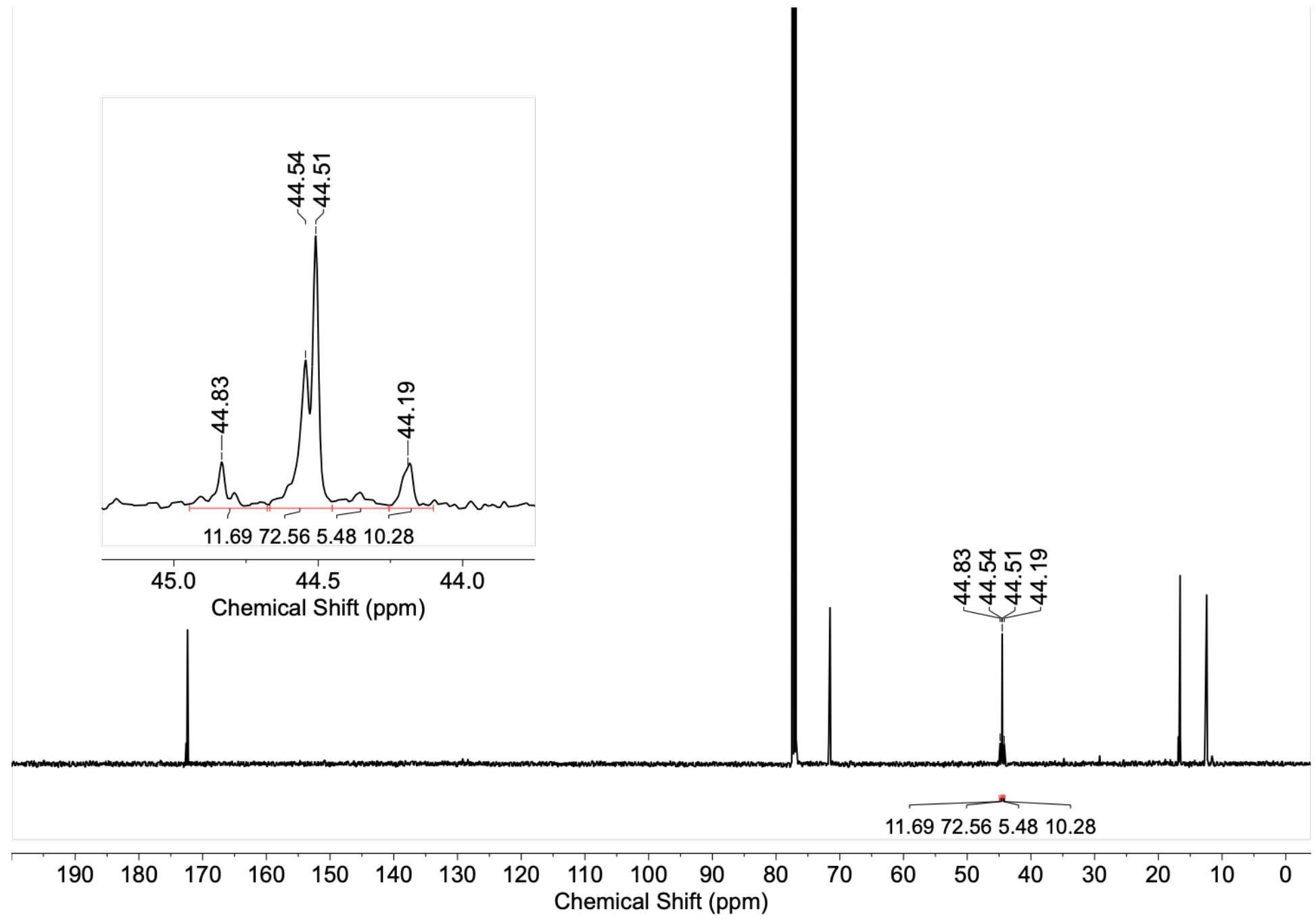

Figure S20: $^{13}C\{^{1}H\}$ NMR (126 MHz, $CDCl_3$) spectrum of isoenriched *trans*-PHMB (*mm*% = 73 %; Table 1, entry 6).

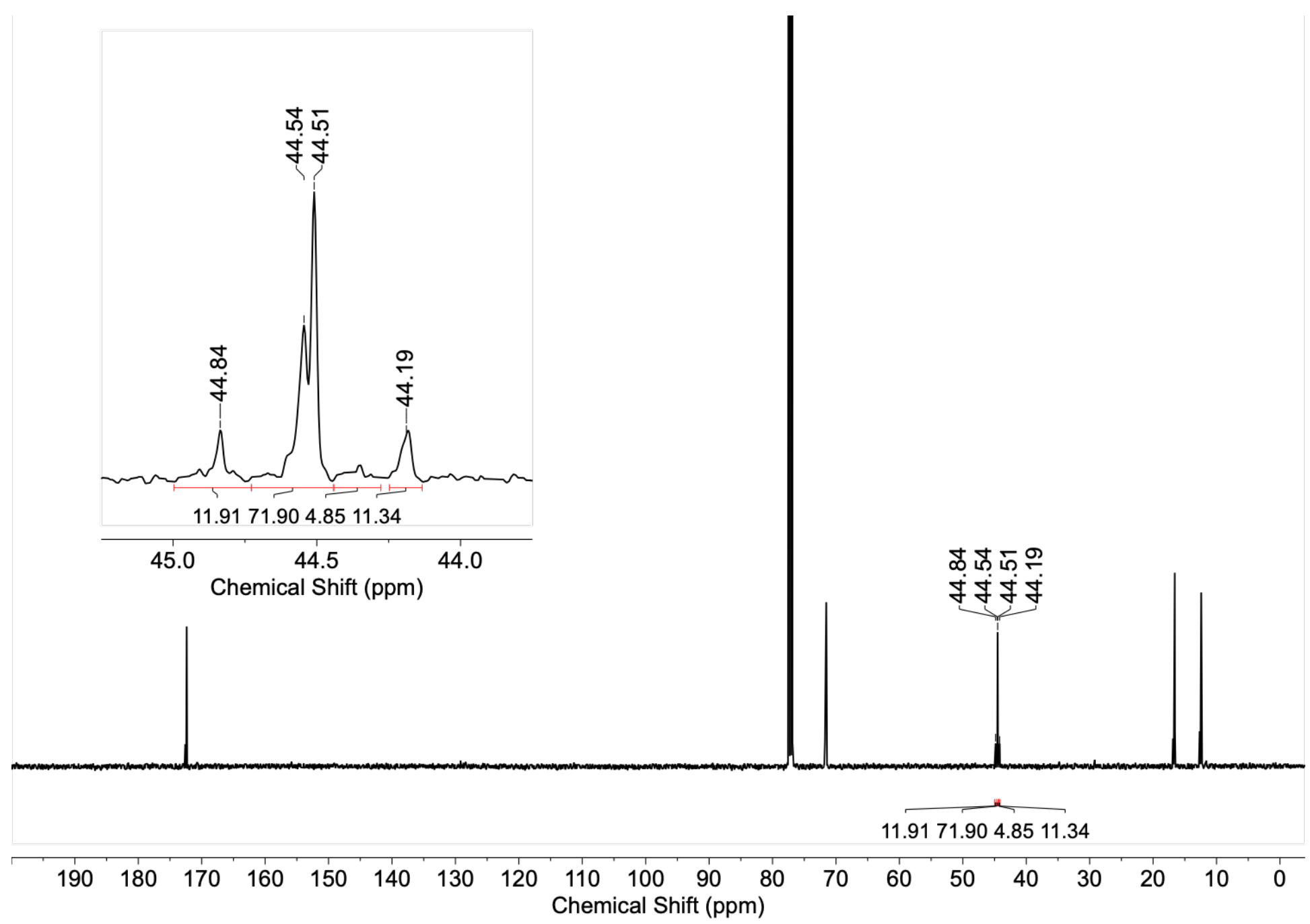

Figure S21: $^{13}C\{^{1}H\}$ NMR (126 MHz, $CDCl_3$) spectrum of isoenriched *trans*-PHMB (*mm*% = 72 %; Table 1, entry 7).

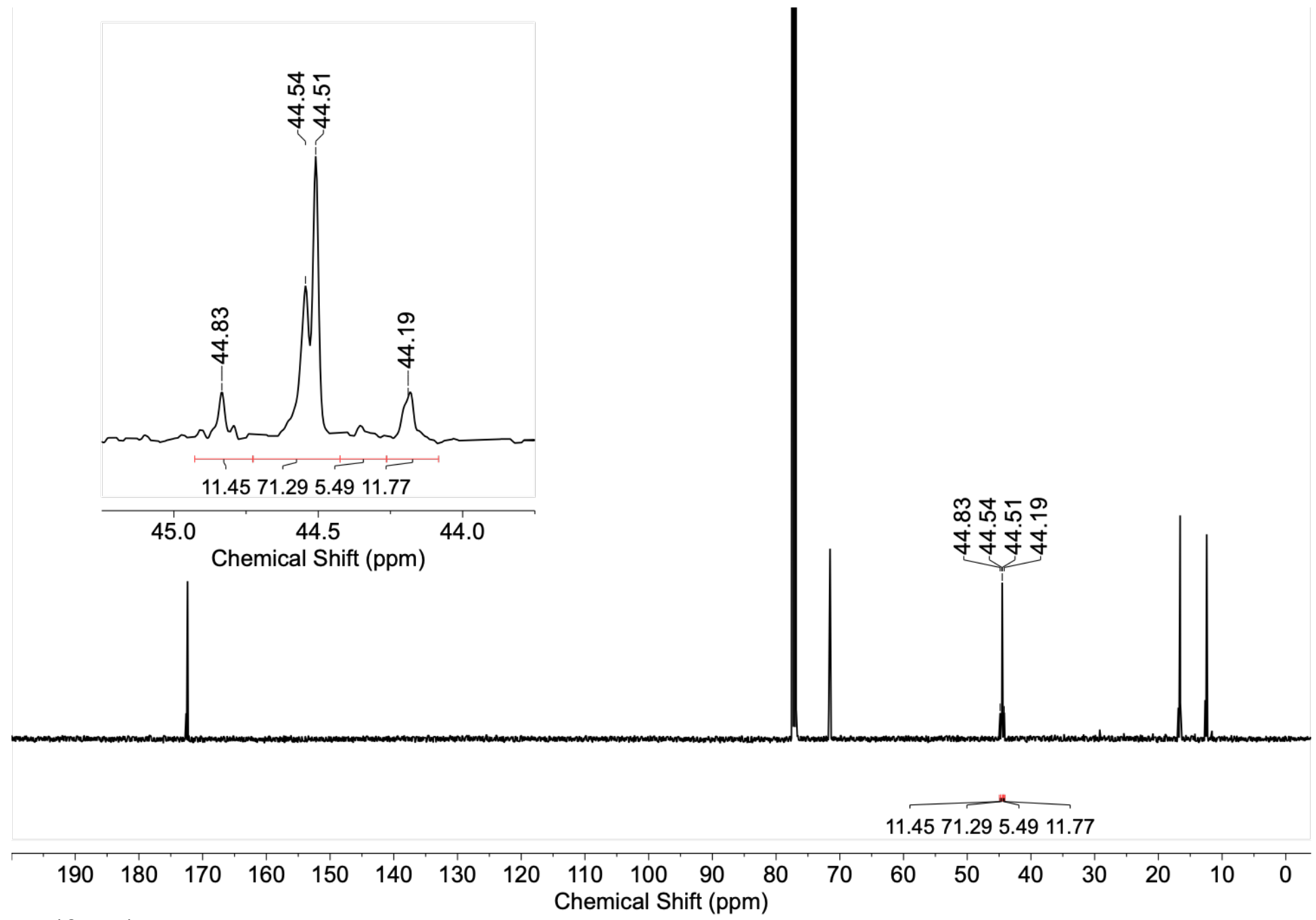

Figure S22: $^{13}C\{^{1}H\}$ NMR (126 MHz, $CDCl_3$) spectrum of isoenriched *trans*-PHMB (*mm*% = 71 %; Table 1, entry 8).

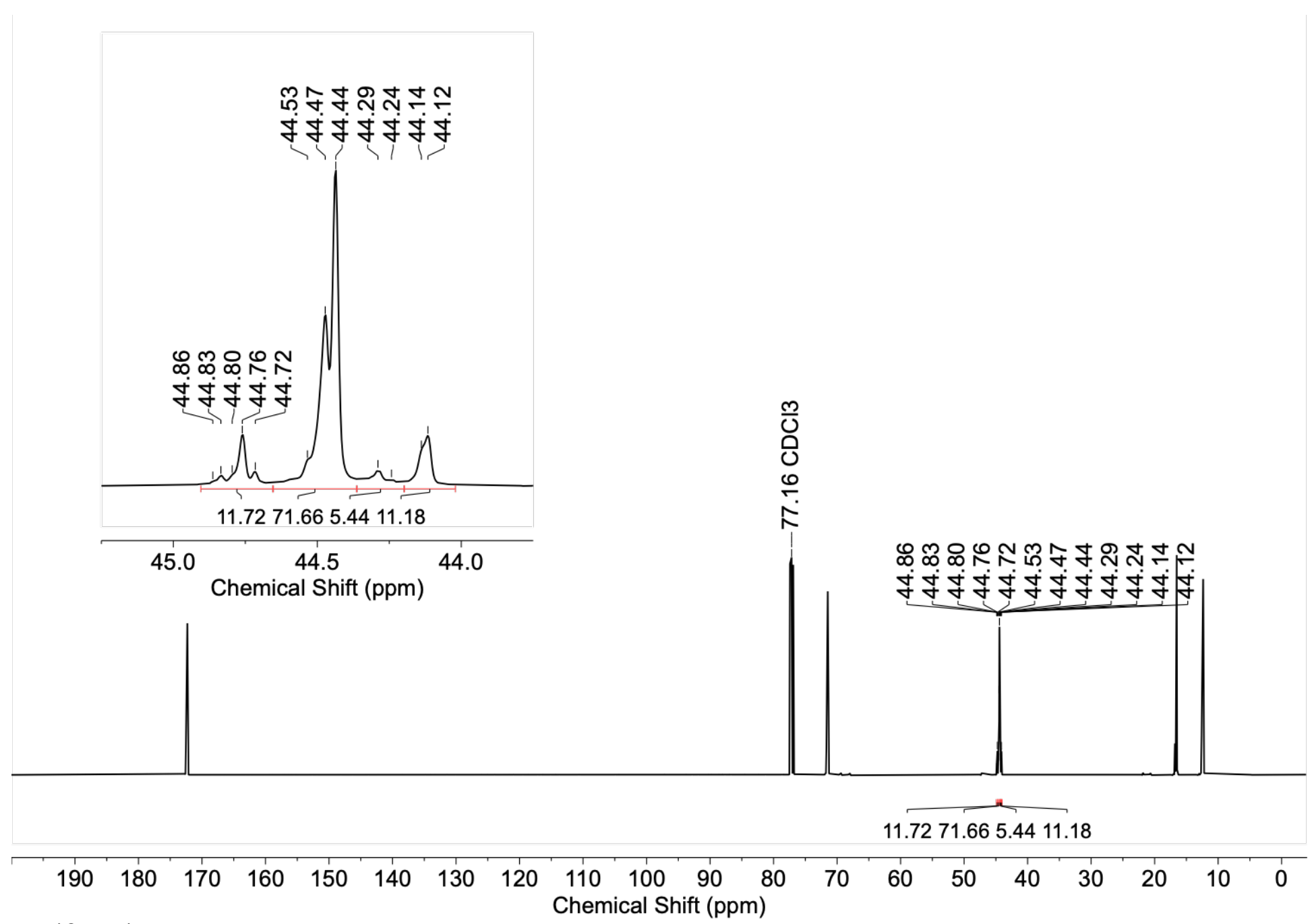

Figure S23: $^{13}C\{^{1}H\}$ NMR (126 MHz, $CDCl_3$) spectrum of isoenriched *trans*-PHMB (*mm*% = 72 %; Table 1, entry 9).

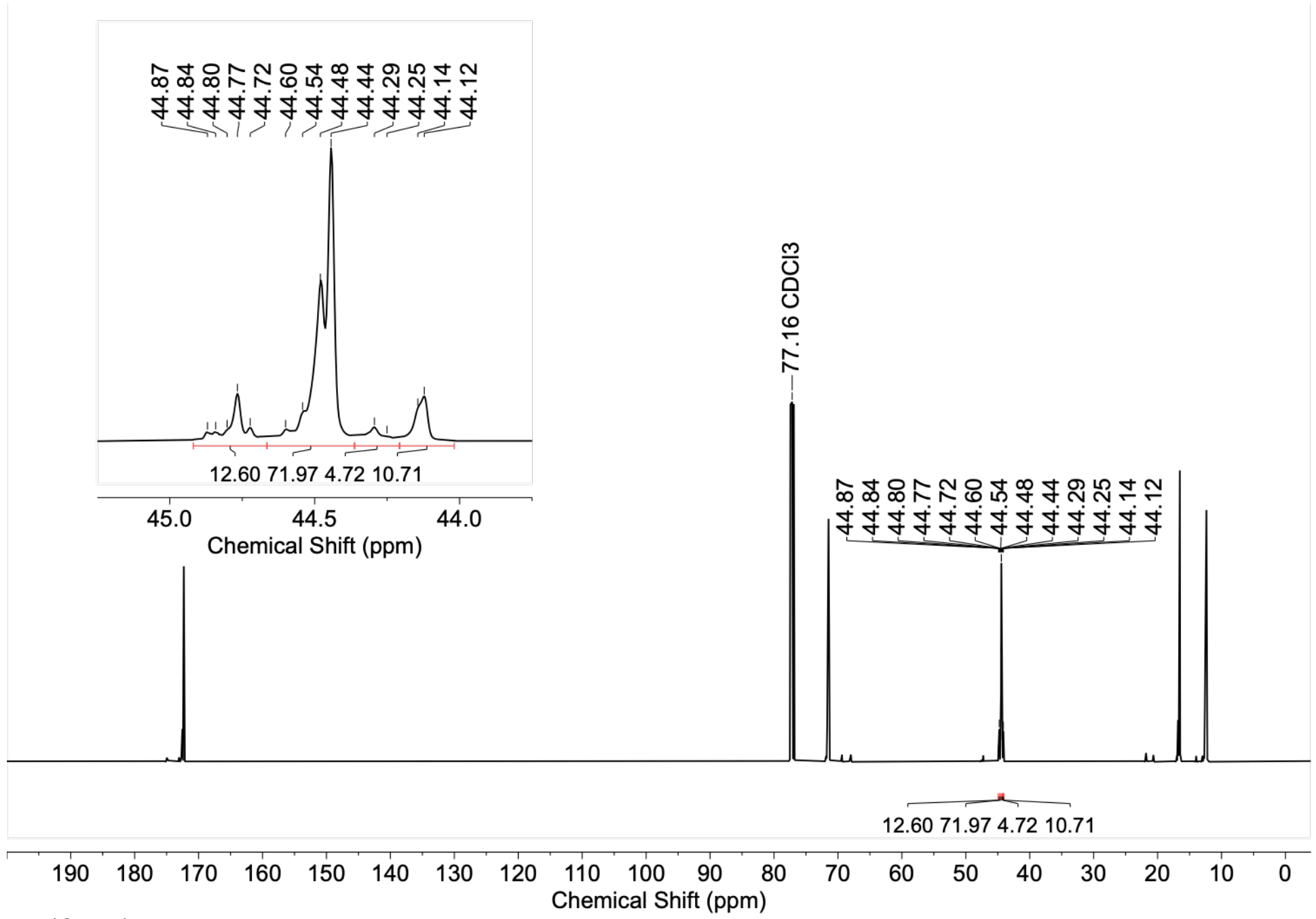

Figure S24: $^{13}C\{^{1}H\}$ NMR (126 MHz, $CDCl_3$) spectrum of isoenriched *trans*-PHMB (*mm*% = 72 %; Table 1, entry 10).

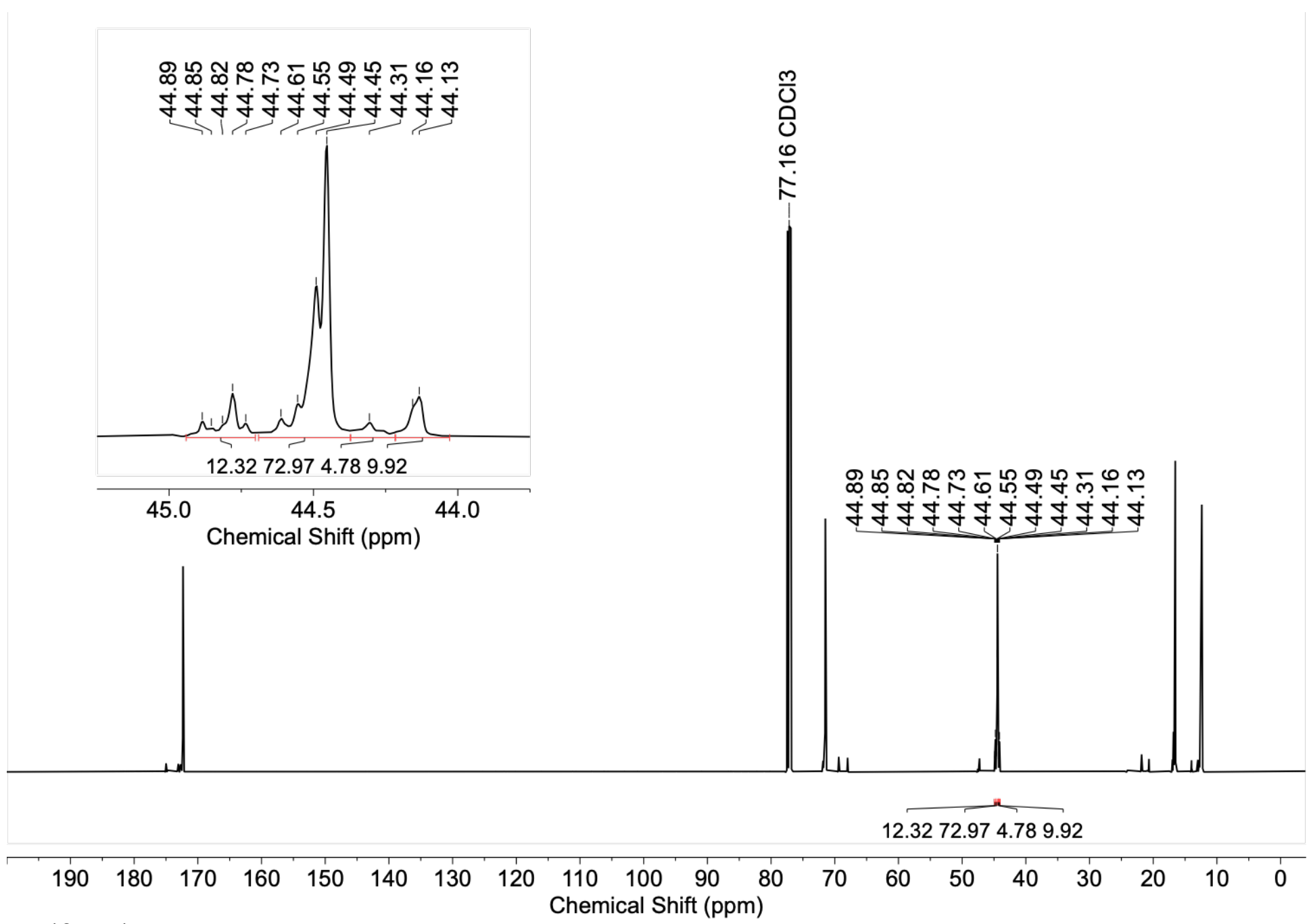

Figure S25: $^{13}C\{^{1}H\}$ NMR (126 MHz, $CDCl_3$) spectrum of isoenriched *trans*-PHMB (*mm*% = 73 %; Table 1, entry 11).

5 Computational Details
5.1 General Information

All stationary points were fully characterized by vibrational frequency analysis at the same level of theory, confirming minima (no imaginary frequencies) and transition states (one imaginary frequency). Frequency calculations were also used to obtain zero-point energy (ZPE) corrections and thermal corrections to enthalpy and entropy at 298.15 K and 1 bar. Final Gibbs free energies (ΔG) reported in the manuscript were obtained by combining single-point electronic energies at the PBE0[11]-D3BJ[12]/SDD[13](Zn)/6-311G(d,p)[14,15] level with thermal corrections derived from the B3LYP[16]/SDD(Zn)/Def2SVP[17] frequency calculations. Benchmark calculations with alternative functionals (B3LYP-D3BJ, M06,[18] and ωB97X-D[19]) confirmed that the predicted stereoselectivity is not affected by the choice of functional (**Table S6**).

Non-covalent interaction (NCI) analysis was performed using the NCIPLOT 4 software,[20] inputting the cartesian coordinates of the molecule of interest. A fine grid integration was employed in the calculations. VMD software[21] was used to visualize the isosurfaces generated, choosing an isovalue of 0.3 and a color scale $-0.03 < \rho < 0.03$.

The buried volume ($\%V_{Bur}$) analysis was performed with the SambVca 2.1 tool,[22] by using a sphere radius of 5.0 Å and mesh size of 0.10. H atoms were not included in the calculation. We used: Zn atom as center of the sphere; C2 atom of the ligand backbone as negative z; C1 atom of the ligand naphthyl ring for xz-plane definition. Values reported in **Figure S33** are the occupations of the octants in the positive hemisphere, where the transition state is occurring.

The activation strain model analysis (ASM)[23] has been performed separating the transition states of interest into two fragments. The catalyst precursor + growing chain has been chosen as Fragment 1 (**Figure S31**, left), the monomer as Fragment 2 (**Figure S31**, right). Single-point calculations of each fragment have been performed using the methods reported in the main text, but without solvent.

$$\Delta E_{\text{Strain (Frag.1)}} = \Delta E_{\text{(Frag.1)}} - \Delta E_{\text{(Reference)}}$$

$$\Delta E_{\text{Strain (Frag.2)}} = \Delta E_{\text{(Frag.2)}} - \Delta E_{\text{(Reference)}}$$

ΔE_{Strain} has then been calculated as the sum of the ΔE_{Strain} values of each fragment:

$$\Delta E_{\text{Strain (TOT)}} = \Delta E_{\text{Strain (Frag.1)}} + \Delta E_{\text{Strain (Frag.2)}}$$

5.2 Benchmark Calculations with Alternative Functionals

Table S6: Stereoselectivities (Gibbs energies, $\Delta\Delta G^{\ddagger}_{stereo}$ in kcal·mol^{-1}) for *trans*-DMPL ROP promoted by *rac*-**1** obtained with different functionals in the single-point calculations.

	SP functional			
	PBE0-D3BJ	B3-D3BJ	M06	ωB97XD
$\Delta\Delta G^{\ddagger}_{stereo}$ (kcal·mol^{-1})	1.3	1.6	1.5	1.2

5.3 Noncovalent Interaction (NCI) Analysis

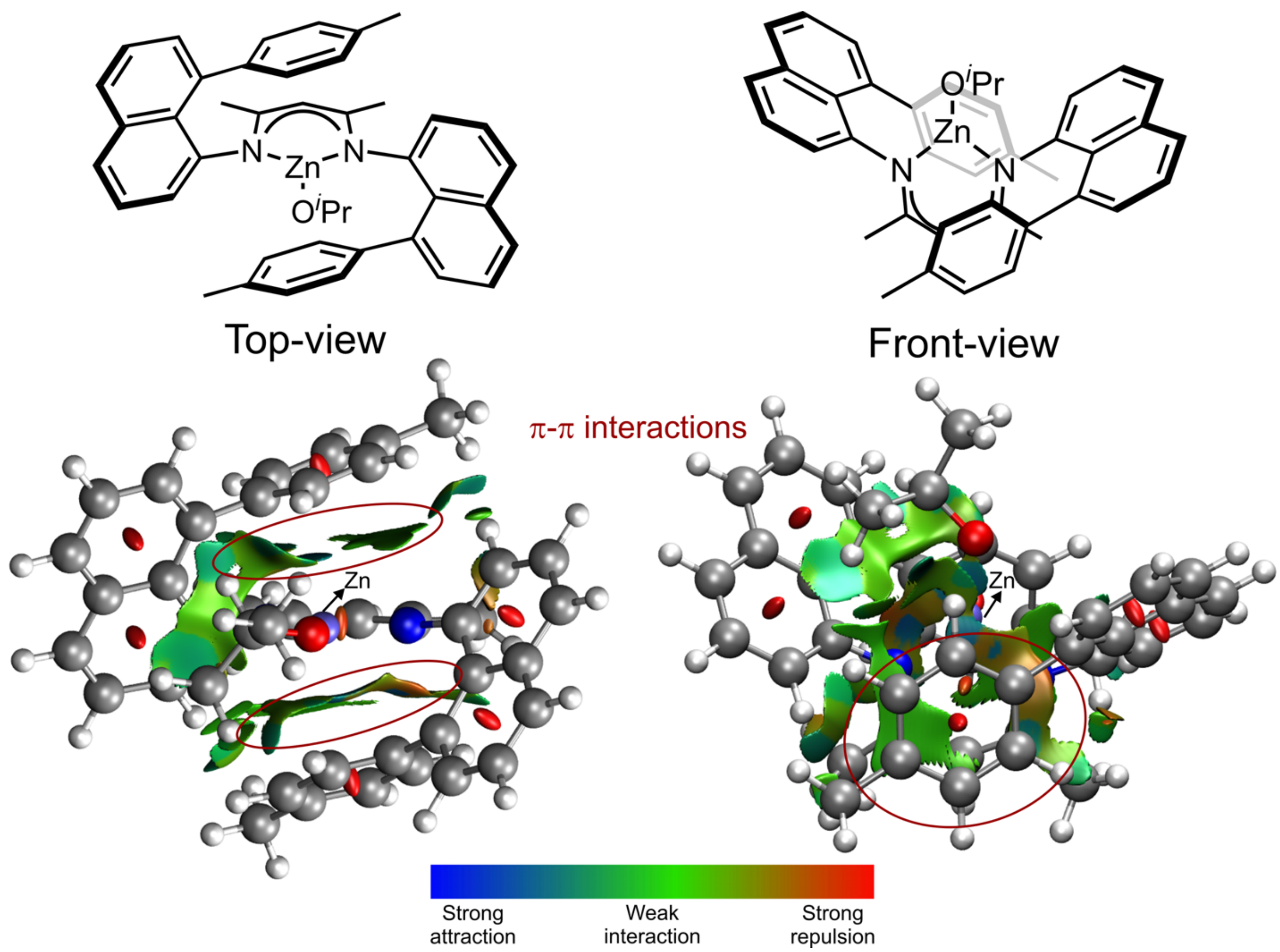

Figure S26: NCI analysis of *rac*-**1** mononuclear precursor from a top (left) and front-view (right), highlighting the presence of π-π interactions that stabilize this species.

5.4 Comparison of Mononuclear and Dinuclear Structures of *rac*-1

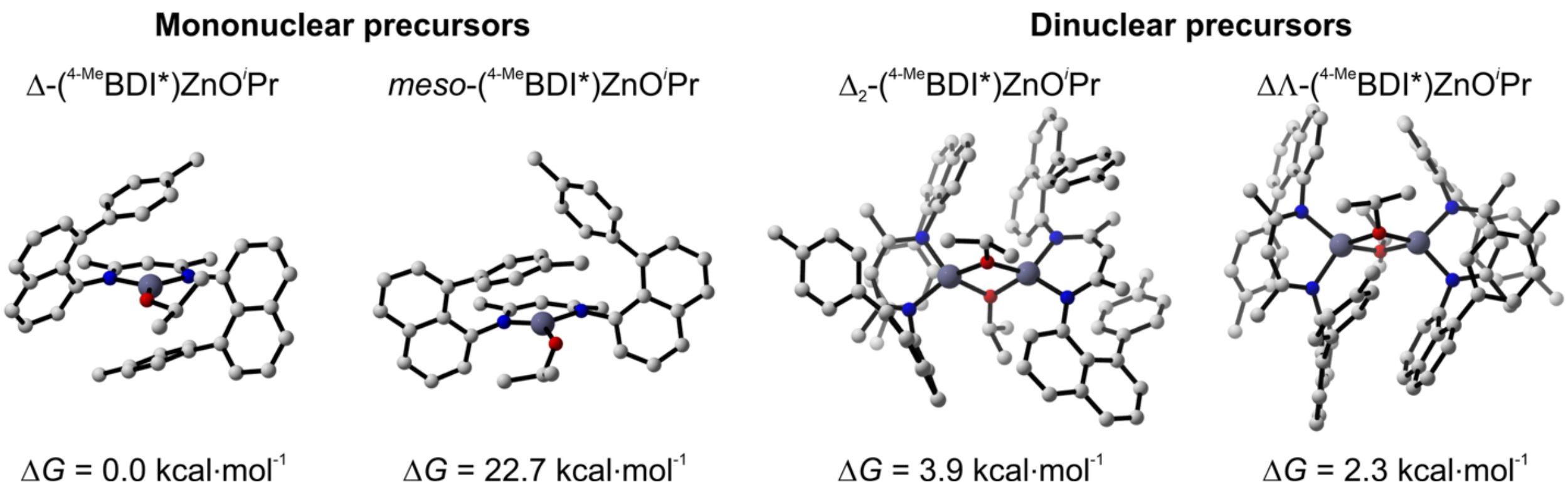

Figure S27: The DFT optimized structures and Gibbs energies of mononuclear (left) and dinuclear (right) precursors of ($^{4\text{-Me}}$BDI*)ZnO^{i}Pr. H atoms are omitted for clarity.

5.5 Elements of Chirality and Proposed Polymerization Mechanism

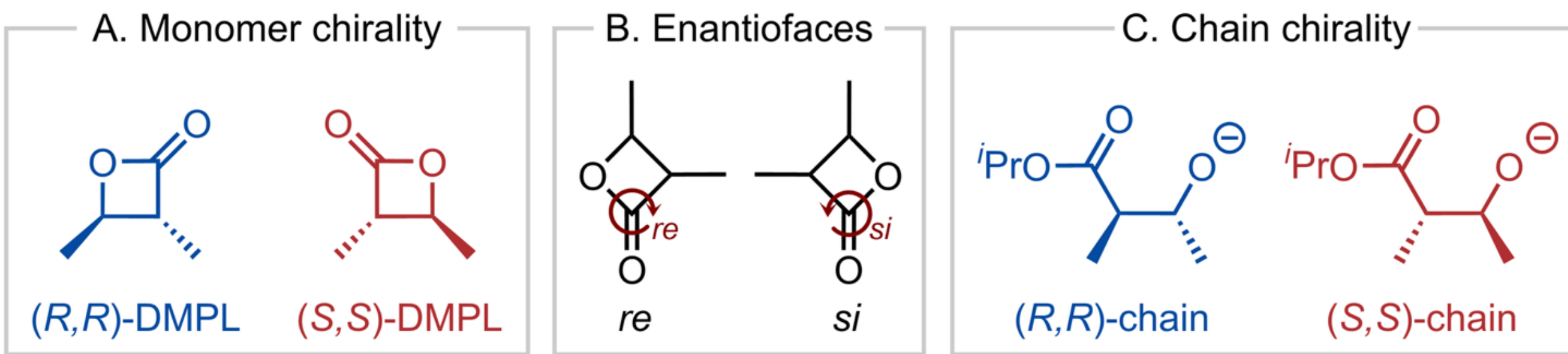

Scheme S2: Elements of chirality of the reaction: monomer enantiomers (A), monomer enantiofaces (B) and chirality of the growing chain (C).

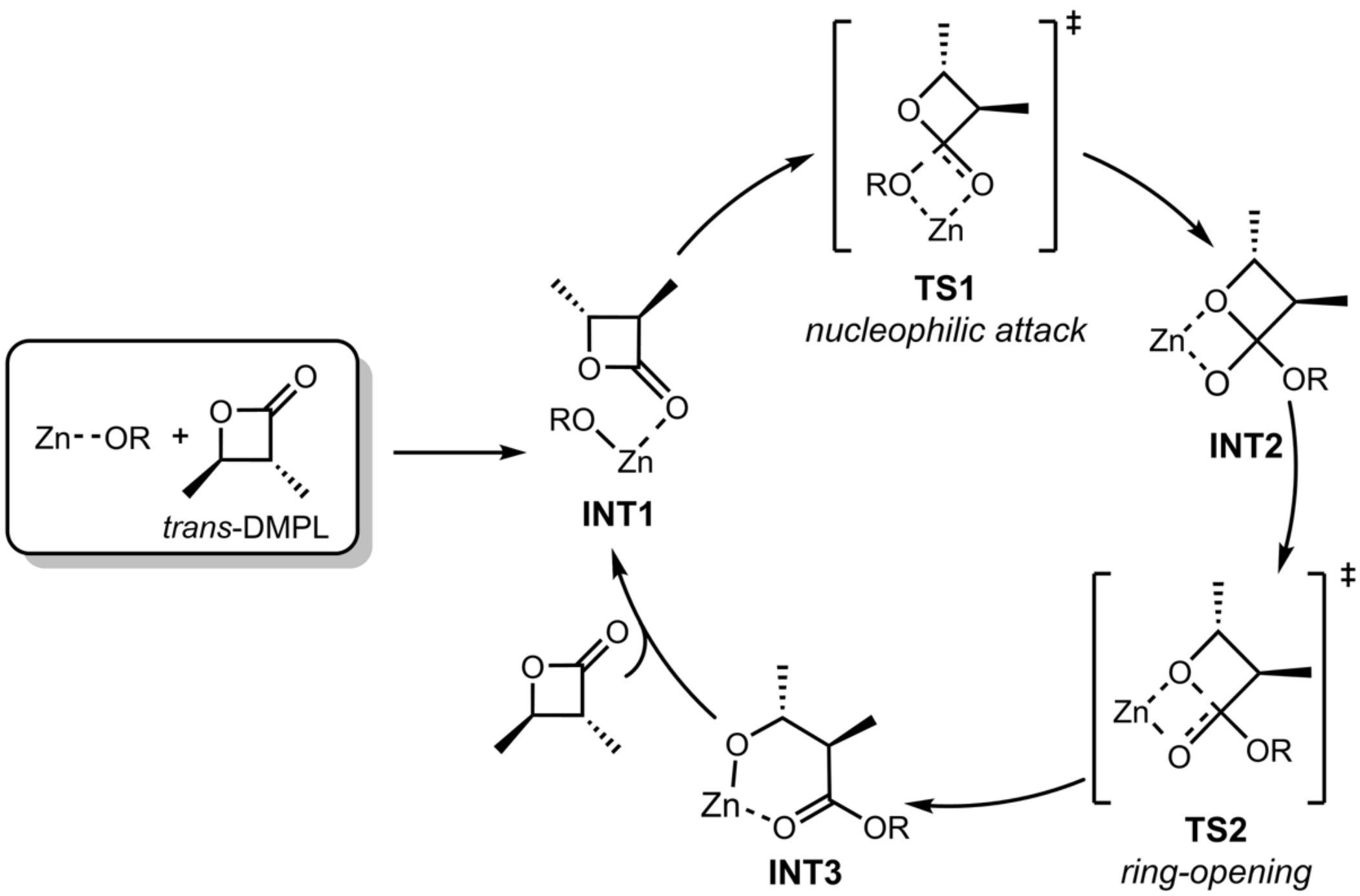

Scheme S3: Catalytic cycle proposed for the ring-opening polymerization of *trans*-DMPL promoted by *rac*-**1**.

5.6 Minimum Energy Pathway of Initiation

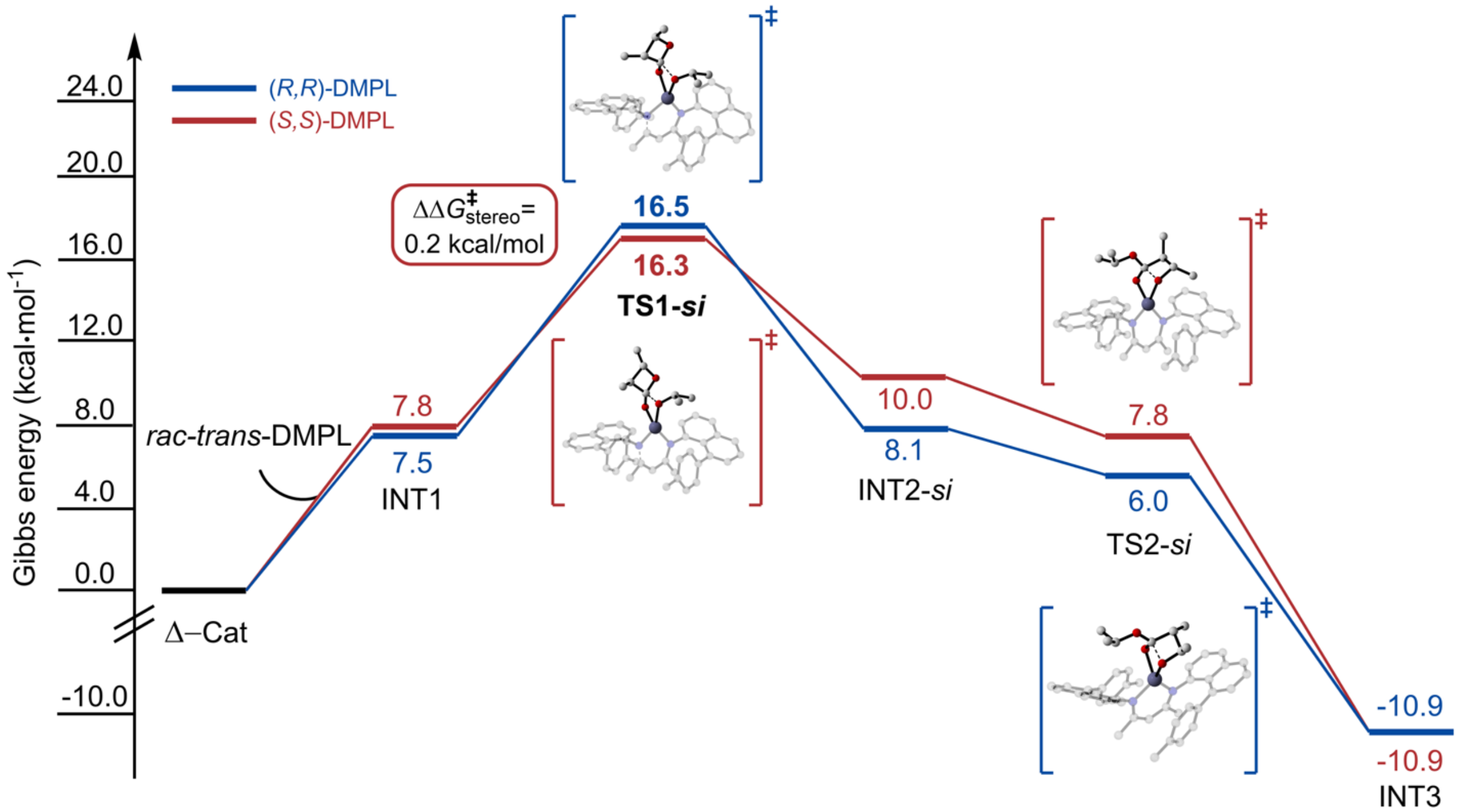

Figure S28: Minimum energy path for the initiation of the ROP of *rac-trans*-DMPL promoted by Δ-**1**. The ROP of (*R,R*)-DMPL is shown in blue, whereas that of (*S,S*)-DMPL is shown in red.

Table S7: Gibbs energies for the rate-determining step (TS1) of both enantiofaces for all Δ reaction pathways. The lowest-energy transition states are shown in bold.

RDS	Enantioface (kcal·mol-1)	
	$\Delta G^{\ddagger}_{re}$	$\Delta G^{\ddagger}_{si}$
$\mathrm{TS1}_{RR}$	18.2	**16.5**
$\mathrm{TS1}_{SS}$	18.3	**16.3**
$\mathrm{TS1}_{RR\rightarrow RR}$	25.9	**19.4**
$\mathrm{TS1}_{RR\rightarrow SS}$	24.4	**20.7**
$\mathrm{TS1}_{SS\rightarrow SS}$	**20.5**	21.8
$\mathrm{TS1}_{SS\rightarrow RR}$	**20.7**	24.7

Table S8: ASM results for enantioselectivity-determining TSs in the first insertion of the ROP of *rac-trans*-DMPL promoted by Δ-($^{4\text{-Me}}$BDI*)ZnO^{i}Pr.

		ΔE_{Strain} (kcal·mol^{-1})		
		Catalyst + Chain	Monomer	Total
Δ-($^{4\text{-Me}}$BDI*)ZnO^{i}Pr	TS1$_{RR}$	16.2	16.5	32.7
	TS1$_{SS}$	15.8	17.1	32.9
	$\Delta\Delta E$	-0.4	0.6	0.2

5.7 Chain Conformation Analysis

The conformations adopted by the (*R,R*)-chain, used to model the growing chains of PHMB, were carefully selected following an extensive conformational analysis performed on model compounds mimicking these polymers (**Scheme S4**). First, a 360° scan was performed about the θ_2 dihedral, from which 3 minima were identified: –160°, –60° and 50°. Fixing these minima, three different conformational energy maps were generated (**Figure S30**), scanning the other two key dihedrals: θ_1 and θ_3. All the conformational minima identified are reported in **Table S9**.

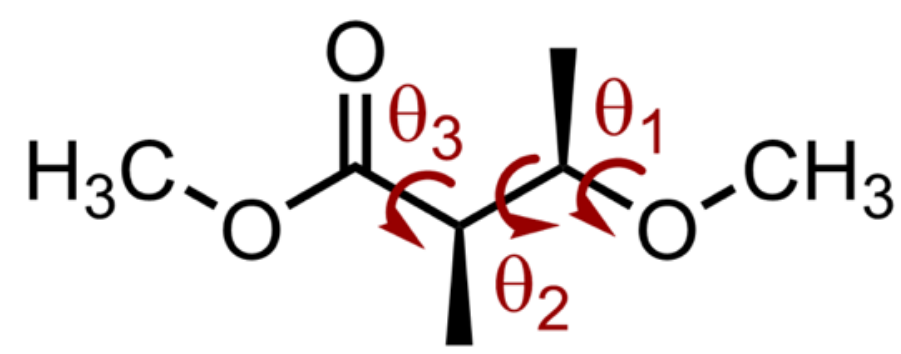

Scheme S4: Relevant dihedrals of single-unit propagating chain of (*R,R*)-PHMB.

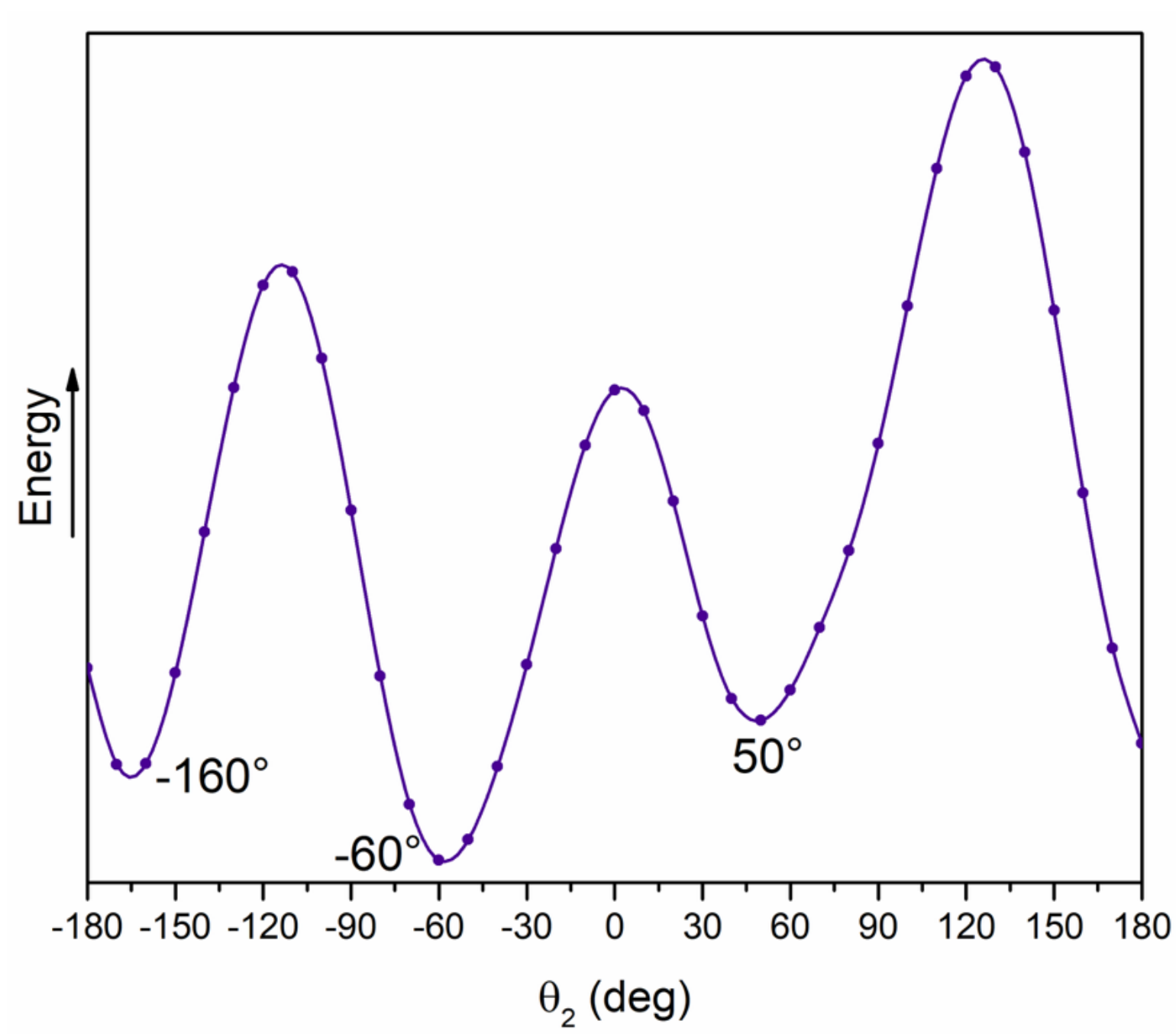

Figure S29: Potential energy scan of θ_2 indicating three minima at –160°, –60° and 50°.

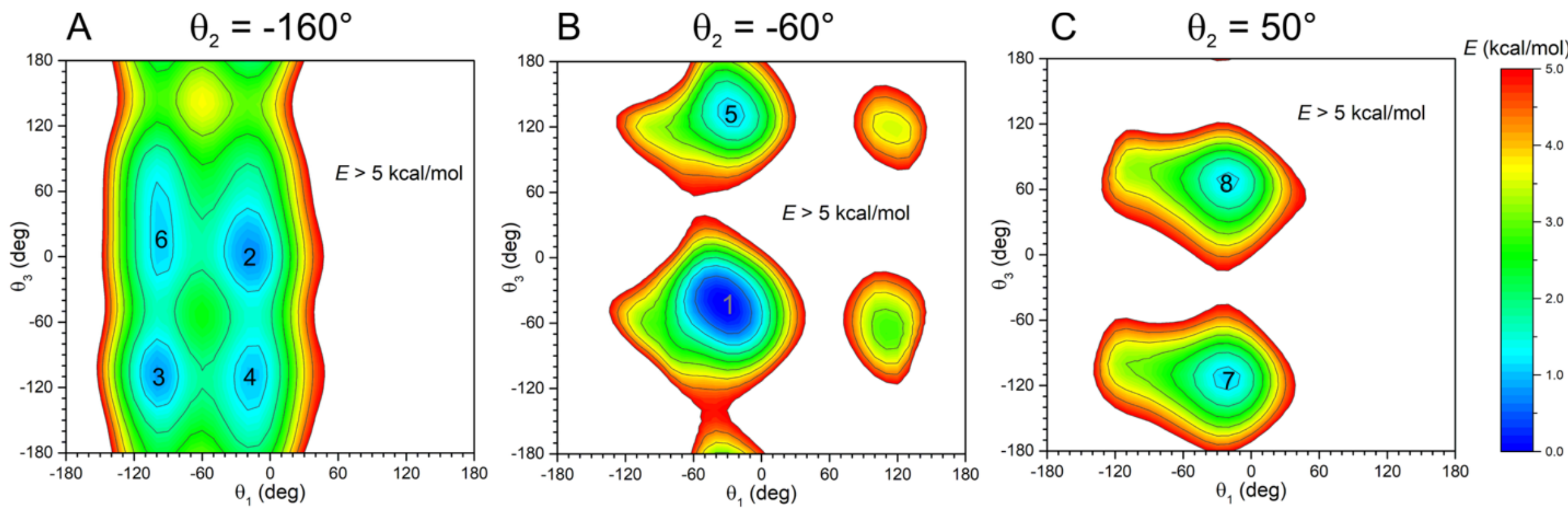

Figure S30: Maps of the conformational energy of (*R,R*) chain as a function of the torsion angles θ_1 and θ_3, at fixed θ_2 values of -160° (A), -60° (B) and 50° (C). The absolute energy minimum is placed at E = 0 kcal·mol^{-1}and the energy values are scaled with respect to the absolute minimum. The colors indicate the scale of energy that decreases from red to blue.

Table S9: Conformational minima of single-unit propagating chain of (*R,R*)-PHMB.

**Minimum**	**θ_1 (deg)**	**θ_2 (deg)**	**θ_3 (deg)**	**ΔΔ*E* (kcal·mol^{-1})**
1	-30	-60	-40	0.0
2	-20	-160	0	0.7
3	-100	-160	-110	0.8
4	-20	-160	-110	1.0
5	-30	-60	130	1.1
6	-100	-160	20	1.1
7	-20	50	-110	1.1
8	-20	50	70	1.1

It is worth noting that the calculated free energy difference between the propagating intermediates (INT3) leading to isotactic and syndiotactic enchainments for Δ-RR is approximately 2 kcal·mol^{-1}, with the isotactic intermediate being lower in energy. These intermediates possess considerable conformational flexibility owing to the growing polymer chain composed of two monomer units, and the reported structures represent only one of many accessible, low-energy conformations on a very flat conformational landscape.

Furthermore, any stabilization of the isotactic propagating intermediate would be expected, according to the Hammond postulate, to be reflected in the corresponding transition state for the subsequent monomer insertion. Therefore, such an energetic difference would not be expected to diminish the computed stereoselectivity, but rather to be carried forward into the next propagation event.

5.8 Activation Strain Model (ASM) Analysis

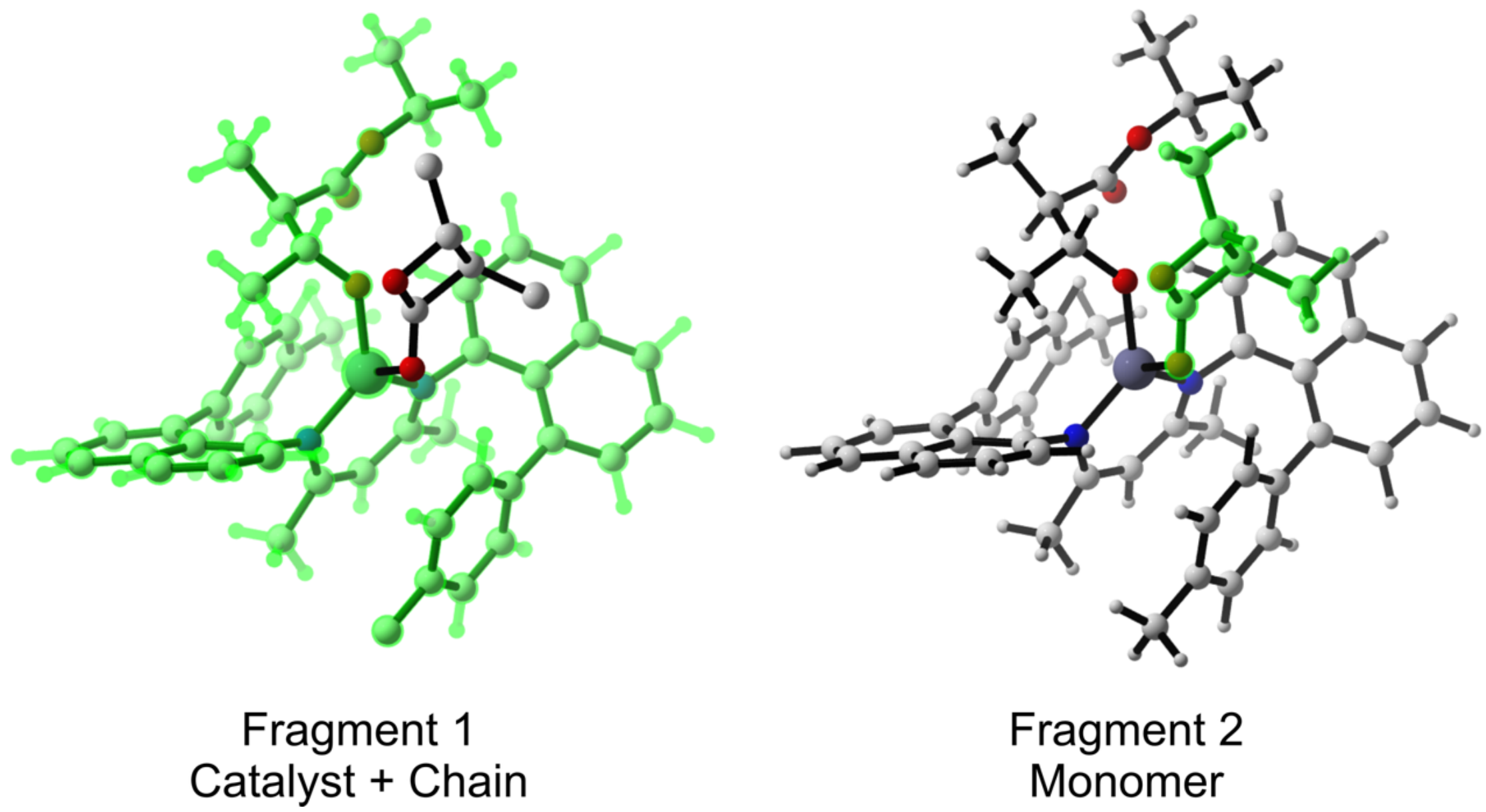

Figure S31: Definition of fragments for ASM analysis.

5.9 TS1 Structures for INT3$_{SS}$-Δ (Δ-*SS*)

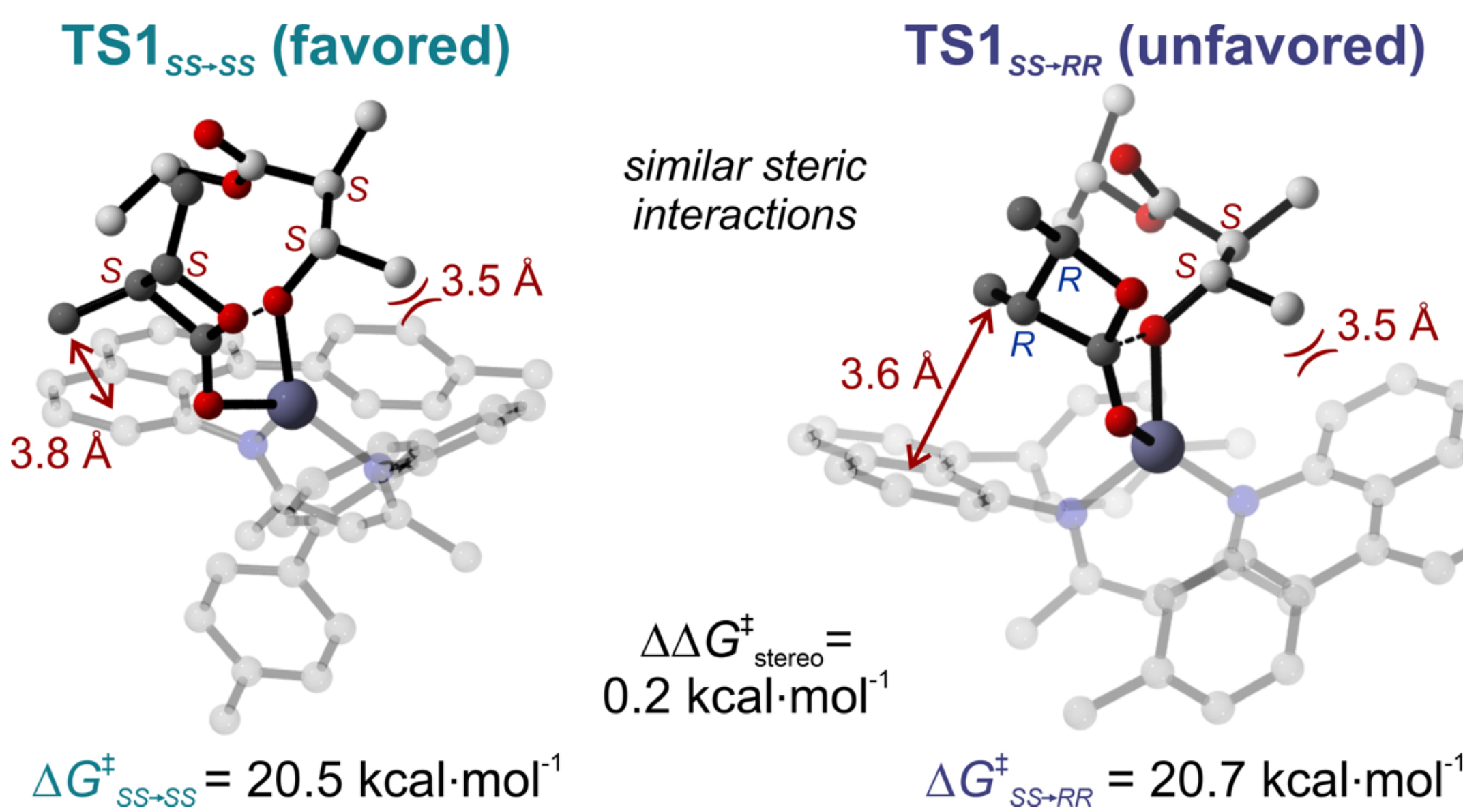

Figure S32: Optimized structures of stereoselectivity determining step (TS1) in the ROP of *trans*-DMPL promoted by INT3$_{SS}$-Δ (Δ-*SS*).

5.10 Buried Volume (%V_{Bur}) Analysis

Table S10: Occupation values of octants and quadrants obtained from %V_{Bur} analysis.

Octant	SW-z	NW-z	NE-z	SE-z	SW+z	NW+z	NE+z	SE+z
%V_{Bur}	93.3	92.7	92.7	92.9	45.1	70.5	42.9	66.5

Quadrant	SW	NW	NE	SE
%V_{Bur}	69.2	81.6	67.8	79.7

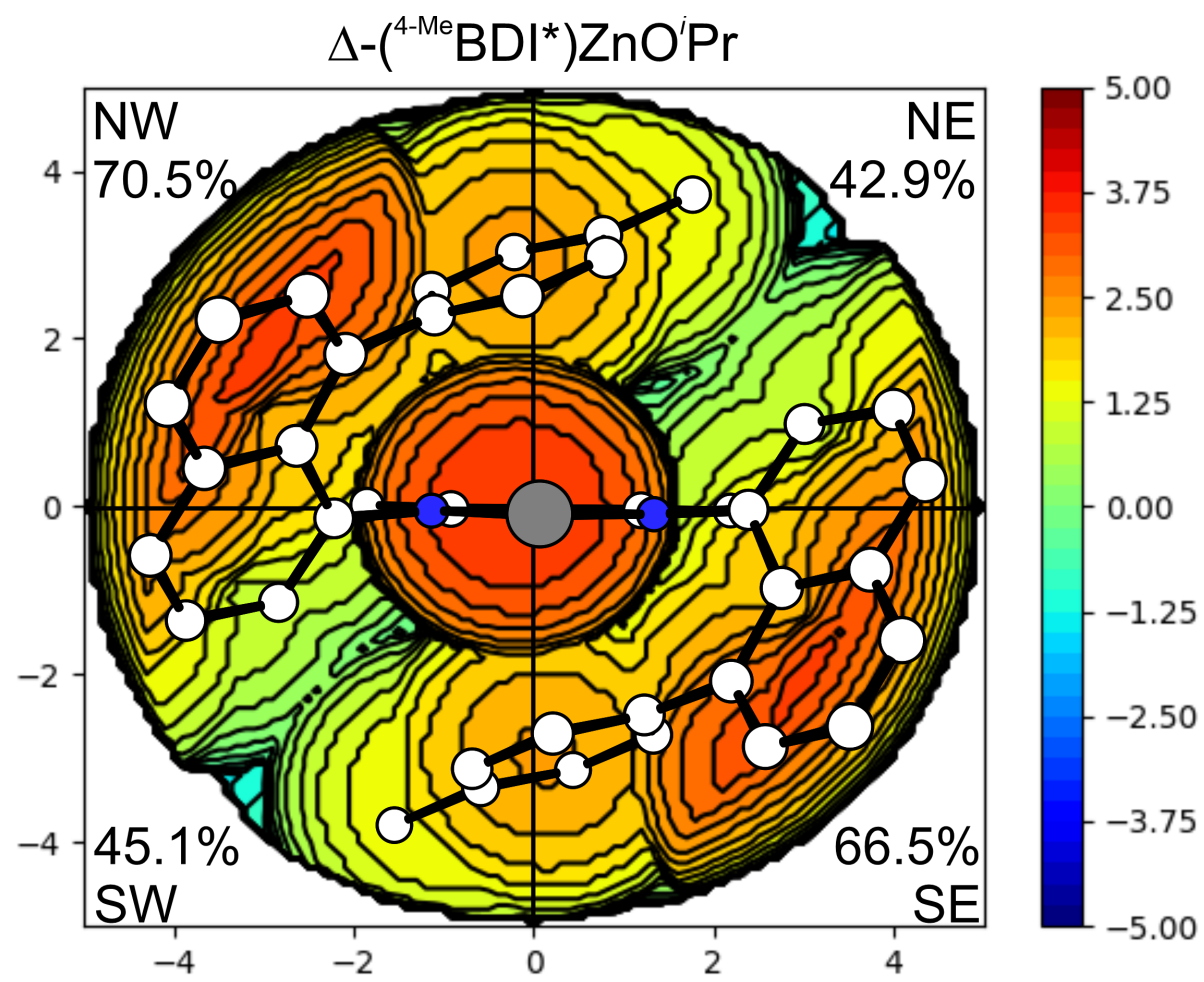

Figure S33: Steric maps obtained *via* %V_{Bur} octant analysis of the catalytic pocket created by Δ-**1**.

6 X-Ray Crystallographic Data

6.1 *rac*-($^{4\text{-Me}}$BDI*)ZnO^{i}Pr (*rac*-1)

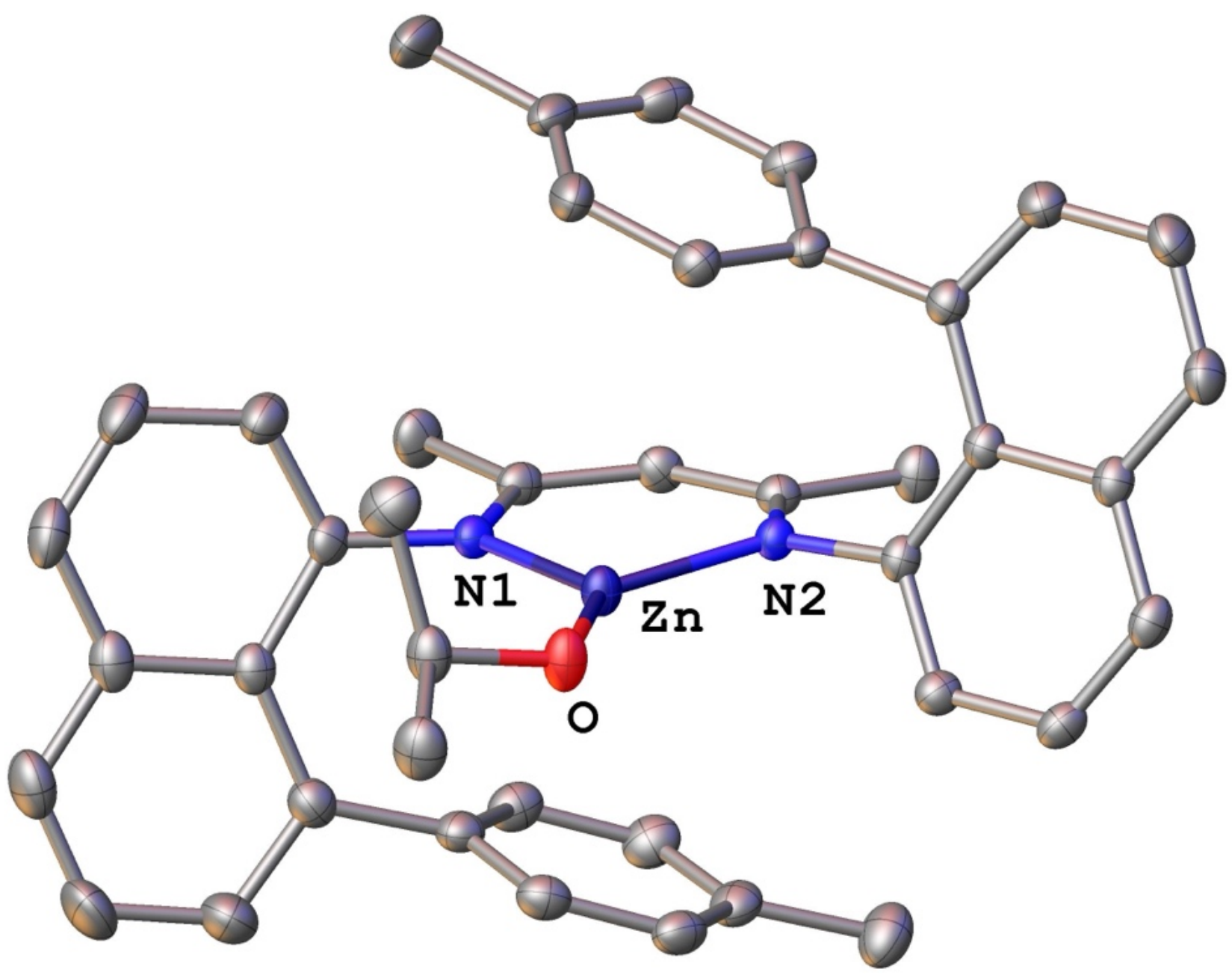

Figure S34: ORTEP drawing of *rac*-($^{4\text{-Me}}$BDI*)ZnO^{i}Pr (*rac*-**1**) with thermal ellipsoids drawn at the 50% probability level. Crystal grown from a concentrated solution in toluene layered with hexanes. CCDC2563348.

Table S11: Crystal data and structure refinement for Rmsy3 (*rac*-**1**). CCDC2563348.

Identification code	rmsy3_abs	
Empirical formula	C42 H40 N2 O Zn	
Formula weight	654.13	
Temperature	100.02(10) K	
Wavelength	1.54184 Å	
Crystal system	Monoclinic	
Space group	P 1 21/n 1	
Unit cell dimensions	a = 9.97740(10) Å	α = 90°
	b = 23.2330(2) Å	β = 98.2400(10)°
	c = 15.0241(2)Å	γ = 90°
Volume	3446.71(7) $Å^3$	
Z	4	
Density (calculated)	1.261 Mg/m^3	
Absorption coefficient	1.247 mm^{-1}	
F(000)	1376	
Crystal size	0.27 x 0.079 x 0.072 mm	
Theta range for data collection	3.529 to 80.006°	
Index ranges	-12 ≤ h ≤ 12, -29 ≤ k ≤ 29, -19 ≤ l ≤ 19	
Reflections collected	51483	
Independent reflections	7501 [R_{int} = 0.0338]	
Completeness to theta = 67.684∞	100.0 %	
Absorption correction	Gaussian	
Max. and min. transmission	1.000 and 0.522	
Refinement method	Full-matrix least-squares on F^2	
Data / restraints / parameters	7501 / 0 / 421	
Goodness-of-fit on F^2	1.069	
Final R indices [I>2sigma(I)]	R1 = 0.0321, wR2 = 0.0860	
R indices (all data)	R1 = 0.0347, wR2 = 0.0880	
Extinction coefficient	n/a	
Largest diff. peak and hole	0.322 and -0.421 e $Å^{-3}$	

Table S12: Atomic coordinates (x 10^4) and equivalent isotropic displacement parameters ($Å^2$ x 10^3) for Rmsy3. U_{eq} is defined as one third of the trace of the orthogonalized U^{ij} tensor.

	x	y	z	U_{eq}
Zn	7152(1)	3800(1)	7103(1)	20(1)
O	5451(1)	3949(1)	7365(1)	27(1)
N(1)	8497(1)	3258(1)	7666(1)	19(1)
N(2)	8063(1)	4089(1)	6143(1)	18(1)
C(1)	9978(2)	5610(1)	7490(1)	38(1)
C(2)	9584(2)	5111(1)	8043(1)	27(1)
C(3)	8293(2)	5072(1)	8289(1)	27(1)
C(4)	7914(2)	4600(1)	8760(1)	26(1)
C(5)	8818(1)	4148(1)	9003(1)	23(1)
C(6)	10124(2)	4197(1)	8784(1)	27(1)
C(7)	10495(2)	4669(1)	8309(1)	30(1)
C(8)	8393(2)	3664(1)	9556(1)	26(1)
C(9)	8256(2)	3805(1)	10430(1)	38(1)
C(10)	7806(2)	3405(1)	11025(1)	45(1)
C(11)	7482(2)	2862(1)	10740(1)	37(1)
C(12)	7612(1)	2686(1)	9854(1)	27(1)
C(13)	7265(2)	2118(1)	9580(1)	33(1)
C(14)	7351(2)	1940(1)	8729(1)	35(1)
C(15)	7790(2)	2328(1)	8112(1)	29(1)
C(16)	8151(1)	2883(1)	8349(1)	21(1)
C(17)	8078(1)	3088(1)	9241(1)	22(1)
C(18)	10678(2)	2759(1)	7786(1)	29(1)
C(19)	9668(1)	3197(1)	7354(1)	20(1)
C(20)	10061(1)	3526(1)	6655(1)	21(1)
C(21)	9338(1)	3940(1)	6097(1)	19(1)
C(22)	10096(1)	4233(1)	5426(1)	25(1)
C(23)	7393(1)	4506(1)	5531(1)	18(1)
C(24)	7141(1)	5036(1)	5878(1)	22(1)
C(25)	6529(1)	5484(1)	5335(1)	23(1)
C(26)	6204(1)	5402(1)	4430(1)	22(1)
C(27)	6398(1)	4855(1)	4049(1)	21(1)

C(28)	5996(2)	4778(1)	3113(1)	26(1)
C(29)	6132(2)	4255(1)	2724(1)	31(1)
C(30)	6624(2)	3783(1)	3266(1)	28(1)
C(31)	6989(1)	3829(1)	4182(1)	21(1)
C(32)	6947(1)	4384(1)	4600(1)	18(1)
C(33)	7278(1)	3271(1)	4672(1)	21(1)
C(34)	6330(2)	3036(1)	5169(1)	25(1)
C(35)	6499(2)	2485(1)	5524(1)	30(1)
C(36)	7624(2)	2153(1)	5408(1)	31(1)
C(37)	8575(2)	2390(1)	4922(1)	30(1)
C(38)	8397(2)	2938(1)	4548(1)	26(1)
C(39)	7798(2)	1550(1)	5791(1)	45(1)
C(40)	4864(1)	3573(1)	7939(1)	26(1)
C(41)	3639(2)	3867(1)	8230(1)	33(1)
C(42)	4479(2)	3007(1)	7467(1)	36(1)

Table S13: Bond lengths [Å] and angles [°] for Rmsy3.

Zn-O	1.8304(10)	C(24)-C(25)	1.4073(18)
Zn-N(1)	1.9425(11)	C(25)-C(26)	1.364(2)
Zn-N(2)	1.9316(11)	C(26)-C(27)	1.4175(19)
O-C(40)	1.4122(17)	C(27)-C(28)	1.4173(19)
N(1)-C(16)	1.4246(16)	C(27)-C(32)	1.4349(17)
N(1)-C(19)	1.3277(17)	C(28)-C(29)	1.364(2)
N(2)-C(21)	1.3305(17)	C(29)-C(30)	1.411(2)
N(2)-C(23)	1.4338(15)	C(30)-C(31)	1.376(2)
C(1)-C(2)	1.510(2)	C(31)-C(32)	1.4377(18)
C(2)-C(3)	1.393(2)	C(31)-C(33)	1.4982(18)
C(2)-C(7)	1.391(2)	C(33)-C(34)	1.3971(19)
C(3)-C(4)	1.386(2)	C(33)-C(38)	1.3915(19)
C(4)-C(5)	1.397(2)	C(34)-C(35)	1.389(2)
C(5)-C(6)	1.394(2)	C(35)-C(36)	1.393(2)
C(5)-C(8)	1.497(2)	C(36)-C(37)	1.392(2)
C(6)-C(7)	1.388(2)	C(36)-C(39)	1.516(2)
C(8)-C(9)	1.379(2)	C(37)-C(38)	1.393(2)
C(8)-C(17)	1.437(2)	C(40)-C(41)	1.517(2)
C(9)-C(10)	1.407(3)	C(40)-C(42)	1.516(2)
C(10)-C(11)	1.357(3)		
C(11)-C(12)	1.416(2)	O-Zn-N(1)	129.27(5)
C(12)-C(13)	1.411(2)	O-Zn-N(2)	130.14(4)
C(12)-C(17)	1.4353(19)	N(2)-Zn-N(1)	100.46(5)
C(13)-C(14)	1.358(3)	C(40)-O-Zn	119.53(8)
C(14)-C(15)	1.408(2)	C(16)-N(1)-Zn	119.09(8)
C(15)-C(16)	1.374(2)	C(19)-N(1)-Zn	120.25(9)
C(16)-C(17)	1.4348(19)	C(19)-N(1)-C(16)	120.29(11)
C(18)-C(19)	1.5114(17)	C(21)-N(2)-Zn	120.26(9)
C(19)-C(20)	1.3994(19)	C(21)-N(2)-C(23)	120.27(11)
C(20)-C(21)	1.4060(18)	C(23)-N(2)-Zn	119.24(8)
C(21)-C(22)	1.5073(18)	C(3)-C(2)-C(1)	121.32(14)
C(23)-C(24)	1.3736(18)	C(7)-C(2)-C(1)	120.89(14)
C(23)-C(32)	1.4337(18)	C(7)-C(2)-C(3)	117.78(14)

C(4)-C(3)-C(2)	121.17(13)	C(24)-C(23)-N(2)	117.10(11)
C(3)-C(4)-C(5)	120.95(14)	C(24)-C(23)-C(32)	120.00(11)
C(4)-C(5)-C(8)	119.14(13)	C(32)-C(23)-N(2)	122.86(11)
C(6)-C(5)-C(4)	117.86(13)	C(23)-C(24)-C(25)	122.05(12)
C(6)-C(5)-C(8)	122.75(13)	C(26)-C(25)-C(24)	119.68(12)
C(7)-C(6)-C(5)	120.86(13)	C(25)-C(26)-C(27)	120.12(12)
C(6)-C(7)-C(2)	121.31(14)	C(26)-C(27)-C(32)	120.88(12)
C(9)-C(8)-C(5)	115.09(13)	C(28)-C(27)-C(26)	118.63(12)
C(9)-C(8)-C(17)	119.23(14)	C(28)-C(27)-C(32)	120.46(12)
C(17)-C(8)-C(5)	125.63(12)	C(29)-C(28)-C(27)	120.43(13)
C(8)-C(9)-C(10)	122.11(16)	C(28)-C(29)-C(30)	119.52(13)
C(11)-C(10)-C(9)	119.87(16)	C(31)-C(30)-C(29)	122.51(13)
C(10)-C(11)-C(12)	120.93(15)	C(30)-C(31)-C(32)	119.12(12)
C(11)-C(12)-C(17)	119.86(14)	C(30)-C(31)-C(33)	115.43(12)
C(13)-C(12)-C(11)	119.46(14)	C(32)-C(31)-C(33)	125.19(12)
C(13)-C(12)-C(17)	120.67(14)	C(23)-C(32)-C(27)	116.81(11)
C(14)-C(13)-C(12)	120.93(14)	C(23)-C(32)-C(31)	125.49(11)
C(13)-C(14)-C(15)	119.53(14)	C(27)-C(32)-C(31)	117.70(12)
C(16)-C(15)-C(14)	121.71(15)	C(34)-C(33)-C(31)	120.15(12)
N(1)-C(16)-C(17)	121.32(12)	C(38)-C(33)-C(31)	121.13(12)
C(15)-C(16)-N(1)	117.99(12)	C(38)-C(33)-C(34)	118.17(13)
C(15)-C(16)-C(17)	120.52(13)	C(35)-C(34)-C(33)	120.72(14)
C(12)-C(17)-C(8)	117.99(13)	C(34)-C(35)-C(36)	121.21(14)
C(16)-C(17)-C(8)	125.35(12)	C(35)-C(36)-C(39)	121.02(16)
C(16)-C(17)-C(12)	116.63(13)	C(37)-C(36)-C(35)	117.99(13)
N(1)-C(19)-C(18)	119.17(12)	C(37)-C(36)-C(39)	120.98(16)
N(1)-C(19)-C(20)	124.16(12)	C(36)-C(37)-C(38)	121.04(14)
C(20)-C(19)-C(18)	116.65(12)	C(33)-C(38)-C(37)	120.85(14)
C(19)-C(20)-C(21)	130.29(12)	O-C(40)-C(41)	108.59(12)
N(2)-C(21)-C(20)	124.05(12)	O-C(40)-C(42)	110.50(12)
N(2)-C(21)-C(22)	119.50(11)	C(42)-C(40)-C(41)	111.34(13)
C(20)-C(21)-C(22)	116.44(11)		

Table S14: Anisotropic displacement parameters ($Å^2 \times 10^3$) for Rmsy3. The anisotropic displacement factor exponent takes the form: $-2\pi^2[h^2 a^{*2}U^{11} + ... + 2 h k a^* b^* U^{12}]$

	U^{11}	U^{22}	U^{33}	U^{23}	U^{13}	U^{12}
Zn	17(1)	22(1)	22(1)	5(1)	3(1)	3(1)
O	20(1)	30(1)	31(1)	12(1)	7(1)	5(1)
N(1)	21(1)	18(1)	17(1)	2(1)	0(1)	2(1)
N(2)	18(1)	17(1)	18(1)	2(1)	2(1)	1(1)
C(1)	38(1)	31(1)	42(1)	6(1)	-1(1)	-8(1)
C(2)	31(1)	22(1)	27(1)	-3(1)	-1(1)	-5(1)
C(3)	31(1)	22(1)	27(1)	-4(1)	-1(1)	4(1)
C(4)	25(1)	27(1)	25(1)	-5(1)	3(1)	2(1)
C(5)	27(1)	21(1)	21(1)	-4(1)	1(1)	-1(1)
C(6)	24(1)	24(1)	31(1)	-2(1)	0(1)	2(1)
C(7)	24(1)	28(1)	36(1)	-2(1)	3(1)	-3(1)
C(8)	27(1)	27(1)	22(1)	0(1)	4(1)	2(1)
C(9)	54(1)	35(1)	28(1)	-5(1)	11(1)	-1(1)
C(10)	65(1)	50(1)	24(1)	1(1)	18(1)	4(1)
C(11)	38(1)	45(1)	30(1)	11(1)	12(1)	3(1)
C(12)	22(1)	32(1)	27(1)	9(1)	4(1)	2(1)
C(13)	30(1)	32(1)	37(1)	16(1)	2(1)	-4(1)
C(14)	42(1)	23(1)	38(1)	7(1)	-4(1)	-7(1)
C(15)	37(1)	22(1)	27(1)	2(1)	-2(1)	-1(1)
C(16)	19(1)	20(1)	22(1)	5(1)	-1(1)	2(1)
C(17)	18(1)	24(1)	22(1)	5(1)	2(1)	2(1)
C(18)	27(1)	30(1)	29(1)	7(1)	2(1)	11(1)
C(19)	20(1)	19(1)	20(1)	-1(1)	-1(1)	4(1)
C(20)	18(1)	23(1)	22(1)	1(1)	3(1)	4(1)
C(21)	18(1)	18(1)	19(1)	-1(1)	2(1)	0(1)
C(22)	20(1)	27(1)	30(1)	7(1)	6(1)	3(1)
C(23)	15(1)	18(1)	21(1)	3(1)	2(1)	0(1)
C(24)	21(1)	21(1)	22(1)	-1(1)	1(1)	0(1)
C(25)	21(1)	18(1)	30(1)	0(1)	2(1)	1(1)
C(26)	17(1)	20(1)	29(1)	6(1)	2(1)	1(1)

C(27)	18(1)	21(1)	23(1)	4(1)	2(1)	-1(1)
C(28)	28(1)	27(1)	23(1)	7(1)	1(1)	0(1)
C(29)	41(1)	32(1)	19(1)	2(1)	0(1)	-3(1)
C(30)	36(1)	23(1)	24(1)	-1(1)	4(1)	-3(1)
C(31)	22(1)	20(1)	22(1)	1(1)	4(1)	-1(1)
C(32)	16(1)	18(1)	21(1)	2(1)	3(1)	0(1)
C(33)	25(1)	18(1)	20(1)	-2(1)	1(1)	-2(1)
C(34)	28(1)	22(1)	26(1)	0(1)	5(1)	-1(1)
C(35)	43(1)	22(1)	24(1)	2(1)	6(1)	-6(1)
C(36)	48(1)	18(1)	24(1)	-2(1)	-3(1)	1(1)
C(37)	32(1)	23(1)	33(1)	-6(1)	-2(1)	5(1)
C(38)	26(1)	22(1)	30(1)	-4(1)	5(1)	0(1)
C(39)	80(1)	21(1)	33(1)	2(1)	1(1)	6(1)
C(40)	21(1)	30(1)	28(1)	7(1)	7(1)	2(1)
C(41)	27(1)	36(1)	38(1)	6(1)	12(1)	5(1)
C(42)	30(1)	33(1)	46(1)	0(1)	11(1)	-2(1)

Table S15: Hydrogen coordinates (x 10^4) and isotropic displacement parameters (Å^2 x 10^3) for Rmsy3.

	x	y	z	U_{eq}
H(1A)	9522	5572	6870	56
H(1B)	10961	5609	7493	56
H(1C)	9706	5972	7746	56
H(3)	7661	5373	8130	33
H(4)	7028	4583	8919	31
H(6)	10769	3904	8963	32
H(7)	11388	4691	8161	35
H(9)	8473	4185	10637	46
H(10)	7729	3515	11624	54
H(11)	7163	2595	11141	44
H(13)	6967	1857	9997	40
H(14)	7117	1556	8552	42
H(15)	7837	2202	7515	35
H(18A)	10706	2770	8441	43
H(18B)	11578	2848	7634	43
H(18C)	10406	2373	7564	43
H(20)	10959	3458	6540	25
H(22A)	9674	4136	4815	38
H(22B)	11040	4104	5516	38
H(22C)	10065	4651	5510	38
H(24)	7387	5102	6504	26
H(25)	6344	5842	5596	28
H(26)	5848	5711	4056	27
H(28)	5629	5093	2754	31
H(29)	5899	4209	2092	37
H(30)	6706	3420	2988	33
H(34)	5562	3257	5265	30
H(35)	5834	2331	5851	35
H(37)	9358	2174	4842	36
H(38)	9047	3087	4205	31

H(39A)	8503	1551	6318	68
H(39B)	8063	1289	5334	68
H(39C)	6942	1419	5970	68
H(40)	5539	3495	8484	31
H(41A)	3925	4221	8557	50
H(41B)	3213	3608	8621	50
H(41C)	2987	3961	7698	50
H(42A)	3823	3079	6928	53
H(42B)	4074	2751	7874	53
H(42C)	5290	2826	7294	53

6.2 *rac*-($^{4\text{-Me}}$BDI*)Zn(N(SiMe$_3$)$_2$

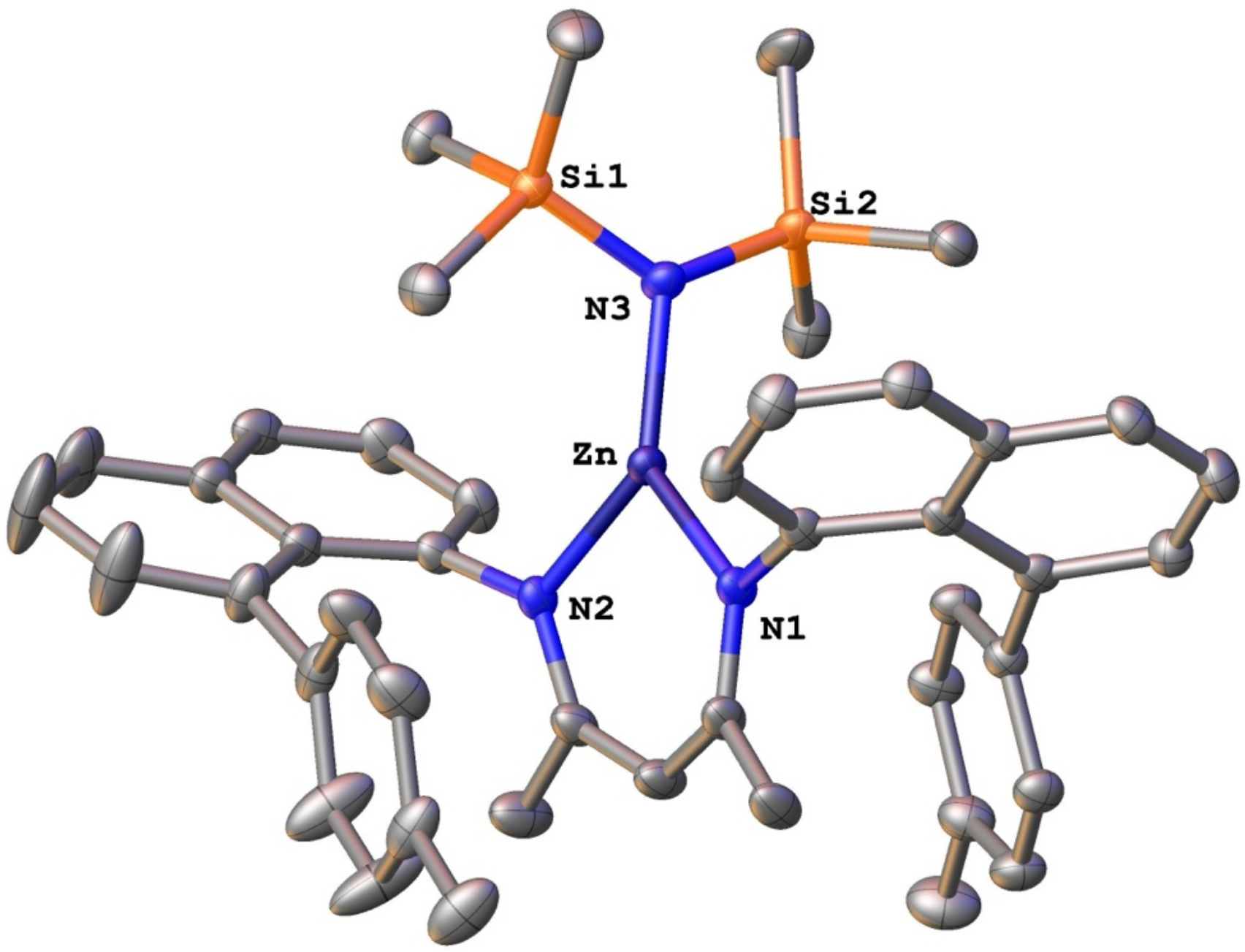

Figure S35: ORTEP structure of *rac*-($^{4\text{-Me}}$BDI*)Zn(N(SiMe$_3$)$_2$) with thermal ellipsoids drawn at the 50% probability level. CCDC2563347.

Table S16: Crystal data and structure refinement for Rmsy2 (*rac*-($^{4\text{-Me}}$BDI*)Zn(N(SiMe$_3$)$_2$)). CCDC2563347.

Identification code	rmsy2_abs	
Empirical formula	C45 H51 N3 Si2 Zn	
Formula weight	755.43	
Temperature	100.15 K	
Wavelength	1.54184 Å	
Crystal system	Triclinic	
Space group	P -1	
Unit cell dimensions	a = 10.12100(10) Å	α = 97.4260(10)°
	b = 11.70640(10) Å	β = 95.6000(10)°
	c = 18.7685(2) Å	γ = 114.6530(10)°
Volume	1975.54(4) Å^3	
Z	2	
Density (calculated)	1.270 Mg/m^3	
Absorption coefficient	1.704 mm^{-1}	
F(000)	800	
Crystal size	0.217 x 0.196 x 0.116 mm	
Theta range for data collection	2.408 to 80.018°.	
Index ranges	-12 ≤ h ≤ 12, -14 ≤ k ≤ 10, -23 ≤ l ≤ 23	
Reflections collected	45072	
Independent reflections	8520 [R_{int} = 0.0435]	
Completeness to theta = 67.684∞	100.0 %	
Absorption correction	Gaussian	
Max. and min. transmission	1.000 and 0.423	
Refinement method	Full-matrix least-squares on F^2	
Data / restraints / parameters	8520 / 0 / 470	
Goodness-of-fit on F^2	1.101	
Final R indices [I>2sigma(I)]	R1 = 0.0348, wR2 = 0.0910	
R indices (all data)	R1 = 0.0383, wR2 = 0.0946	
Extinction coefficient	n/a	
Largest diff. peak and hole	0.480 and -0.444 e Å^{-3}	

Table S17: Atomic coordinates (x 10^4) and equivalent isotropic displacement parameters (Å^2 x 10^3) for Rmsy2. U_{eq} is defined as one third of the trace of the orthogonalized U^{ij} tensor.

	x	y	z	U_{eq}
Zn	4231(1)	5790(1)	7466(1)	17(1)
Si(1)	7176(1)	8119(1)	8049(1)	19(1)
Si(2)	6424(1)	6958(1)	6428(1)	21(1)
N(1)	3368(2)	3949(1)	7474(1)	20(1)
N(2)	2464(2)	6024(1)	7613(1)	19(1)
N(3)	6096(2)	6979(1)	7307(1)	21(1)
C(1)	-1301(2)	2831(2)	5035(1)	41(1)
C(2)	13(2)	2658(2)	5363(1)	28(1)
C(3)	-138(2)	1496(2)	5532(1)	28(1)
C(4)	1077(2)	1344(2)	5837(1)	24(1)
C(5)	2479(2)	2360(2)	5994(1)	21(1)
C(6)	2637(2)	3526(2)	5815(1)	23(1)
C(7)	1425(2)	3670(2)	5509(1)	27(1)
C(8)	3781(2)	2143(1)	6277(1)	20(1)
C(9)	4146(2)	1389(2)	5781(1)	24(1)
C(10)	5342(2)	1097(2)	5950(1)	27(1)
C(11)	6163(2)	1542(2)	6629(1)	25(1)
C(12)	5828(2)	2295(1)	7172(1)	21(1)
C(13)	6677(2)	2696(2)	7878(1)	24(1)
C(14)	6387(2)	3419(2)	8419(1)	28(1)
C(15)	5242(2)	3773(2)	8260(1)	25(1)
C(16)	4389(2)	3411(1)	7582(1)	20(1)
C(17)	4631(2)	2628(1)	7000(1)	18(1)
C(18)	1383(2)	1919(2)	7616(1)	30(1)
C(19)	1938(2)	3271(1)	7478(1)	24(1)
C(20)	903(2)	3761(2)	7377(1)	29(1)
C(21)	1136(2)	5035(2)	7432(1)	24(1)
C(22)	-201(2)	5286(2)	7288(1)	37(1)
C(23)	2607(2)	7304(1)	7752(1)	21(1)
C(24)	2735(2)	7928(2)	7176(1)	28(1)

C(25)	3035(2)	9235(2)	7267(1)	30(1)
C(26)	3235(2)	9901(2)	7949(1)	27(1)
C(27)	3095(2)	9299(2)	8560(1)	26(1)
C(28)	3321(3)	10032(2)	9256(1)	43(1)
C(29)	3236(4)	9504(2)	9859(1)	68(1)
C(30)	2878(4)	8194(2)	9787(1)	59(1)
C(31)	2601(2)	7407(2)	9125(1)	31(1)
C(32)	2746(2)	7956(1)	8476(1)	22(1)
C(33)	2137(2)	6023(2)	9174(1)	29(1)
C(34)	3143(2)	5610(2)	9454(1)	38(1)
C(35)	2694(2)	4373(2)	9591(1)	38(1)
C(36)	1217(2)	3514(2)	9463(1)	32(1)
C(37)	219(3)	3940(2)	9201(2)	57(1)
C(38)	666(3)	5172(2)	9057(2)	53(1)
C(39)	721(3)	2178(2)	9623(1)	42(1)
C(40)	6387(2)	7599(2)	8876(1)	29(1)
C(41)	7178(2)	9712(2)	8007(1)	29(1)
C(42)	9139(2)	8354(2)	8211(1)	33(1)
C(43)	8130(2)	8365(2)	6308(1)	39(1)
C(44)	6676(2)	5501(2)	6075(1)	27(1)
C(45)	4860(2)	7008(2)	5825(1)	29(1)

Table S18: Bond lengths [Å] and angles [°] for Rmsy2.

Zn-N(1)	1.9614(12)	C(16)-C(17)	1.441(2)
Zn-N(2)	1.9577(13)	C(18)-C(19)	1.510(2)
Zn-N(3)	1.9020(13)	C(19)-C(20)	1.398(2)
Si(1)-N(3)	1.7085(14)	C(20)-C(21)	1.398(2)
Si(1)-C(40)	1.8712(18)	C(21)-C(22)	1.507(2)
Si(1)-C(41)	1.8758(17)	C(23)-C(24)	1.369(2)
Si(1)-C(42)	1.8768(18)	C(23)-C(32)	1.434(2)
Si(2)-N(3)	1.7123(14)	C(24)-C(25)	1.413(2)
Si(2)-C(43)	1.8826(18)	C(25)-C(26)	1.359(3)
Si(2)-C(44)	1.8765(17)	C(26)-C(27)	1.410(3)
Si(2)-C(45)	1.8807(19)	C(27)-C(28)	1.409(3)
N(1)-C(16)	1.429(2)	C(27)-C(32)	1.443(2)
N(1)-C(19)	1.330(2)	C(28)-C(29)	1.350(3)
N(2)-C(21)	1.332(2)	C(29)-C(30)	1.405(3)
N(2)-C(23)	1.4305(19)	C(30)-C(31)	1.375(3)
C(1)-C(2)	1.511(2)	C(31)-C(32)	1.441(2)
C(2)-C(3)	1.387(2)	C(31)-C(33)	1.505(2)
C(2)-C(7)	1.395(2)	C(33)-C(34)	1.387(3)
C(3)-C(4)	1.393(2)	C(33)-C(38)	1.378(3)
C(4)-C(5)	1.391(2)	C(34)-C(35)	1.392(3)
C(5)-C(6)	1.395(2)	C(35)-C(36)	1.384(3)
C(5)-C(8)	1.503(2)	C(36)-C(37)	1.379(3)
C(6)-C(7)	1.387(2)	C(36)-C(39)	1.511(2)
C(8)-C(9)	1.382(2)	C(37)-C(38)	1.392(3)
C(8)-C(17)	1.439(2)		
C(9)-C(10)	1.408(2)	N(2)-Zn-N(1)	96.40(5)
C(10)-C(11)	1.360(3)	N(3)-Zn-N(1)	133.42(6)
C(11)-C(12)	1.419(2)	N(3)-Zn-N(2)	130.07(6)
C(12)-C(13)	1.413(2)	N(3)-Si(1)-C(40)	107.91(8)
C(12)-C(17)	1.440(2)	N(3)-Si(1)-C(41)	112.30(7)
C(13)-C(14)	1.369(2)	N(3)-Si(1)-C(42)	114.44(8)
C(14)-C(15)	1.403(2)	C(40)-Si(1)-C(41)	106.99(8)
C(15)-C(16)	1.373(2)	C(40)-Si(1)-C(42)	106.39(9)

C(41)-Si(1)-C(42)	108.39(9)
N(3)-Si(2)-C(43)	114.34(8)
N(3)-Si(2)-C(44)	111.11(7)
N(3)-Si(2)-C(45)	110.13(8)
C(44)-Si(2)-C(43)	105.73(9)
C(44)-Si(2)-C(45)	109.94(8)
C(45)-Si(2)-C(43)	105.34(10)
C(16)-N(1)-Zn	116.04(10)
C(19)-N(1)-Zn	122.24(10)
C(19)-N(1)-C(16)	121.14(13)
C(21)-N(2)-Zn	120.27(10)
C(21)-N(2)-C(23)	120.39(13)
C(23)-N(2)-Zn	117.73(10)
Si(1)-N(3)-Zn	115.46(7)
Si(1)-N(3)-Si(2)	126.01(8)
Si(2)-N(3)-Zn	117.80(7)
C(3)-C(2)-C(1)	121.49(17)
C(3)-C(2)-C(7)	117.55(16)
C(7)-C(2)-C(1)	120.95(16)
C(2)-C(3)-C(4)	121.21(16)
C(5)-C(4)-C(3)	121.10(15)
C(4)-C(5)-C(6)	117.81(15)
C(4)-C(5)-C(8)	119.73(14)
C(6)-C(5)-C(8)	122.18(14)
C(7)-C(6)-C(5)	120.79(15)
C(6)-C(7)-C(2)	121.51(15)
C(9)-C(8)-C(5)	114.80(14)
C(9)-C(8)-C(17)	119.02(14)
C(17)-C(8)-C(5)	126.17(14)
C(8)-C(9)-C(10)	122.51(16)
C(11)-C(10)-C(9)	119.63(15)
C(10)-C(11)-C(12)	120.88(15)
C(11)-C(12)-C(17)	120.01(15)
C(13)-C(12)-C(11)	118.86(15)
C(13)-C(12)-C(17)	121.13(14)
C(14)-C(13)-C(12)	120.81(15)
C(13)-C(14)-C(15)	119.05(16)
C(16)-C(15)-C(14)	122.34(15)
N(1)-C(16)-C(17)	122.60(14)
C(15)-C(16)-N(1)	116.48(14)
C(15)-C(16)-C(17)	120.67(14)
C(8)-C(17)-C(12)	117.90(14)
C(8)-C(17)-C(16)	126.10(14)
C(12)-C(17)-C(16)	115.99(14)
N(1)-C(19)-C(18)	119.69(15)
N(1)-C(19)-C(20)	123.02(14)
C(20)-C(19)-C(18)	117.27(15)
C(21)-C(20)-C(19)	128.95(16)
N(2)-C(21)-C(20)	123.44(15)
N(2)-C(21)-C(22)	119.06(14)
C(20)-C(21)-C(22)	117.49(15)
N(2)-C(23)-C(32)	121.57(14)
C(24)-C(23)-N(2)	117.48(14)
C(24)-C(23)-C(32)	120.72(14)
C(23)-C(24)-C(25)	121.96(16)
C(26)-C(25)-C(24)	119.10(16)
C(25)-C(26)-C(27)	121.16(15)
C(26)-C(27)-C(32)	120.63(16)
C(28)-C(27)-C(26)	119.06(16)
C(28)-C(27)-C(32)	120.31(17)
C(29)-C(28)-C(27)	121.08(18)
C(28)-C(29)-C(30)	119.4(2)
C(31)-C(30)-C(29)	123.0(2)
C(30)-C(31)-C(32)	118.77(16)
C(30)-C(31)-C(33)	113.87(16)
C(32)-C(31)-C(33)	127.35(15)
C(23)-C(32)-C(27)	116.34(15)
C(23)-C(32)-C(31)	126.32(14)
C(31)-C(32)-C(27)	117.33(15)
C(34)-C(33)-C(31)	121.12(18)
C(38)-C(33)-C(31)	120.77(18)
C(38)-C(33)-C(34)	117.24(17)

C(33)-C(34)-C(35)	121.5(2)	C(37)-C(36)-C(39)	121.52(19)
C(36)-C(35)-C(34)	121.18(19)	C(36)-C(37)-C(38)	121.9(2)
C(35)-C(36)-C(39)	121.39(18)	C(33)-C(38)-C(37)	121.1(2)
C(37)-C(36)-C(35)	117.07(17)		

Table S19: Anisotropic displacement parameters ($Å^2 \times 10^3$) for Rmsy2. The anisotropic displacement factor exponent takes the form: $-2\pi^2 [h^2 a^{*2} U^{11} + ... + 2 h k a^* b^* U^{12}]$.

	U^{11}	U^{22}	U^{33}	U^{23}	U^{13}	U^{12}
Zn	20(1)	13(1)	19(1)	5(1)	5(1)	7(1)
Si(1)	20(1)	17(1)	20(1)	4(1)	2(1)	8(1)
Si(2)	24(1)	16(1)	21(1)	5(1)	7(1)	4(1)
N(1)	23(1)	14(1)	26(1)	6(1)	9(1)	9(1)
N(2)	25(1)	15(1)	22(1)	6(1)	8(1)	11(1)
N(3)	22(1)	17(1)	22(1)	5(1)	5(1)	4(1)
C(1)	28(1)	47(1)	52(1)	21(1)	4(1)	19(1)
C(2)	24(1)	31(1)	33(1)	12(1)	6(1)	13(1)
C(3)	21(1)	24(1)	35(1)	8(1)	4(1)	6(1)
C(4)	25(1)	18(1)	31(1)	9(1)	7(1)	8(1)
C(5)	22(1)	20(1)	22(1)	7(1)	6(1)	9(1)
C(6)	22(1)	19(1)	29(1)	10(1)	6(1)	7(1)
C(7)	27(1)	23(1)	35(1)	14(1)	8(1)	13(1)
C(8)	21(1)	14(1)	26(1)	9(1)	7(1)	7(1)
C(9)	27(1)	23(1)	23(1)	6(1)	6(1)	11(1)
C(10)	33(1)	30(1)	27(1)	6(1)	11(1)	20(1)
C(11)	26(1)	27(1)	30(1)	10(1)	10(1)	17(1)
C(12)	22(1)	17(1)	26(1)	9(1)	8(1)	10(1)
C(13)	27(1)	22(1)	28(1)	10(1)	5(1)	13(1)
C(14)	36(1)	24(1)	25(1)	7(1)	1(1)	15(1)
C(15)	38(1)	19(1)	24(1)	6(1)	8(1)	16(1)
C(16)	24(1)	14(1)	26(1)	9(1)	9(1)	9(1)
C(17)	21(1)	13(1)	24(1)	9(1)	8(1)	7(1)
C(18)	32(1)	17(1)	47(1)	12(1)	21(1)	11(1)
C(19)	27(1)	14(1)	34(1)	6(1)	14(1)	8(1)
C(20)	22(1)	17(1)	46(1)	7(1)	13(1)	7(1)
C(21)	24(1)	20(1)	35(1)	9(1)	13(1)	12(1)
C(22)	26(1)	24(1)	66(1)	12(1)	13(1)	14(1)
C(23)	22(1)	17(1)	28(1)	8(1)	9(1)	11(1)
C(24)	39(1)	22(1)	28(1)	10(1)	14(1)	17(1)

C(25)	41(1)	22(1)	39(1)	18(1)	17(1)	18(1)
C(26)	26(1)	16(1)	43(1)	10(1)	11(1)	12(1)
C(27)	26(1)	17(1)	36(1)	4(1)	8(1)	10(1)
C(28)	66(1)	16(1)	43(1)	0(1)	11(1)	15(1)
C(29)	134(3)	26(1)	32(1)	-5(1)	13(1)	27(1)
C(30)	119(2)	26(1)	26(1)	5(1)	13(1)	24(1)
C(31)	46(1)	18(1)	25(1)	3(1)	6(1)	11(1)
C(32)	23(1)	15(1)	28(1)	5(1)	8(1)	9(1)
C(33)	48(1)	19(1)	17(1)	4(1)	6(1)	11(1)
C(34)	41(1)	30(1)	50(1)	21(1)	24(1)	16(1)
C(35)	45(1)	34(1)	46(1)	22(1)	20(1)	21(1)
C(36)	51(1)	20(1)	20(1)	6(1)	-2(1)	10(1)
C(37)	49(1)	22(1)	73(2)	16(1)	-32(1)	-3(1)
C(38)	53(1)	23(1)	67(2)	14(1)	-26(1)	6(1)
C(39)	56(1)	23(1)	38(1)	12(1)	-9(1)	8(1)
C(40)	31(1)	34(1)	22(1)	8(1)	4(1)	14(1)
C(41)	34(1)	21(1)	30(1)	3(1)	1(1)	12(1)
C(42)	24(1)	42(1)	34(1)	4(1)	2(1)	16(1)
C(43)	41(1)	26(1)	32(1)	5(1)	15(1)	-5(1)
C(44)	25(1)	26(1)	32(1)	6(1)	9(1)	11(1)
C(45)	41(1)	25(1)	24(1)	7(1)	5(1)	17(1)

Table S20: Hydrogen coordinates (x 10^4) and isotropic displacement parameters (Å^2 x 10^3) for Rmsy2.

	x	y	z	U_{eq}
H(1A)	-2073	2002	4776	61
H(1B)	-1000	3421	4693	61
H(1C)	-1682	3183	5424	61
H(3)	-1086	790	5438	34
H(4)	945	533	5939	29
H(6)	3586	4230	5904	28
H(7)	1559	4475	5396	32
H(9)	3567	1055	5307	29
H(10)	5574	591	5591	33
H(11)	6972	1347	6743	30
H(13)	7459	2462	7979	29
H(14)	6952	3676	8895	34
H(15)	5050	4281	8636	30
H(18A)	1798	1450	7312	45
H(18B)	305	1491	7494	45
H(18C)	1687	1938	8131	45
H(20)	-98	3142	7255	34
H(22A)	-173	5923	7690	55
H(22B)	-1096	4489	7248	55
H(22C)	-204	5608	6832	55
H(24)	2619	7469	6700	33
H(25)	3095	9642	6857	36
H(26)	3474	10787	8015	32
H(28)	3536	10912	9303	52
H(29)	3417	10013	10327	81
H(30)	2824	7835	10216	71
H(34)	4163	6183	9556	45
H(35)	3413	4114	9774	45
H(37)	-804	3377	9116	68

H(38)	-55	5431	8875	64
H(39A)	1488	1890	9556	64
H(39B)	-192	1596	9291	64
H(39C)	545	2182	10128	64
H(40A)	5394	7555	8841	43
H(40B)	7018	8216	9312	43
H(40C)	6337	6753	8908	43
H(41A)	7614	10031	7587	43
H(41B)	7758	10320	8455	43
H(41C)	6163	9618	7956	43
H(42A)	9160	7540	8267	50
H(42B)	9672	8991	8656	50
H(42C)	9611	8652	7796	50
H(43A)	8058	9160	6482	59
H(43B)	8221	8301	5791	59
H(43C)	8999	8367	6590	59
H(44A)	7708	5669	6216	41
H(44B)	6413	5293	5542	41
H(44C)	6038	4778	6281	41
H(45A)	3924	6333	5890	43
H(45B)	4988	6875	5315	43
H(45C)	4853	7844	5956	43

7 References

(1) Yang, R.; Xu, G.; Lv, C.; Dong, B.; Zhou, L.; Wang, Q. Zn(HMDS)$_2$ as a Versatile Transesterification Catalyst for Polyesters Synthesis and Degradation toward a Circular Materials Economy Approach. *ACS Sustain. Chem. Eng.* **2020**, *8*, 18347–18353.
(2) Young, M. S.; Rusconi, Y.; LaPointe, A. M.; Keresztes, I.; Talarico, G.; Coates, G. W. Leveraging Stereochemistry to Optimize the Properties of Polyhydroxyalkanoates. *J. Am. Chem. Soc. J. Am. Chem. Soc.* **2026**, *148*, 26768–26773.
(3) Zhou, Z.; LaPointe, A. M.; Shaffer, T. D.; Coates, G. W. Nature-Inspired Methylated Polyhydroxybutyrates from C1 and C4 Feedstocks. *Nat. Chem.* **2023**, *15*, 856–861.
(4) CrysAlisPro; Rigaku OD, The Woodlands, TX, 2015.
(5) Sheldrick, G. M. SHELXT - Integrated Space-Group and Crystal-Structure Determination. *Acta Cryst.* **2015**, *A71*, 3–8.
(6) Sheldrick, G. M. A Short History of SHELX. *Acta Cryst.* **2008**, *A64*, 112–122.
(7) Müller, P. Practical Suggestions for Better Crystal Structures. *Crystallogr. Rev.* **2009**, *15*, 57–83.
(8) Odian, G. G. *Principles of Polymerization,* 4th ed.; Wiley-Interscience: Hoboken, N.J, 2004.
(9) Hessler, J. P. The Use of Monte Carlo Simulations to Evaluate Kinetic Data and Analytic Approximations. *Int. J. of Chem. Kinet.* **1997**, *29*, 803–817.
(10) Press, W. H.; Teukolsky, S. A.; Vetterling, W. T.; Flannery, B. P. *Numerical Recipes: The Art of Scientific Computing,* 3rd ed.; Cambridge University Press: Cambridge, UK, 2007.
(11) Adamo, C.; Barone, V. Toward Reliable Density Functional Methods without Adjustable Parameters: The PBE0 Model. *J. Chem. Phys.* **1999**, *110*, 6158–6170.
(12) Grimme, S.; Antony, J.; Ehrlich, S.; Krieg, H. A Consistent and Accurate Ab Initio Parametrization of Density Functional Dispersion Correction (DFT-D) for the 94 Elements H-Pu. *J. Chem. Phys.* **2010**, *132*, 154104.
(13) Wadt, W. R.; Hay, P. J. Ab Initio Effective Core Potentials for Molecular Calculations. Potentials for Main Group Elements Na to Bi. *J. Chem. Phys.* **1985**, *82*, 284–298.
(14) Binkley, J. S.; Pople, J. A.; Hehre, W. J. Self-Consistent Molecular Orbital Methods. 21. Small Split-Valence Basis Sets for First-Row Elements. *J. Am. Chem. Soc.* **1980**, *102*, 939–947.
(15) McLean, A. D.; Chandler, G. S. Contracted Gaussian Basis Sets for Molecular Calculations. I. Second Row Atoms, Z = 11–18. *J. Chem. Phys.* **1980**, *72*, 5639–5648.
(16) Lee, C.; Yang, W.; Parr, R. G. Development of the Colle-Salvetti Correlation-Energy Formula into a Functional of the Electron Density. *Phys. Rev. B* **1988**, *37*, 785–789.
(17) Weigend, F.; Ahlrichs, R. Balanced Basis Sets of Split Valence, Triple Zeta Valence and Quadruple Zeta Valence Quality for H to Rn: Design and Assessment of Accuracy. *Phys. Chem. Chem. Phys.* **2005**, *7*, 3297–3305.
(18) Zhao, Y.; Truhlar, D. G. The M06 Suite of Density Functionals for Main Group Thermochemistry, Thermochemical Kinetics, Noncovalent Interactions, Excited States, and Transition Elements: Two New Functionals and Systematic Testing of Four M06-Class Functionals and 12 Other Functionals. *Theor. Chem. Acc.* **2008**, *120*, 215–241.
(19) Chai, J.-D.; Head-Gordon, M. Long-Range Corrected Hybrid Density Functionals with Damped Atom–Atom Dispersion Corrections. *Phys. Chem. Chem. Phys.* **2008**, *10*, 6615–6620.
(20) Boto, R. A.; Peccati, F.; Laplaza, R.; Quan, C.; Carbone, A.; Piquemal, J.-P.; Maday, Y.; Contreras-García, J. NCIPLOT4: Fast, Robust, and Quantitative Analysis of Noncovalent Interactions. *J. Chem. Theory Comput.* **2020**, *16*, 4150–4158.

(21) Humphrey, W.; Dalke, A.; Schulten, K. VMD: Visual Molecular Dynamics. *J. Mol. Graph.* **1996**, *14*, 33–38.
(22) Falivene, L.; Cao, Z.; Petta, A.; Serra, L.; Poater, A.; Oliva, R.; Scarano, V.; Cavallo, L. Towards the Online Computer-Aided Design of Catalytic Pockets. *Nat. Chem.* **2019**, *11*, 872–879.
(23) Bickelhaupt, F. M.; Houk, K. N. Analyzing Reaction Rates with the Distortion/Interaction-Activation Strain Model. *Angew. Chem., Int. Ed. Engl.* **2017**, *56*, 10070–10086.
